# Novel 5,6-dichlorobenzimidazole derivatives as dual BRAF_WT_ and BRAF_V600E_ inhibitors: design, synthesis, anti-cancer activity and molecular dynamics simulations

**DOI:** 10.1186/s13065-025-01402-8

**Published:** 2025-02-21

**Authors:** Ahmed Temirak, Ahmed M. El Kerdawy, Amira M. Nageeb, Heba T. Abdel-Mohsen

**Affiliations:** 1 Chemistry of Natural and Microbial Products Department, Pharmaceutical and Drug Industries Research Institute, National Research Centre, Dokki, P.O. 12622, Cairo, Egypt; 2 School of Health and Care Sciences, College of Health and Science, University of Lincoln, Joseph Banks Laboratories, Green Lane, Lincoln, UK; 3 Department of Pharmaceutical Chemistry, Faculty of Pharmacy, Cairo University, Kasr El-Aini Street, P.O. Box 11562, Cairo, Egypt; 4 High Throughput Molecular and Genetic Technology Lab, Center of Excellence for Advanced Sciences, Biochemistry Department, Biotechnology Research Institute, National Research Centre, Dokki, P.O. 12622, Cairo, Egypt

**Keywords:** Design, Dichlorobenzimidazole, BRAF, Anticancer, Molecular dynamic simulations

## Abstract

A new series of 1-substiuted-5,6-dichloro-2-(4-methoxyphenyl)-1*H*-benzo[*d*]imidazoles **10a**–**p** was designed and synthesized to target both BRAF_WT_ and BRAF_V600E_. The design strategy ensures that these derivatives would effectively occupy the ATP binding pocket of BRAF_WT/V600E_ kinase domains and extend over the gate area interacting through hydrogen bonding with the surrounding key amino acids Glu500 and Asp593 and to finally occupy the allosteric hydrophobic back pocket. Some synthesized derivatives demonstrated impressive potency against BRAF_WT_ with % inhibition approaching 91% at a concentration of 10 µM. The most potent candidate **10h** demonstrated IC_50_ values of 1.72 and 2.76 µM on BRAF_WT_ and BRAF_V600E_, respectively. At the same time, the synthesized benzimidazoles **10a**–**p** were examined for their growth inhibitory activity on NCI-60 cancer cell lines. Again, compound **10h** revealed a potent GI_50_ across a range of cancer cell lines. Moreover, it arrested cell cycle progression in HT29 colon cancer cell line at G2/M phase and induced apoptosis in the same cell line. Molecular dynamics simulations supported the validity of the design assumption, simultaneously, ADME prediction study displayed that the designed benzimidazoles exhibit promising physiochemical and drug-likeness properties as anticancer agents.

**Graphical Abstract:**

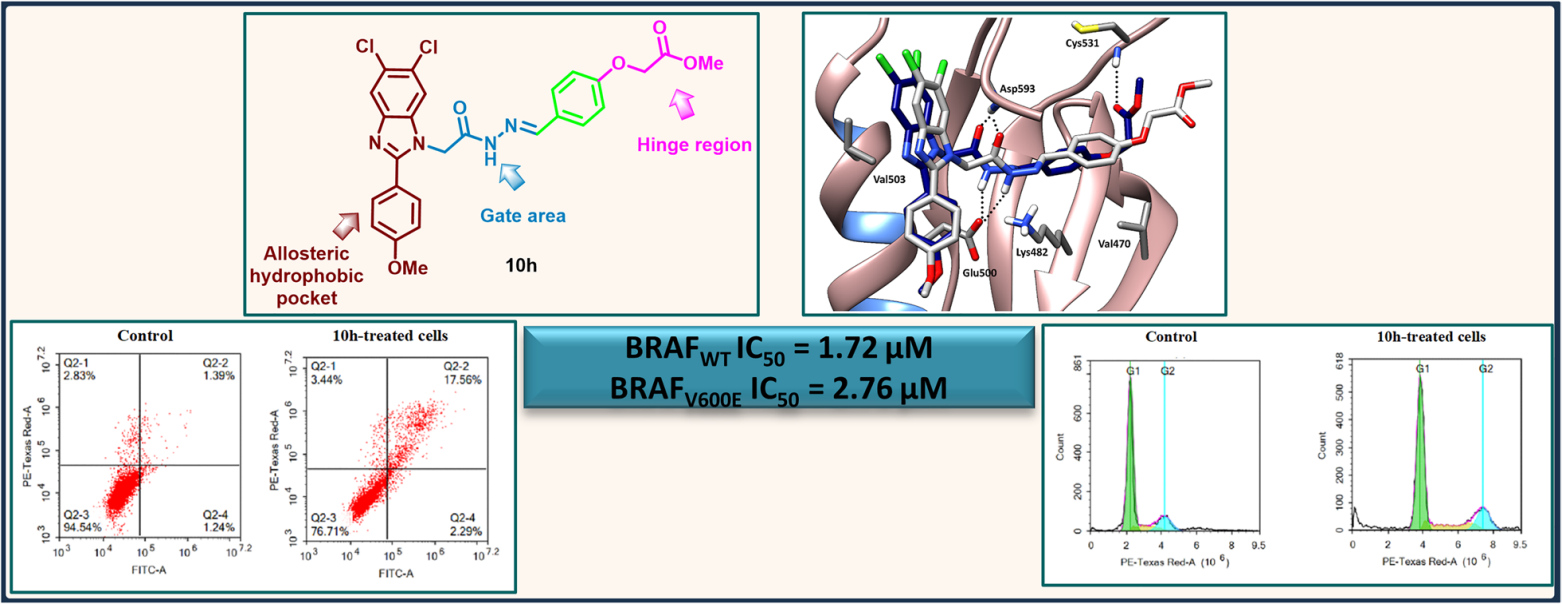

**Supplementary Information:**

The online version contains supplementary material available at 10.1186/s13065-025-01402-8.

## Introduction

A significant signal transduction pathway that controls cell proliferation and migration is the mitogen-activated protein kinase (MAPK) pathway or RAS-RAF-MEK pathway. This pathway is responsible for signal transfer to the DNA resulting in cell division and differentiation [1–6]. It is triggered by the activation of diverse membrane-bound tyrosine kinase receptors and G-protein-coupled receptors that result in turn in the activation of reticular activating system (RAS) and Rapidly Accelerated Fibrosarcoma (RAF) kinases [7]. Following the activation of RAF, MEK and ERK are phosphorylated and activated stimulating various nuclear and cytoplasmic molecules that are essential for cell survival, proliferation, and differentiation [6].

RAF is a serine-threonine kinase that is considered one of the most important targets in the RAS-RAF-MEK signaling pathway [7]. The RAF family contains three major isoenzymes; ARAF, BRAF, and CRAF, however, the BRAF is considered the most unregulated and the most susceptible to mutation leading to the aggressive growth and metastasis of cancer [6]. Mutation of the amino acid valine 600 to glutamic acid (V600E) is the most critical type of mutation in BRAF as it constitutes more than 90% of the observed mutations in BRAF and results in more than tenfold increase in the activity of BRAF compared to the wild type [8, 9]. This type of mutation is observed in diverse types of cancer including melanoma [10], colorectal cancer [11], papillary thyroid cancers [12], non-small-cell lung cancers (NSCLCs) [13] and hairy cell leukemia [14]. Thus, targeting BRAF_WT_ and its mutated form BRAF_V600E_ by small-molecule inhibitors is an interesting strategy to counteract tumor growth and metastasis [15–17].

**Fig. 1:**
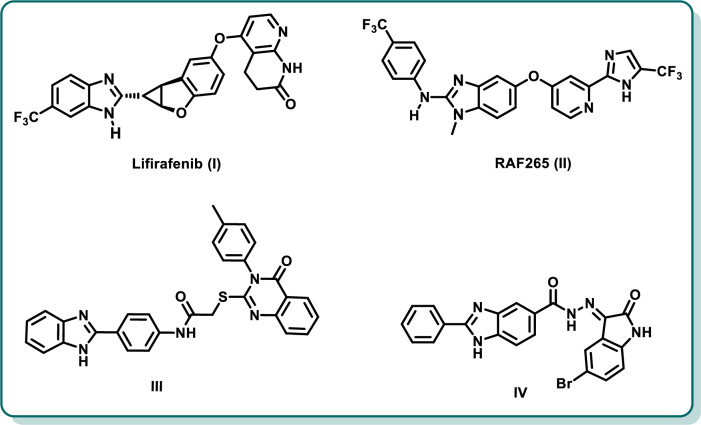
Structures of representative benzimidazole-based BRAF inhibitors **I**–**IV**

Benzimidazole is a privileged scaffold that was incorporated in diverse targeted chemotherapeutic agents due to its potent protein kinase inhibitory action [18–24]. In particular, several benzimidazole derivatives were recently highlighted as potent RAF kinase inhibitors [25–27]. Lifirafenib (**I**) is a benzimidazole derivative that demonstrated a potent inhibitory activity on RAF kinases, among others (EGFR_WT_ and EGFR_T790M/L858R_) (Fig. 1) [25]. Lifirafenib (**I**) is now in clinical trials for solid tumors possessing BRAF_V600E_ mutation, such as melanoma, mutated NSCLC, papillary thyroid cancer and ovarian cancer [28]. Besides, RAF265 (**II**) is another benzimidazole derivative that was reported to exhibit potent dual BRAF/VEGFR-2 inhibitory activity as well as potent antiproliferative activity against melanoma and colorectal cancer (Fig. 1) [29, 30]. Moreover, our research group has recently reported the design and synthesis of benzimidazole–quinazolinone conjugates as pan-RAF inhibitors and anticancer agents [26]. Compound **III** (Fig. 1) is a representative for this series which demonstrated a potent multi-kinase inhibitory activity against diverse oncokinases; VEGFR-2, BRAF_WT_, BRAF_V600E_, CRAF, PDGFR-β, FLT-3, and c-KIT with IC_50_ of 6.14, 6.74, 2.47, 10.83, 0.03, 0.13, and 0.12 µM, respectively. [26]. Recently, we reported a new series of 2,5-disubstituted benzimidazoles with a potent multi-kinase inhibitory activity [19, 27]. The representative benzimidazole-oxindole hybrid **IV** (Fig. 1) demonstrated potent IC_50_ values of 0.02, 1.52, 0.18 and 1.65 µM on BRAF_WT_, BRAF_V600E_, VEGFR-2, and FGFR-1 with promising in vitro and in vivo cytotoxic activity [19].

In view of the multiple and ongoing resistance of cancer cells to the current protein kinase inhibitors, there is a continuous need to discover new protein kinase inhibitors as targeted anticancer agents [31, 32].

**Fig. 2:**
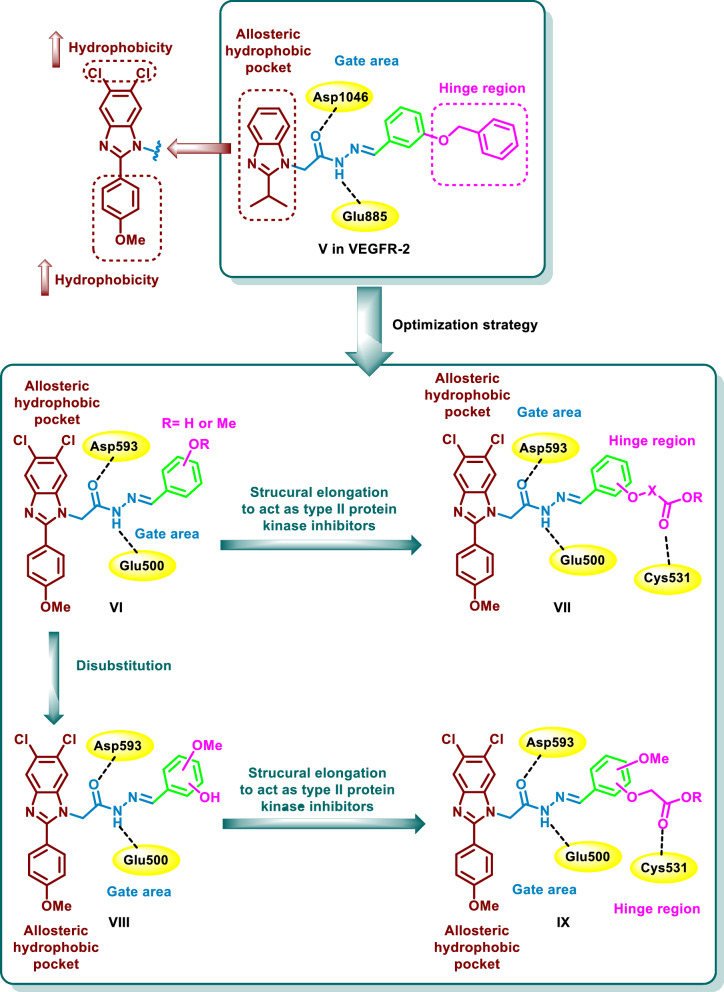
Design strategy of the novel 1-substituted-5,6-dichlorobenzimidazole derivatives **VI**–**IX**

Recently our group has published a series of 1,2-disubstituted benzimidazoles as type II VEGFR-2 inhibitors targeting hepatocellular carcinoma [33, 34]. Compound **V** was found to possess a potent inhibitory activity on VEGFR-2 (IC_50_ = 0.11 µM) as well as a potent antiproliferative activity on HepG2 cell line with IC_50_ value of 1.98 µM. In this series, molecular docking simulations showed that the benzimidazole moiety is accommodated in the allosteric hydrophobic back pocket of the VEGFR-2 kinase domain interacting through multiple hydrophobic interactions with the hydrophobic side chains of the surrounding residues [33, 34] (Fig. 2).

Based on the analogues binding sites of VEGFR-2 and BRAF, we were curious to optimize the previously designed scaffold for the design and synthesis of new benzimidazole derivatives as type II BRAF inhibitors. In reference to the previous results on one hand and the well-known pharmacophoric features of the type II BRAF inhibitors on the other hand [3, 27, 33, 34], we aim, in the current research, to enhance the binding to the kinase domain through increasing the hydrophobic interaction of the benzimidazole moiety with the allosteric hydrophobic back pocket [35]. This was achieved by its replacement with the more hydrophobic 5,6-dichlorobenzimidazole moiety which has a reported promising antiproliferative activity and kinase inhibitory activity [36, 37]. Thus, we have tailored a new series of 1-substituted-5,6-dichlorobenzimidazole derivatives **VI**–**IX** as BRAF inhibitors (Fig. 2). Our design approach was based on the accommodation of the 2-phenyl-5,6-dichlorobenzimidazole fragment in the allosteric hydrophobic back pocket of BRAF binding site in which the 5,6-dichloro moieties and the 4-methoxyphenyl moiety are assumed to stabilize the hydrophobic interactions with the amino acids lining this pocket. The *N*-1 of the benzimidazole scaffold was functionalized with an acetohydrazide moiety which is expected to get involved in hydrogen bonding interactions with the key amino acids Glu500, and Asp593 in the gate area. The hydrazide moiety was then functionalized with hydroxy or methoxyphenyl groups to give the general structure **VI** (Fig. 2). Further derivatization of the hydroxyphenyl group with acetic acid, methyl acetate ester, isopropionic acid, or ethyl isopropionate ester was performed in an attempt to achieve an interaction with the key amino acid Cys531 in the hinge region through hydrogen bonding (general structure **VII**) (Fig. 2). To establish a structure–activity relationship for this series, introduction of disubstituted hydroxy / methoxy phenyl groups was carried out to give the general structure **VIII**. Furthermore, further elongation of the hydroxy groups of **VIII** with acetic acid and its methyl ester was carried out to give the general structure **IX** (Fig. 2). The designed compounds were prepared and were subjected to biochemical evaluation of their inhibitory activity on BRAF_WT_ and of their antiproliferative activity on the cancer cell lines of NCI-60 panel. The most potent candidate was further evaluated for its inhibitory activity on BRAF_V600E_ as well as for its effect on cell cycle progression and cell apoptosis of HT29 cell line derived from colorectal cancer. Molecular docking and dynamic simulations on BRAF_WT/V600E_ were conducted to confirm the design approach. Finally, the ADME properties of the synthesized candidates were predicted using the SwissADME free web tool to anticipate their pharmacokinetics [38].

## Results and discussion

### Chemistry

**Scheme 1:**
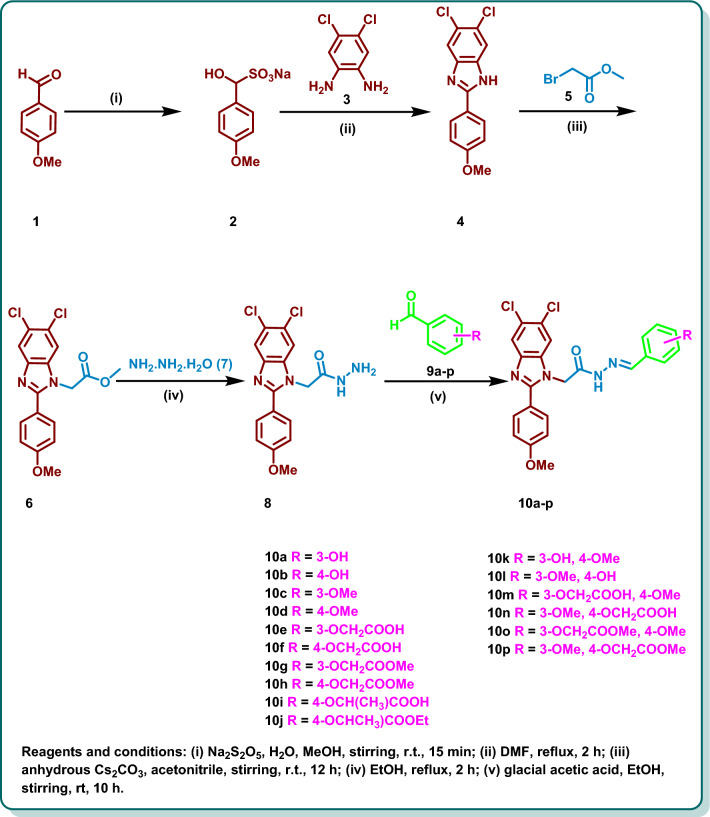
Synthesis of 1-substituted 5,6-dichlorobenzimidazoles **10a**–**p**

**Fig. 3:**
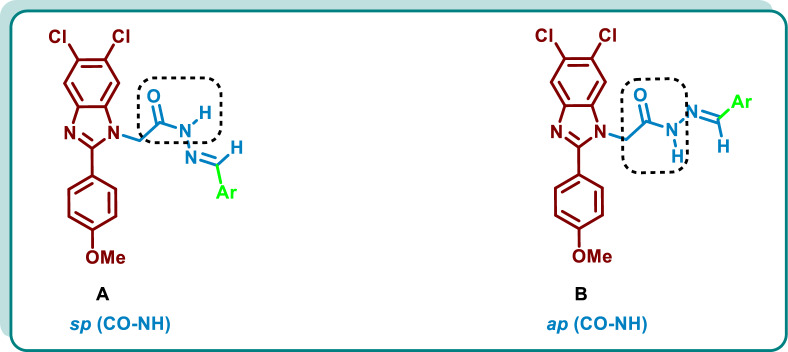
Structures of synperiplanar **A** and antiperiplanar **B** conformers

Synthesis of the target 5,6-dichlorobenzimidazoles **10a**–**p** was performed by initial formation of 4-methoxybenzaldehyde bisulfite adduct **2** through the reaction of 4-methoxy benzaldehyde (**1**) with sodium metabisulfite [33]. Then condensation of 4,5-dichloro-*o*-phenylene diamine (**3**) with **2** was carried out to yield the starting 5,6-dichlorobenzimidazole (**4**). The reaction of methyl bromoacetate (**5**) with **4** in the presence of Cs_2_CO_3_ resulted in the formation of **6** which was further reacted with hydrazine hydrate 98% (**7**) to afford the acid hydrazide derivative **8**. Acid catalysed condensation reaction of **8** with diverse aldehydes **9a-p** was performed to give the target dichlorobenzimidazole derivatives **10a**–**p** (Scheme 1). The NMR spectra of the synthesized candidates displayed the appearance of non-separated mixture of synperiplanar **A** and antiperiplanar conformers **B** in a ratio of ~ 1:3 due to rotation of C-N of CONH group [34, 39, 40] (Fig. 3) (For further details see the experimental part and the SI).

The structure of the final compounds **10a**–**p** was confirmed by several spectroscopic methods (IR, ^1^H NMR, ^13^C NMR and HRMS). The IR spectra of **10a**–**p** revealed distinct stretching vibrations. The carbonyl group of the acid hydrazide moiety displayed characteristic peaks in the range of ~ 1670–1690 cm⁻^1^. Similarly, the C=N bond of the benzimidazole ring exhibited peaks around ~ 1600–1610 cm⁻^1^. In addition, compounds with terminal carbonyl groups, such as the acid group in **10e** and the ester group in **10g**, showed prominent peaks between ~ 1730–1760 cm⁻^1^.

Additionally, ^1^H NMR and ^13^C NMR spectroscopy, providing detailed insights into their conformational properties, as mentioned earlier, the products **10a**–**p** exist as both major and minor conformers. In the ^1^H NMR spectrum of compound **10d**, a clear singlet at *δ*_H_ 3.80 ppm, integrating for six protons, corresponds to the two methoxy groups. Furthermore, another singlet is observed at *δ*_H_ 5.51 ppm, with an integration of two protons, representing the CH₂ group of the acid hydrazide moiety. In addition, the aromatic region reveals the presence of eight protons from the two phenyl rings, appearing as four distinct doublets at *δ*_H_ 6.99, 7.09, 7.65, and 7.66 ppm, respectively. Moreover, the two aromatic protons belonging to the benzimidazole ring are evident as singlets at *δ*_H_ 7.95 and 7.98 ppm. Subsequently, the CH proton of the imine group is identified as a singlet at *δ*_H_ 8.05 ppm. Finally, the NH proton of the acid hydrazide group is clearly detected as a singlet at *δ*_H_ 11.67 ppm. This comprehensive analysis confirms the expected structure of the compound **10d**.

From the HRMS analysis of our final compounds **10a**–**p**, detection occurred exclusively in the negative mode, likely due to the electron-rich nature of the Schiff bases, the presence of acidic hydrogens adjacent to the imine group, or the stabilization of the negative charge.

### Biological activity

#### Investigation of the inhibitory activity of 10a–p on BRAF_WT_ at 10 µM

The 1-substituted 5,6-dichlorobenzimidazoles **10a**–**p** were evaluated for their inhibitory activity on BRAF_wt_ at 10 micromolar concentration and the % of inhibition is shown in Table 1.

**Table 1:**
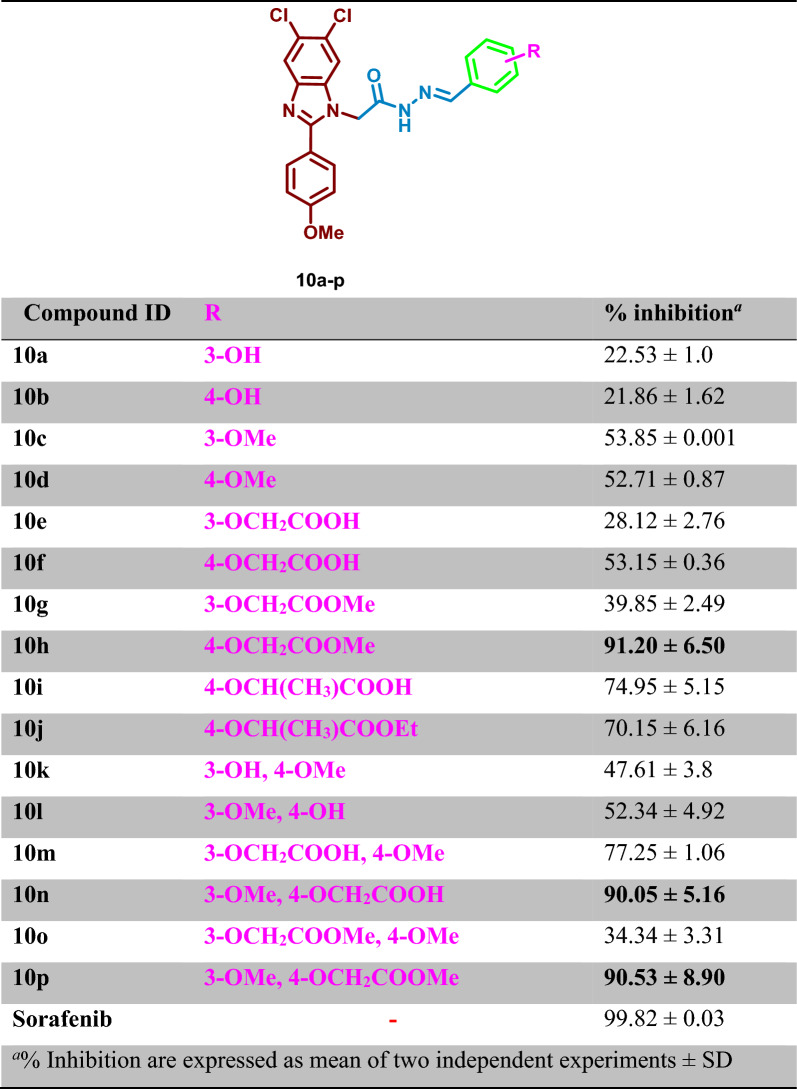
Percentage inhibition of BRAF_wt_ after treatment with the synthesized 1-substituted 5,6-dichlorobenzimidazoles **10a**–**p** at 10 µM

**Fig. 4:**
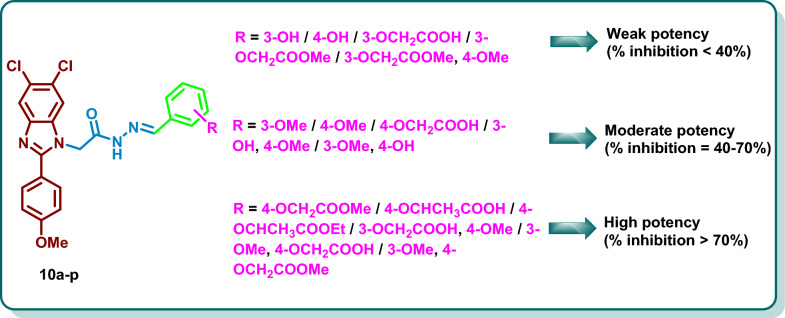
General structure activity relationship of inhibitory activity of **10a**–**p** on BRAF_WT_

The synthesized derivatives **10a**–**p** demonstrated diverse % of inhibition against BRAF_WT_ with weak to potent inhibitory activity reaching 91.20%. Analysis of the overall results revealed that derivatization of the 4 position of aryl spacer with acetic acid in **10f** and **10n**; acetic acid methyl ester in **10h** and **10p**; isopropionic acid in **10i** or its ethyl ester in **10j** demonstrated favourable activity with % inhibition ranging from 53.15 to 91.20% which can be attributed to the ability of the acid or the ester moiety to occupy the hinge region of the target kinases and involved in hydrogen bonding with the key amino acids. Further analysis demonstrated that the dichlorobenzimidazoles incorporating 3-hydroxyphenyl group **10a** or 4-hydroxyphenyl group **10b** showed weak inhibitory activity with % inhibition of 22.53% and 21.86%, respectively. Replacement of the 3-hydroxy and 4-hydroxy groups in **10a** and **10b** with 3-methoxy or 4-methoxy groups in **10c** and **10d**, respectively, resulted in moderate improvement in the % inhibition (53.85% and 52.71%, respectively). Derivatization of **10a** with acetic acid in **10e** or methyl acetate ester in **10g** resulted in a slight increase in the % inhibition (28.12% and 39.85%, respectively). Meanwhile, derivatization of **10b** with acetic acid in **10f** resulted in a moderate potency increase (% inhibition = 53.15%), moreover, methyl esterification of **10f** to afford **10h** demonstrated significant increase in potency (% inhibition = 91.20%). Structural elongation of **10b** using isopropionic acid in **10i** and ethyl isopropionate in **10j** afforded the same level of inhibitory activity (% inhibition of 74.95% and 70.15%, respectively). The disubstituted phenyl group in **10k** and **10l** demonstrated a moderate % inhibition of 47.61% and 52.34%, respectively. Derivatization of hydroxy groups in **10k** and **10l** with acetic acid moiety in **10m** and **10n** resulted in increasing the potency (% inhibitions of 77.25% and 90.05%, respectively). Methyl esterification of **10m** to afford **10o** resulted in decreasing the potency (% inhibition = 34.34%), while esterification of **10n** to yield **10p** showed the same level of inhibitory activity with % inhibition of 90.53% (Fig. 4).

#### Assessment of 10h on BRAF _WT_, BRAF_V600E_, VEGFR-2 and FGFR-1 at different concentrations

Based on the high potency of compound **10h**, it was further evaluated for its inhibitory activity at different concentrations on BRAF_WT_, BRAF_V600E_, VEGFR-2 and FGFR-1 and the IC_50_ (µM) was determined and presented in Table 2.

**Table 2:** IC_50_ (µM) of the 5,6-dichlorobenzimidazole **10h** on BRAF_WT_, BRAF_V600E_, VEGFR-2 and FGFR-1

Compound ID	IC_50_ (µM)			
	BRAF_WT_	BRAF_V600E_	VEGFR-2	FGFR-1
**10h**	1.72 ± 0.09	2.76 ± 0.15	1.52 ± 0.14	> 10
**Sorafenib**	0.02 ± 0.002	0.04 ± 0.009	0.10 ± 0.01	0.58 ± 0.10 [41]

^a^Results are mean of two independent experiments ± standard deviation (SD)

The findings in Table 2 demonstrated that compound **10h** displayed potent dual inhibitory activity on both BRAF_WT_ and BRAF_V600E_ with IC_50_ of 1.72 and 2.76 µM, respectively as well as promising inhibitory activity on VEGFR-2 with IC_50_ = 1.52 µM, whereas, IC_50_ > 10 µM was displayed against FGFR-1. These results could be considered as a promising activity in terms of multi-kinase activity against cancer-associated kinases.

#### Antiproliferative activity on NCI cancer cell lines at single concentration

In parallel the 1-substituted 5,6-dichlorobenzimidazoles **10a**–**p** were tested for their growth inhibitory activity on NCI cancer cell lines at 10 µM and the results are presented in Table 3.

**Table 3:** In vitro growth inhibition% (GI%) of NCI 60 cancer cell line panel after treatment with 10 μM of the 5,6-dichlorobenzimidazoles **10a-p**

Cell line	GI%															
	**10a**	**10b**	**10c**	**10d**	**10e**	**10f**	**10g**	**10h**	**10i**	**10j**	**10k**	**10l**	**10m**	**10n**	**10o**	**10p**
*Leukemia*																
CCRF-CEM	40.62	25.45	41.76	40.20	–^a^	–	20.86	28.15	10.70	25.77	43.81	46.87	–	–	16.92	21.09
HL-60(TB)	42.56	50.68	28.79	40.93	–	–	–	–	–	6.19	60.83	44.48	–	–	–	–
K-562	66.04	47.49	46.52	58.74	–	–	21.26	31.44	10.25	41.30	59.93	58.06	–	–	23.55	12.30
MOLT-4	70.41	77.22	70.28	68.89	–	–	30.43	53.25	–	53.22	76.06	70.80	–	10.12	40.40	13.66
RPMI-8226	67.37	62.07	47.32	64.94	–	–	–	–	–	40.17	38.79	48.26	–	–	17.93	15.71
SR	42.00	64.70	48.41	35.11	–	–	78.28	81.09	nd^b^	nd	52.23	49.15	–	9.65	90.91	92.81
*Non-small cell lung cancer*																
A549/ATCC	62.54	57.17	36.06	59.36	–	–	–	–	–	–	49.90	35.43	–	–	8.33	–
EKVX	76.64	52.61	30.42	76.83	–	5.96	13.17	–	20.53	21.66	56.44	51.93	–	–	13.97	18.90
HOP-62	24.15	19.34	6.68	23.60	–	–	–	–	–	–	–	12.36	–	–	–	–
HOP-92	23.80	40.04	20.63	10.87	–	–	5.50	7.02	6.61	15.15	12.02	40.09	–	–	29.13	–
NCI-H226	43.71	35.51	17.13	33.93	–	–	8.00	–	23.02	7.33	48.10	47.52	11.67	10.07	10.16	–
NCI-H23	53.77	20.26	10.24	50.92	5.12	–	–	–	13.85	20.83	21.10	15.00	8.00	9.67	7.03	–
NCI-H322M	nd	nd	nd	nd	–	nd	nd	nd	–	–	8.94	nd	–	–	11.69	nd
NCI-H460	46.80	49.72	23.69	47.16	–	–	–	–	–	–	24.82	31.02	–	–	–	–
NCI-H522	30.03	21.79	15.05	27.91	–	–	9.11	7.31	10.42	32.60	37.79	23.54	6.07	8.04	32.73	6.30
*Colon cancer*																
COLO 205	46.00	9.38	8.17	42.66	–	–	–	51.53	–	8.58	22.13	–	–	–	–	34.94
HCC-2998	35.88	21.40	7.72	34.90	–	–	44.05	141.67	–	15.41	32.16	18.19	–	–	83.83	86.06
HCT-116	59.65	45.12	37.87	61.29	–	–	10.61	10.84	11.45	32.30	21.96	31.62	–	6.00	22.87	25.15
HCT-15	59.96	52.32	44.18	62.86	–	6.73	21.44	40.33	21.36	37.10	47.22	51.50	–	–	23.63	21.46
HT29	41.21	25.09	5.29	37.47	–	–	51.70	93.39	–	39.16	30.24	12.99	–	–	90.31	116.43
KM12	41.00	26.33	17.64	40.78	–	–	–	–	–	–	20.12	25.45	–	–	–	–
SW-620	31.69	18.61	10.17	32.28	–	–	–	–	–	–	6.39	9.29	–	–	–	–
*CNS cancer*																
SF-268	51.51	52.55	48.31	48.62	–	–	–	–	–	14.30	45.59	53.72	–	–	18.59	–
SF-295	67.82	33.97	22.28	66.82	–	–	–	–	15.06	20.96	50.05	38.07	–	–	–	–
SF-539	36.49	32.53	24.42	34.24	–	10.80	26.46	39.16	–	–	29.09	32.40	–	–	80.00	100.82
SNB-19	50.38	39.01	32.43	50.46	–	–	–	8.19	5.79	11.18	40.26	37.31	–	–	–	–
SNB-75	nd	nd	nd	nd	–	nd	nd	nd	8.33	5.72	33.65	nd	–	–	27.54	nd
U251	39.33	42.76	26.55	38.63	–	–	–	10.10	7.22	5.26	37.98	25.82	–	–	6.53	–
*Melanoma*																
LOX IMVIL	28.97	42.62	30.32	24.66	–	8.41	19.05	39.53	10.92	18.37	38.37	38.55	–	10.35	36.36	25.23
MALME-3M	nd	nd	nd	nd	–	nd	nd	nd	–	–	23.45	nd	–	–	–	nd
M14	41.13	39.07	34.15	38.25	6.12	–	–	10.78	–	9.09	34.67	32.96	–	–	36.47	–
MDA-MB-435	45.87	39.15	33.04	41.42	–	–	11.95	18.29	7.59	24.76	46.82	42.16	–	–	45.50	31.92
SK-MEL-2	33.61	32.33	19.38	30.88	–	–	–	26.37	–	8.36	40.19	18.62	–	–	26.55	24.11
SK-MEL-28	39.28	26.44	20.46	33.50	–	–	–	31.67	–	–	17.31	7.48	–	–	–	6.03
SK-MEL-5	79.70	52.10	44.52	78.88	–	–	8.95	–	14.39	25.22	62.18	57.89	–	–	6.53	6.06
UACC-257	57.68	23.95	20.93	60.37	–	–	–	–	–	–	40.11	36.27	–	–	41.39	22.66
UACC-62	40.05	38.03	37.25	34.07	–	–	–	–	10.17	20.14	35.41	33.59	6.42	9.71	24.34	36.49
*Ovarian cancer*																
IGROV1	nd	nd	nd	nd	–	nd	nd	nd	–	25.42	10.22	nd	–	–	97.59	nd
OVCAR-3	36.61	27.01	14.42	22.72	–	–	–	93.05	–	–	23.32	28.67	–	–	–	–
OVCAR-4	68.08	52.79	42.13	64.98	–	–	–	–	–	9.77	47.33	52.90	–	–	18.01	–
OVCAR-5	20.54	–	–	21.49	–	–	–	–	–	–	–	–	–	–	–	–
OVCAR-8	29.48	28.04	13.46	22.40	–	–	84.17	148.5	–	20.67	29.33	27.13	–	–	69.76	143.03
NCI/ADR-RES	44.47	28.53	21.33	36.02	–	–	–	–	–	–	40.07	38.38	–	–	11.14	5.42
SK-OV-3	35.45	24.59	10.07	30.35	–	–	–	–	6.27	5.50	–	6.44	–	–	24.07	–
*Renal cancer*																
786-0	28.64	34.77	19.24	29.13	–	–	10.04	111.59	5.33	11.30	25.26	30.66	–	–	11.95	162.17
A498	48.93	25.86	22.72	47.19	–	–	–	–	–	–	29.67	16.16	–	–	–	–
ACHN	40.01	33.39	20.45	40.18	–	–	–	105.62	5.11	17.98	36.62	32.39	–	–	71.33	95.25
CAKI-1	54.98	57.23	43.50	52.05	–	15.02	10.48	5.89	10.19	9.10	48.25	55.32	–	–	37.02	16.49
RXF 393	44.37	41.22	23.07	38.74	–	–	32.92	85.73	–	43.72	42.17	32.23	–	–	129.37	186.24
SN12C	31.30	18.04	9.21	27.76	–	–	40.74	66.42	–	51.91	16.28	19.91	5.24	5.05	97.22	99.51
TK-10	37.25	13.91	5.88	34.89	–	–	–	–	–	–	28.04	16.23	–	–	–	58.05
UO-31	nd	nd	nd	nd	10.58	nd	nd	nd	25.68	25.57	55.02	nd	8.63	13.00	18.26	nd
*Prostate cancer*																
PC-3	57.75	40.68	29.47	68.47	–	8.65	36.91	74.39	19.27	38.51	56.64	54.19	–	10.81	51.23	79.77
DU-145	44.24	30.78	23.27	41.24	–	–	109.93	158.11	–	85.21	26.38	31.78	–	–	154.52	142.64
*Breast cancer*																
MCF7	80.56	74.15	56.58	81.47	13.62	18.91	31.90	67.54	28.47	39.00	68.03	65.94	9.09	14.21	33.41	25.80
MDA-MB-231/ATCC	19.97	21.19	15.05	27.38	–	–	21.57	94.55	12.19	17.19	33.90	17.17	–	11.50	90.11	64.62
HS 578T	36.85	37.75	28.95	34.63	7.11	–	13.87	72.23	–	–	27.43	32.68	–	6.21	127.85	117.72
BT-549	47.76	65.63	52.94	50.30	–	–	–	6.87	11.38	11.77	31.67	50.20	–	–	–	15.13
T-47D	69.74	56.35	45.77	67.21	–	–	55.88	106.75	27.22	72.82	43.35	51.28	–	–	106.11	81.14
MDA-MB-468	82.09	90.07	76.14	81.76	–	6.38	111.89	177.1	10.83	43.89	71.17	99.01	–	12.44	118.26	158.83
Mean GI%	46.85	38.52	28.07	44.60	–	–	14.62	36.65	–	18.44	35.35	34.64	–	–	34.57	37.62

^a^GI% < 5%

^b^Not detected

**Fig. 5:**
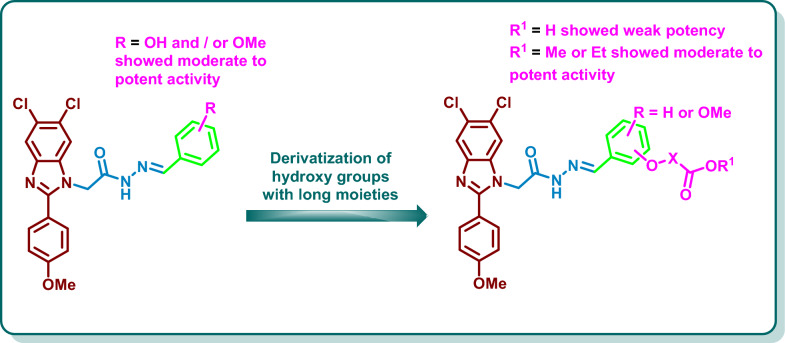
General structure–activity relationship of **10a**–**p** against NCI cancer cell lines

From the findings depicted in Table 3, it is clear that the nature of the substituent on the phenyl moiety has a diverse influence on the antiproliferative activity on the tested cell lines. The 5,6-dichlorobenzimidazole derivative **10a** incorporating 3-hydroxyphenyl group demonstrated moderate to potent inhibitory activity against NCI cancer cell lines with mean GI% = 46.85%. Shifting of the 3-hydroxy group to the 4-position in **10b** resulted in a decline in the potency (mean GI% of 38.52%). Replacement of the 3-hydroxyphenyl group in **10a** and 4-hydroxyphenyl group in **10b** with 3-methoxyphenyl and 4-methoxyphenyl groups in **10c** and **10d**, respectively, resulted in the same level of potency against the tested cell lines with mean growth inhibition % of 28.07 and 44.60%, respectively. Derivatization of 3-hydroxyphenyl and 4-hydroxyphenyl groups in **10a** and **10b** with acetic acid in **10e** and **10f** showed apparent decrease in the potency against nearly all the tested cell lines. On the contrary, the methyl esters **10g** and **10h** revealed preferable growth inhibitory activity against the tested cell lines with mean GI% of 14.62 and 36.65%. For the isopropionic acid derivatives **10i** and **10j**, the ethyl ester derivative **10j** displayed a higher potency than the acid derivative **10i** (mean GI% of 18.44% and less than 5%, respectively) (Fig. 4). Introducing disubstituted phenyl groups, viz*.* 3-hydroxy, 4-methoxy phenyl group in **10k** and 3-methoxy, 4-hydroxy phenyl group in **10l** resulted in the same level of potency against the tested cell lines with mean GI% of 35.35% and 34.64%, respectively. Derivatization of the hydroxy groups of **10k** and **10l** with acetic acid in **10m** and **10n** resulted in decreasing the potency against nearly all of the tested cell lines with mean GI% less than 5%, whereas the methyl esters **10o** and **10p** restored the potency against the tested cell lines with mean GI% of 34.57 and 37.62% (Fig. 5).

#### Examination of the antiproliferative activity of 10h, 10o and 10p on five dose level

Compounds **10h**, **10o** and **10p** were selected by NCI to be assayed for their activity on NCI cancer cell lines in 5-dose assay and the GI_50_ were depicted in Table 4.

**Table 4:** GI_50_ of the 5,6-dichlorobenzimidazole derivatives **10h**, **10o** and **10p** on NCI cancer cell lines

Cell name	GI_50_ (µM)		
	**10h**	**10o**	**10p**
*Leukemia*			
CCRF-CEM	4.78	> 100	> 72.5
HL-60(TB)	> 82.5	> 100	> 72.5
K-562	11.3	> 100	> 72.5
MOLT-4	5.36	14.2	> 72.5
RPMI-8226	71.2	> 100	> 72.5
SR	2.80	3.00	2.15
*Non-small cell lung cancer*			
A549/ATCC	> 82.5	> 100	> 72.5
EKVX	18.8	89.2	18.90
HOP-62	17.3	> 100	> 72.5
HOP-92	11.4	23.6	12.5
NCI-H226	> 82.5	> 100	> 72.5
NCI-H23	> 82.5	> 100	> 72.5
NCI-H322M	21.3	60.4	22.2
NCI-H460	> 82.5	> 100	26.1
NCI-H522	14.7	> 100	> 72.5
*Colon cancer*			
COLO 205	16.5	> 100	18.2
HCC-2998	13.1	13.4	11.7
HCT-116	68.0	> 100	> 72.5
HCT-15	11.2	> 100	> 72.5
HT29	1.79	12.9	1.46
KM12	> 82.5	> 100	> 72.5
SW-620	22.2	> 100	> 72.5
*CNS cancer*			
SF-268	10.1	> 100	32.4
SF-295	> 82.5	> 100	> 72.5
SF-539	10.7	5.28	7.52
SNB-19	> 82.5	> 100	> 72.5
SNB-75	2.14	14.4	1.73
U251	14.1	> 100	> 72.5
*Melanoma*			
LOX IMVI	6.83	> 100	31.2
MALME-3M	9.18	> 100	> 72.5
M14	17.5	> 100	> 72.5
MDA-MB-435	14.8	> 100	22.90
SK-MEL-2	10.5	21.6	10.0
SK-MEL-28	11.7	> 100	> 72.5
SK-MEL-5	80.9	> 100	> 72.5
UACC-257	12.7	38.7	14.70
UACC-62	10.1	14.5	6.52
*Ovarian cancer*			
IGROV1	4.06	14.7	3.60
OVCAR-3	10.6	> 100	> 72.5
OVCAR-4	17.2	20.5	23.0
OVCAR-5	34.3	> 100	24.0
OVCAR-8	2.93	8.68	2.54
NCI/ADR-RES	5.22	81.2	3.05
SK-OV-3	19.1	26.0	16.0
*Renal cancer*			
786-0	3.01	17.9	2.21
A498	15.4	> 100	11.6
ACHN	7.84	14.2	5.42
CAKI-1	5.74	15.3	2.77
RXF 393	2.85	11.0	1.43
SN12C	2.95	7.92	2.20
TK-10	13.2	18.3	12.6
UO-31	9.74	1.82	8.11
*Prostate cancer*			
PC-3	4.45	17.2	4.38
DU-145	1.67	4.79	1.90
*Breast cancer*			
MCF7	4.25	100	> 72.5
MDA-MB-231/ATCC	6.66	16.1	13.80
HS 578T	10.1	2.68	4.34
BT-549	25.6	> 100	> 72.5
T-47D	2.68	12.7	4.47
MDA-MB-468	1.68	4.40	1.48

Compound **10h** showed more potent activity against the tested cancer cell lines than **10o** and **10p**. Close analysis of the findings in Table 4 revealed that compound **10h** showed a potent GI_50_ of 4.78, 5.36 and 2.80 µM on the leukemia cell lines CCRF-CEM, MOLT-4, and SR, respectively. On colon cancer cell line HT29, it showed a GI_50_ of 1.79 µM and on CNS cancer cell line SNB-75, it showed a GI_50_ of 2.14 µM, whereas on melanoma cell lines LOXIMVI and MALME-3M, it showed GI_50_ of 6.83 and 9.18 µM, respectively. On Ovarian cancer cell lines IGROV1, OVCAR-8, and NCI/ADR-RES it exhibited GI_50_ of 4.06, 2.93, and 5.22 µM, respectively. Renal cancer cell lines 786-, ACHN, CAKI-1, RXF393, SN12C and UO-31 were also highly sensitive to **10h** showing GI_50_ of 3.01, 7.84, 5.74, 2.85, 2.95 and 9.74 µM, respectively. Moreover, for prostate cancer cell lines PC-3 and DU-145, GI_50_ of 4.45 and 1.67 µM, respectively, were observed. Finally, on the breast cancer cell lines MCF7, MDA-MB-231 / ATCC, T-47D, and MDA-MB-468, **10h** showed GI_50_ of 4.25, 6.66, 2.68, and 1.68 µM, respectively.

#### Evaluation of the antiproliferative activity of 10h on HSF normal cell line

Table 5 displays the findings of an analysis conducted on a normal human skin fibroblast (HSF) cell line to determine the cytotoxicity of the most powerful derivative, **10h** on a normal cell line. Remarkably, compound **10h** had no cytotoxic effect (IC_50_ > 100 µM) on the HSF cell line compared to IC_50_ = 2.25 µM for sorafenib.

**Table 5:** IC_50_ of **10h** on HSF cell line

Cell line	IC_50_ (µM)^a^	
	**10h**	**Sorafenib**
HSF	> 100	2.25 ± 0.14

^a^Data were expressed as mean of three independent ± standard deviation experiments

#### Cell cycle analysis

**Fig. 6:**
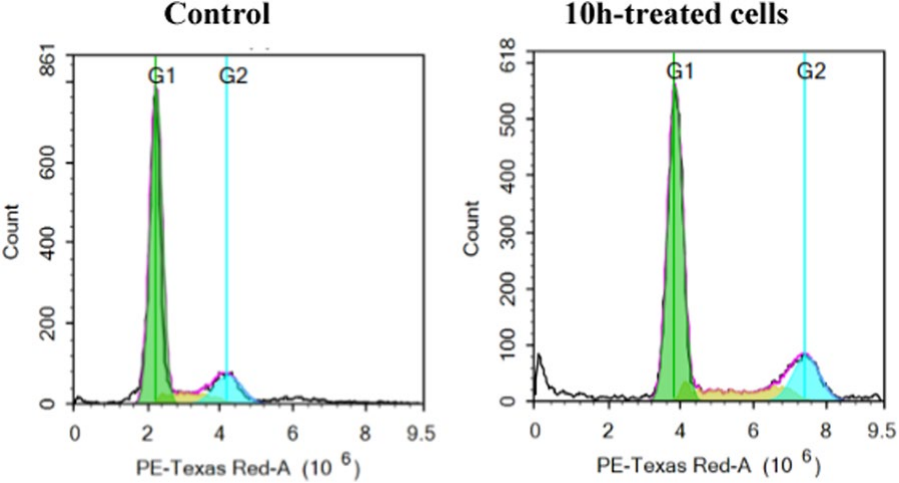
Investigation of the influence of **10h** on the progression of HT29 cell cycle

Motivated by the interesting inhibitory activity of **10h** on BRAF_WT_ and BRAF_V600E_ as well as its encouraging antiproliferative activity, it was hence selected to be examined further for its influence on the progression of the cell cycle of HT29 cell line derived from colorectal cancer which express BRAF_V600E_ [42] at its GI_50_ concentration and the results were depicted in Fig. 6 and Table 6. Interestingly, treatment with **10h** displayed apparent decrease in the cells accumulated in G1 phase from 75.44% in control cells to 62.69% in **10h-**treated cells. Besides, increase in the % of cells present in the S and G2 phase to 18.04 and 19.26%, respectively, in reference to 9.36 and 15.21%, respectively, in control cells. Additionally, it is interesting to note that cells accumulated in sub G1 phase appeared to increase after treatment with **10h** going from 3.06% (control) to 10.45% (**10h-**treated cells) indicating the apoptotic effect of the target compound.

**Table 6:** Percentages of different phases of HT29 cell cycle before and after treatment with **10h**

Comp	%G0/G1	%S	%G2/M	%Sub-G1
**Control**	75.44	9.36	15.21	3.06
**10h**	62.69	18.04	19.26	10.45

#### Apoptosis assay

**Fig. 7:**
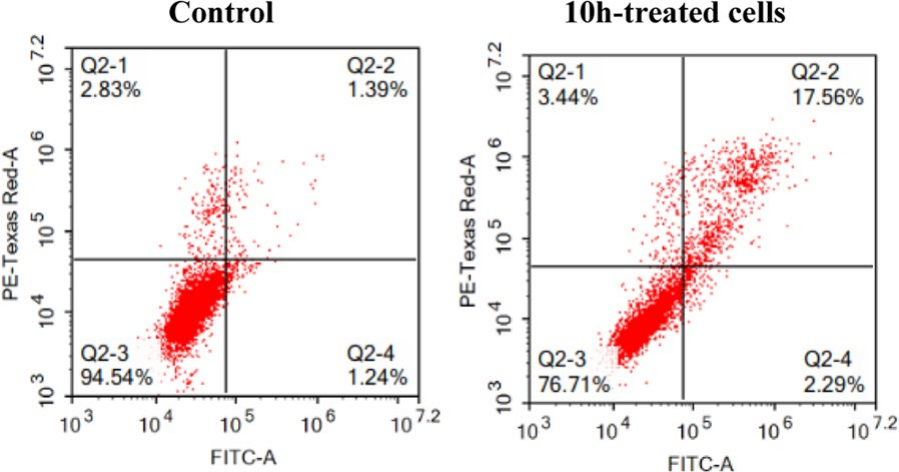
Percentage of cells in each phase before and after treatment with **10h** (Q2-3, viable; Q2-4, early apoptotic; Q2-2, late apoptotic; Q2-1, necrotic)

Furthermore, 5,6-dichlorobenzimidazole **10h** was further evaluated for its potency in inducing apoptosis in HT29 cell line at its GI_50_ concentration (Fig. 7). Analysis of the findings confirms the potential of **10h** to stimulate apoptosis in HT29 cell line as evidenced by the pronounced elevation in the total % of the cells in the apoptotic phases (Early and late) from 2.63% in control cells to 19.85% in **10h**-treated cells. Such that the % of cells accumulated in the early and late apoptosis phases increased from 1.24% and 1.39%, respectively, in control cells to 2.29% and 17.56%, respectively, in **10h**-treated cells. Moreover, the % of necrotic cells increased from 2.83% in control cells to 3.44% in **10h**-treated cells.

### Molecular modeling

The most promising compound **10h** was chosen as a representative compound for the newly synthesised compounds to investigate their binding pattern and dynamic behaviour in BRAF_WT_ and BRAF_V600E_ kinase domains utilising molecular dynamics (MD) simulations. Initially molecular docking was carried out to perform ligand placement of compound **10h** in the target kinase domains whose docking complexes were used as starting points for MD simulations [43]. The obtained poses in BRAF_WT_ and BRAF_V600E_ kinase domains were scored using Molecular Mechanics/Generalized Born Surface Area (MM/GBSA) binding free energy calculation [44].

#### Molecular docking

Molecular docking simulations using induced fit protocol implemented in MOE 2022.02 were initially used for ligand placement of compound **10h** in BRAF_WT_ and BRAF_V600E_ kinase domains using the protein structures PDB ID: 1UWH [45] and PDB ID: 1UWJ [45], respectively, co-crystallized with sorafenib which were first downloaded from the protein data bank [46].

The molecular docking setup was first validated by self-docking of sorafenib in BRAF_WT_ and BRAF_V600E_ kinase domains. The experimental ligands' binding pattern was accurately reproduced in the validation step, demonstrating the suitability of the docking protocol for the intended study. Such that the validation step revealed small RMSD values between the docking and the experimental ligand poses, BRAF_WT_ (0.730 Å) and BRAF_V600E_ (0.720 Å), moreover, the obtained docking poses replicated the key interactions performed by the co-crystalized ligand with the hot spots Glu500, Cys531, and Asp593 in both kinase domains (For further details see supporting materials). The validated molecular docking protocol was then used for ligand placement of compound **10h** in BRAF_WT_ and BRAF_V600E_ kinase domains (For details about the obtained **10h** placement poses, see the supporting materials).

#### Molecular dynamics simulations

MD simulations were performed using GROMACS 2021.3 package [43] for 100 ns using the molecular docking complexes of **10h** in the target kinases as starting points. Root mean square deviation (RMSD), root mean square fluctuation (RMSF) and radius of gyration (Rg) were used to assess the system stability and simulation quality.

**Fig. 8:**
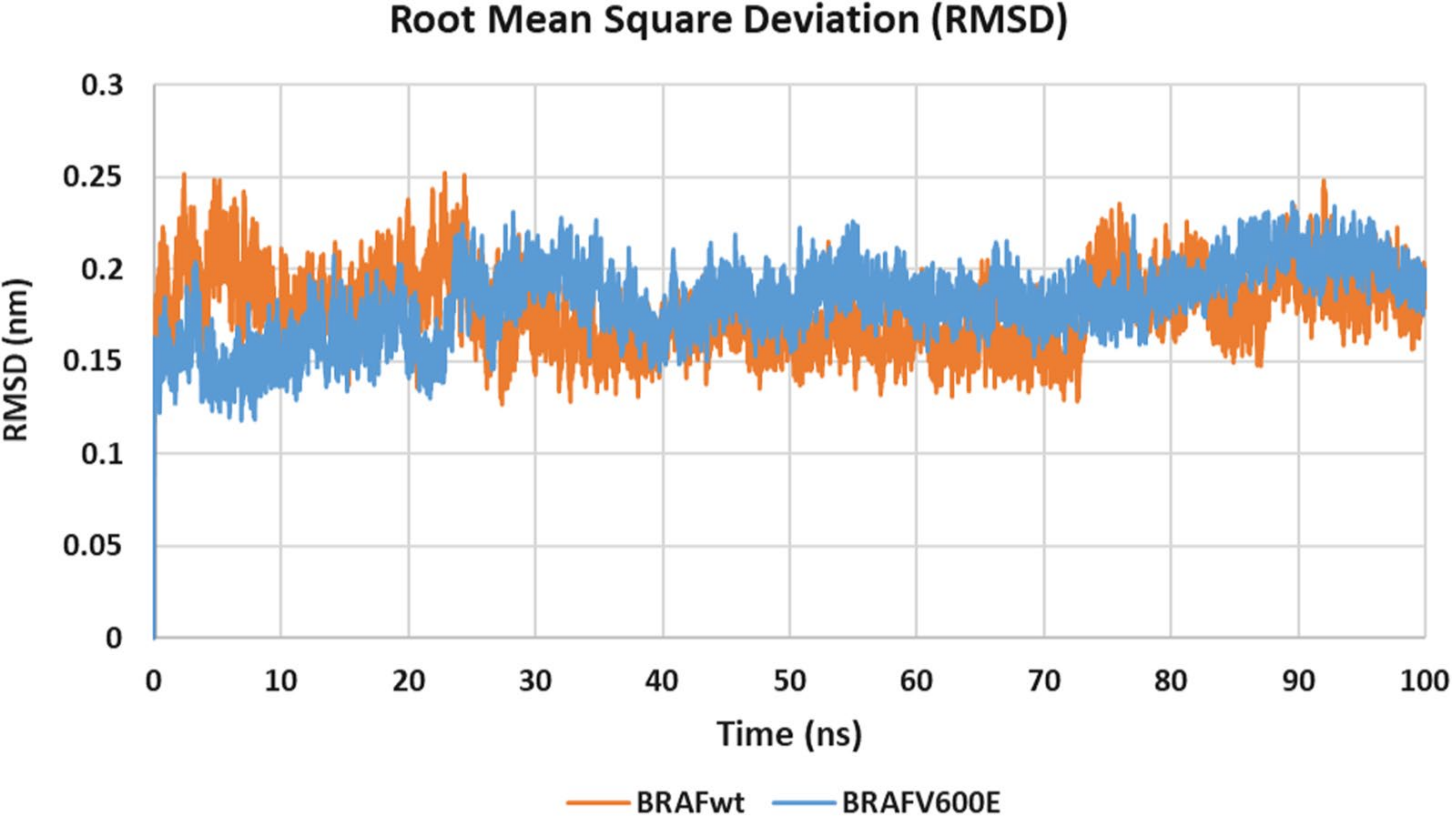
RMSD graph for the backbone atoms of **10h**/BRAF_WT_ (orange) and **10h**/BRAF_V600E_ (blue) structures from the initial reference frame backbone during 100 ns MD simulation

**Fig. 9:**
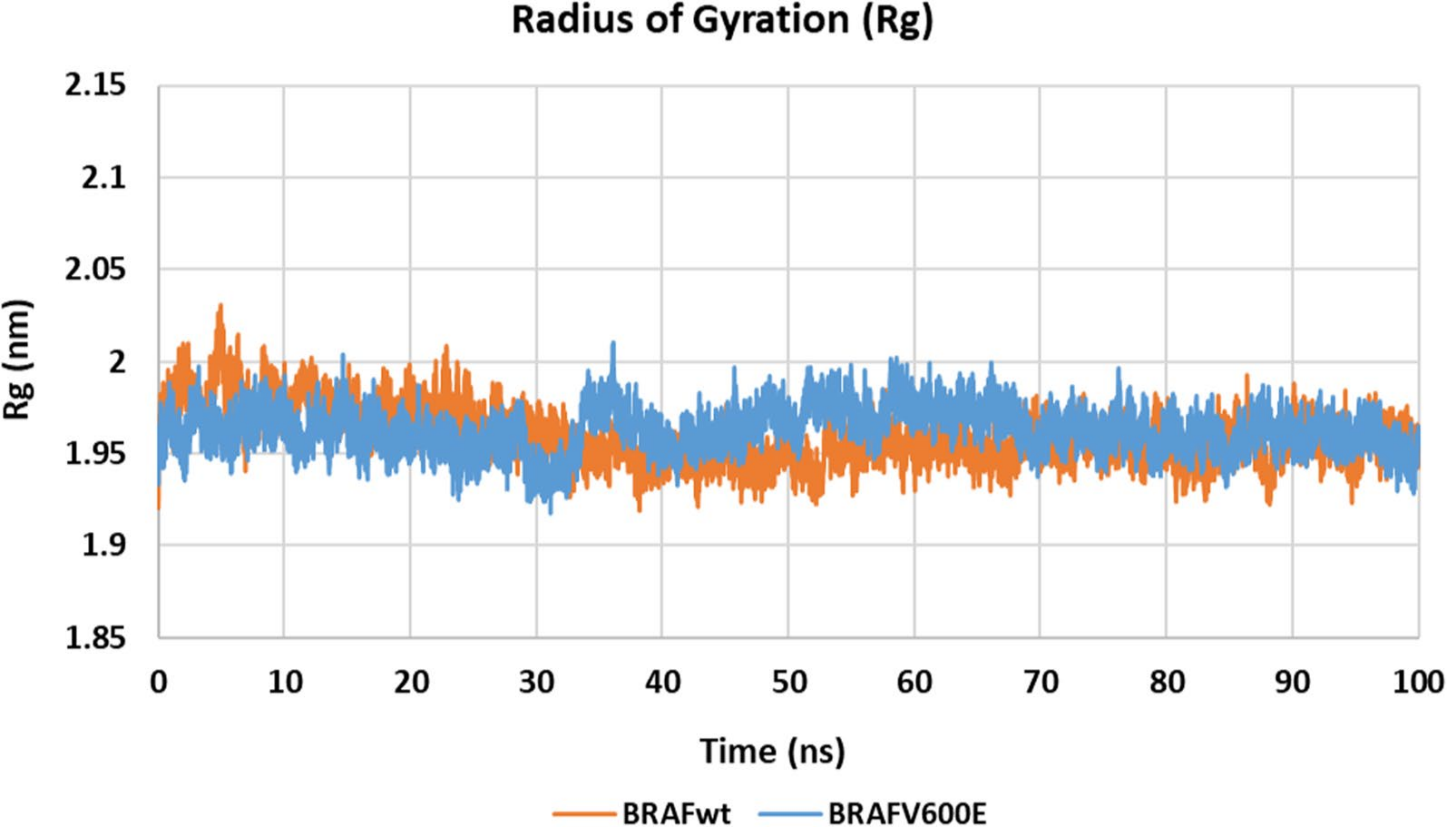
Radius of gyration (Rg) graph for **10h**/BRAF_WT_ (orange) and **10h**/BRAF_V600E_ (blue) structures during 100 ns MD simulation

Figure 8 shows that the RMSD values of **10h**/BRAF_WT/V600E_ structures stabilize at 25 ns showing an acceptable average RMSD of 0.177 and 0.182 nm, respectively. Furthermore, the stable radius of gyration Rg (< 2.0 nm) for both structures indicated that both systems are well-compacted throughout the simulation (Fig. 9).

Root Mean Square Fluctuation (RMSF) describes residues’ flexibility throughout the simulation [26]. Figure 9 shows that except for the terminal residues and loop regions, RMSF values of most residues have not exceeded 0.1 nm in both kinases. Furthermore, apart from the loop region Asp593-Ser621, the different amino acids in both kinases show a similar fluctuation pattern (Fig. 9).

**Fig. 10:**
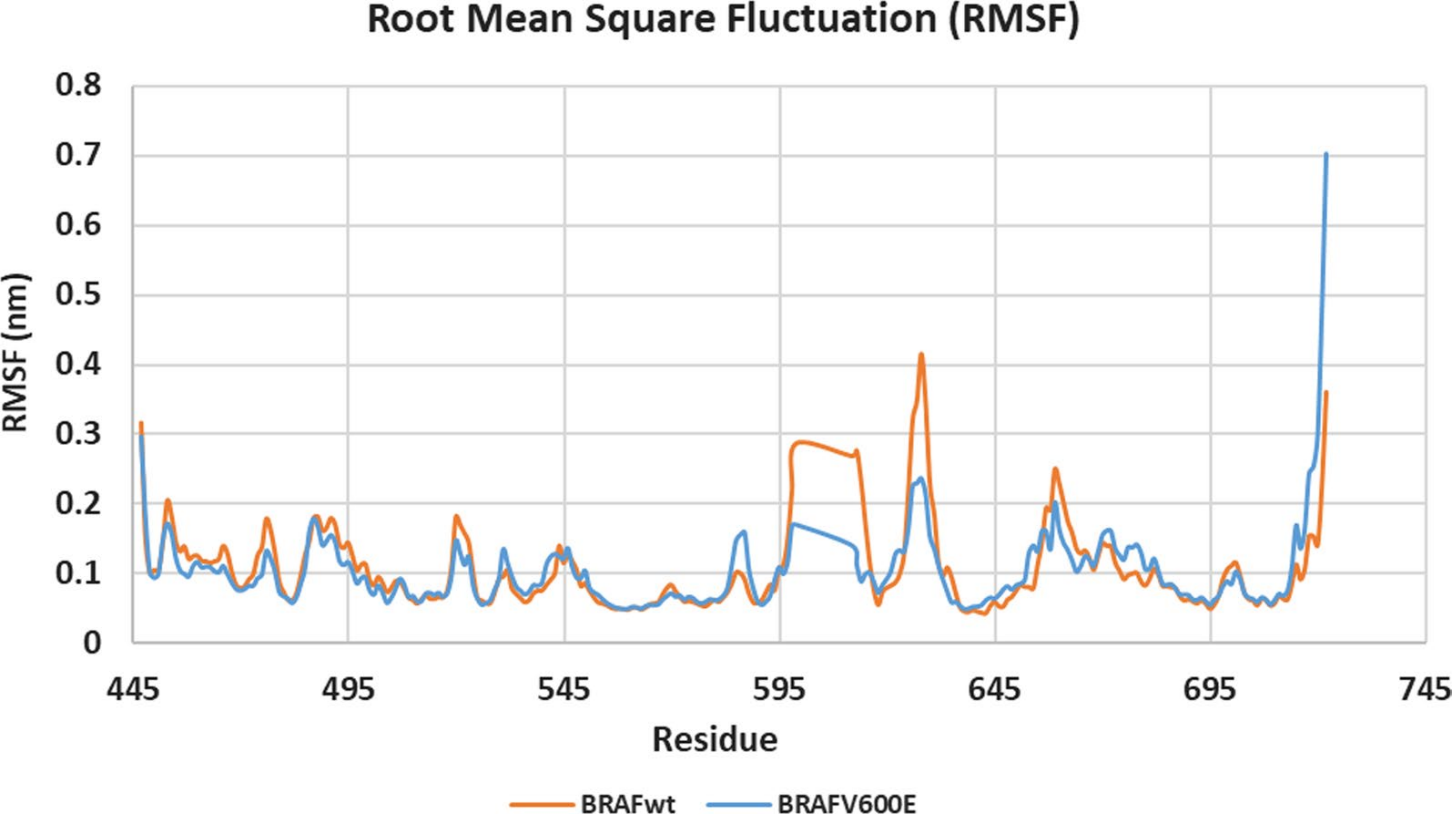
RMSF graph for the residues of **10h**/BRAF_WT_ (orange) and **10h**/BRAF_V600E_ (blue) structures during 100 ns MD simulation

**Fig. 11:**
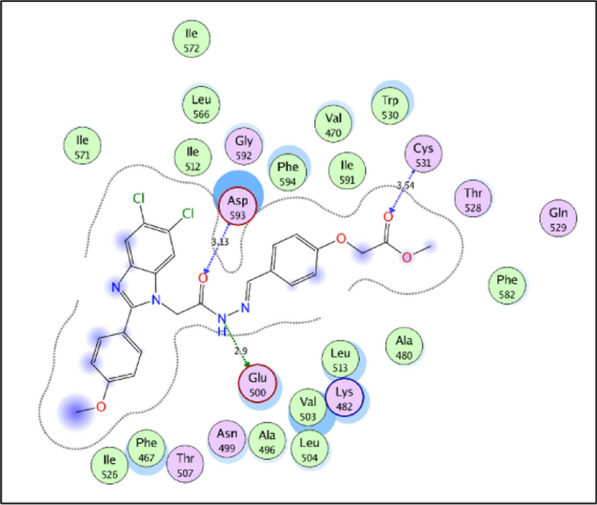
2D diagram showing the common binding pattern of compound **10h** in the kinase domain of the target kinases BRAF and BRAF_V600E_

An in-depth analysis of compound **10h** binding mode throughout the simulations in the target kinase domains showed that the target compound has a similar binding pattern in both kinases (Fig. 10). This binding pattern involves the accommodation of the central acylhydrazone moiety in the interface between the gate area and the allosteric hydrophobic back pocket interacting through hydrogen bonding interactions with the side chain carboxylate of Glu500 of the αC helix and with backbone NH of Asp593 of the conserved DFG motif in both BRAF_WT_ and BRAF_V600E_. From one side, it directs the 2-substituted-5,6-dichlorobenzimidazole towards the allosteric hydrophobic back pocket interacting with the surrounding hydrophobic side chains of Phe467, Val503, Leu504, Ile512, Leu566, Ile571, and Ile591 in both kinases through hydrophobic interactions. On the other side, this binding pattern directs the 4-substituted phenyl moiety towards the hinge region interacting through hydrogen bonding with the key amino acid Cys531 (Fig. 11).

**Fig. 12:**
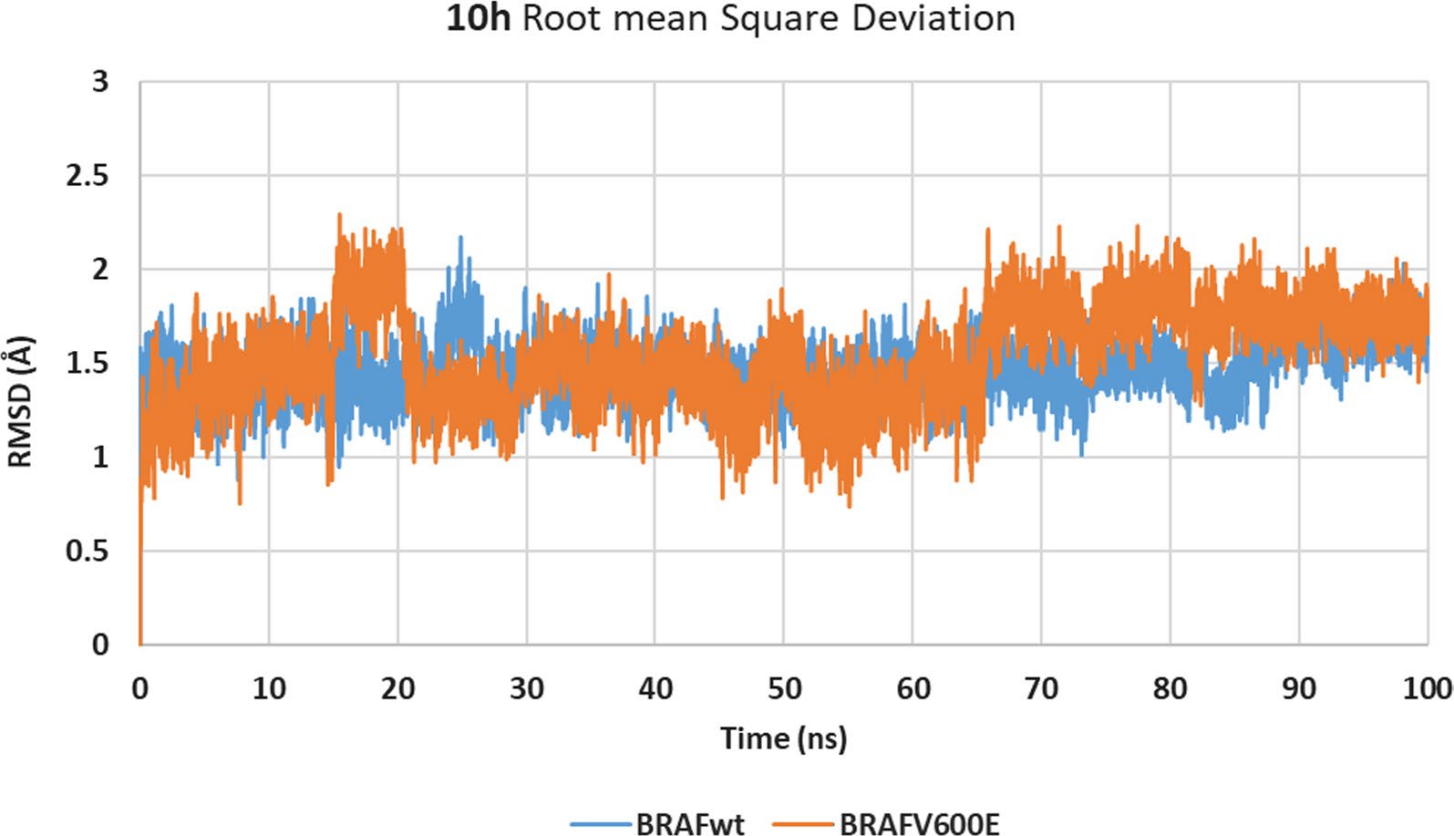
RMSD graph of compound **10h** atoms from its initial pose in BRAF (blue) and BRAF_V600E_ (orange) structures during 100 ns MD simulation

To study the dynamic behaviour of compound **10h** in the kinase domain of the target kinases, RMSD graph of the ligand atoms was plotted from its initial pose in both kinases throughout the simulation. Figure 12 indicated the pose stability of compound **10h** with average RMSD values of 1.456 and 1.540 Å, in BRAF_WT_ and BRAF_V600E_ kinase domains, respectively, from the initial poses (docking poses).

**Fig. 13:**
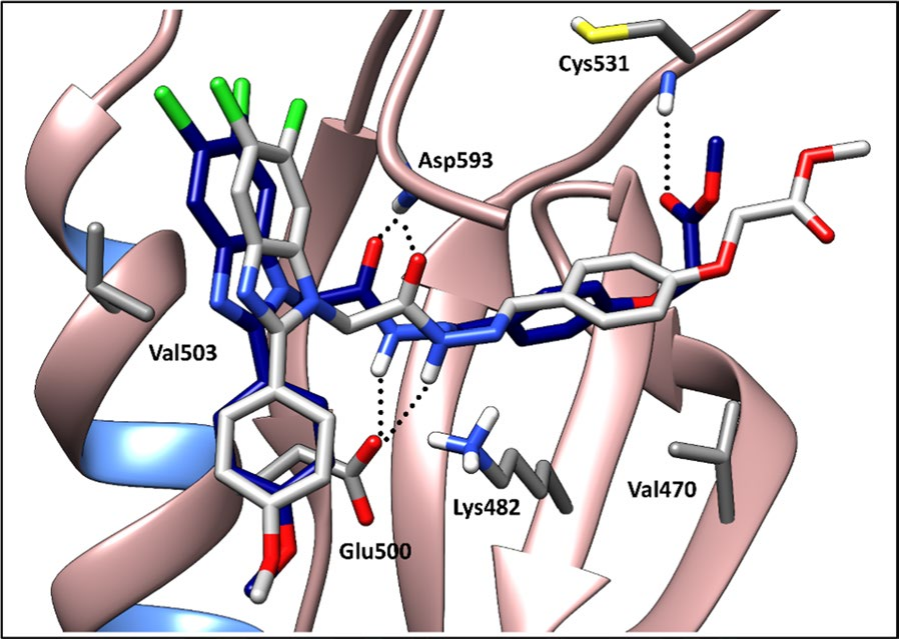
3D representation showing the dynamic behaviour of compound **10h** in the kinase domain of BRAF, in most frames (navy blue), by its proximity to Cys531, compound **10h** achieves the key hydrogen bond interaction with the hinge region, and in other frames (light grey) it moves away from Cys531

Further analysis of compound **10h** dynamic behaviour was performed using cluster analysis tool in Chimera 1.17.1. [47] in both simulations. Cluster analysis showed the stability of the 2-substituted-5,6-dichlorobenzimidazole and acylhydrazone moieties in the allosteric back pocket and the gate area, respectively, achieving the common interactions with the surrounding amino acids (vide supra). Whereas, in both simulations, the flexible methylacetate moiety showed an active dynamic behaviour throughout the simulation with proximity to Cys531, in most frames, achieving the key hydrogen bond interaction with the hinge region, and in other frames, it moves away from Cys531 (Fig. 13) which could account for **10h** slight RMSD fluctuation throughout the simulation time (Fig. 12).

The obtained poses of compound **10h** in BRAF_WT_ and BRAF_V600E_ kinase domains in the dominant clusters were scored using Molecular Mechanics/Generalized Born Surface Area (MM/GBSA) binding free energy calculation method implemented in fastDRH webserver (http://cadd.zju.edu.cn/fastdrh/) [44] and were compared to that of the co-crystalized ligand sorafenib in its experimental poses. Table 7 shows the binding free energy for compound **10h** in comparison to sorafenib in BRAF_WT_ and BRAF_V600E_ kinase domains. As can be seen, compound **10h** showed a better predicted MM/GBSA binding free energy in BRAF_WT_ kinase domain than that of sorafenib (− 60.06 vs − 58.04 kcal/mol, respectively), whereas sorafenib showed a better predicted MM/GBSA binding free energy in BRAF_V600E_ kinase domain than that of **10h** (− 59.50 vs − 55.21 kcal/mol, respectively).

**Table 7:** fastDRH predicted MM/GBSA binding free energy in kcal/mol for the most potent compound **10h** and the co-crystalized compound (Sorafenib) in BRAF_WT_ and BRAF_V600E_ active sites

Compound	Binding free energy (kcal/mol) BRAF_WT_	Binding free energy (kcal/mol) BRAF_V600E_
**10h**	− 60.06	− 55.21
Co-crystalized ligand (Sorafenib)	− 58.04	− 59.50

### Physicochemical and pharmacokinetic properties prediction

The synthesized 5,6-dichlorobenzimidazoles **10a**–**p** were submitted to SwissADME online web tool [38] to predict their physicochemical and pharmacokinetic properties. Compounds **10a**–**p** showed promising predicted properties exhibiting a predicted WlogP range of 3.30–5.50 (Table 8), high gastrointestinal absorption with no blood–brain barrier permeation (i.e., no CNS side effects).

**Table 8:** Physicochemical properties of 1-substiuted-2-(5,6-dichloro-2-(4-methoxyphenyl)-1*H*-benzo[*d*]imidazoles **10a-p**

Compound ID	MW	#Rotatable bonds	TPSA	WLOGP	Lipinski #violations	Bioavailability Score	PAINS #alerts	Synthetic Accessibility
**10a**	469.32	7	88.74	4.87	0	0.55	0	3.22
**10b**	469.32	7	88.74	4.87	0	0.55	1	3.22
**10c**	483.35	8	77.74	5.18	0	0.55	0	3.34
**10d**	483.35	8	77.74	5.18	0	0.55	0	3.34
**10e**	526.35	10	117.87	3.30	1	0.56	0	3.47
**10f**	526.35	10	117.87	3.30	1	0.56	0	3.47
**10g**	541.38	11	104.04	4.72	1	0.55	0	3.66
**10h**	541.38	11	104.04	4.72	1	0.55	0	3.65
**10i**	540.37	10	117.87	3.69	1	0.56	0	4.01
**10j**	569.44	12	104.04	5.50	1	0.55	0	4.34
**10k**	499.35	8	97.97	4.88	0	0.55	0	3.40
**10l**	499.35	8	97.97	4.88	0	0.55	1	3.40
**10m**	556.37	11	127.10	3.31	1	0.56	0	3.67
**10n**	556.37	11	127.10	3.31	1	0.56	0	3.66
**10o**	571.41	12	113.27	4.73	1	0.55	0	3.85
**10p**	571.41	12	113.27	4.73	1	0.55	0	3.84

**Fig. 14:**
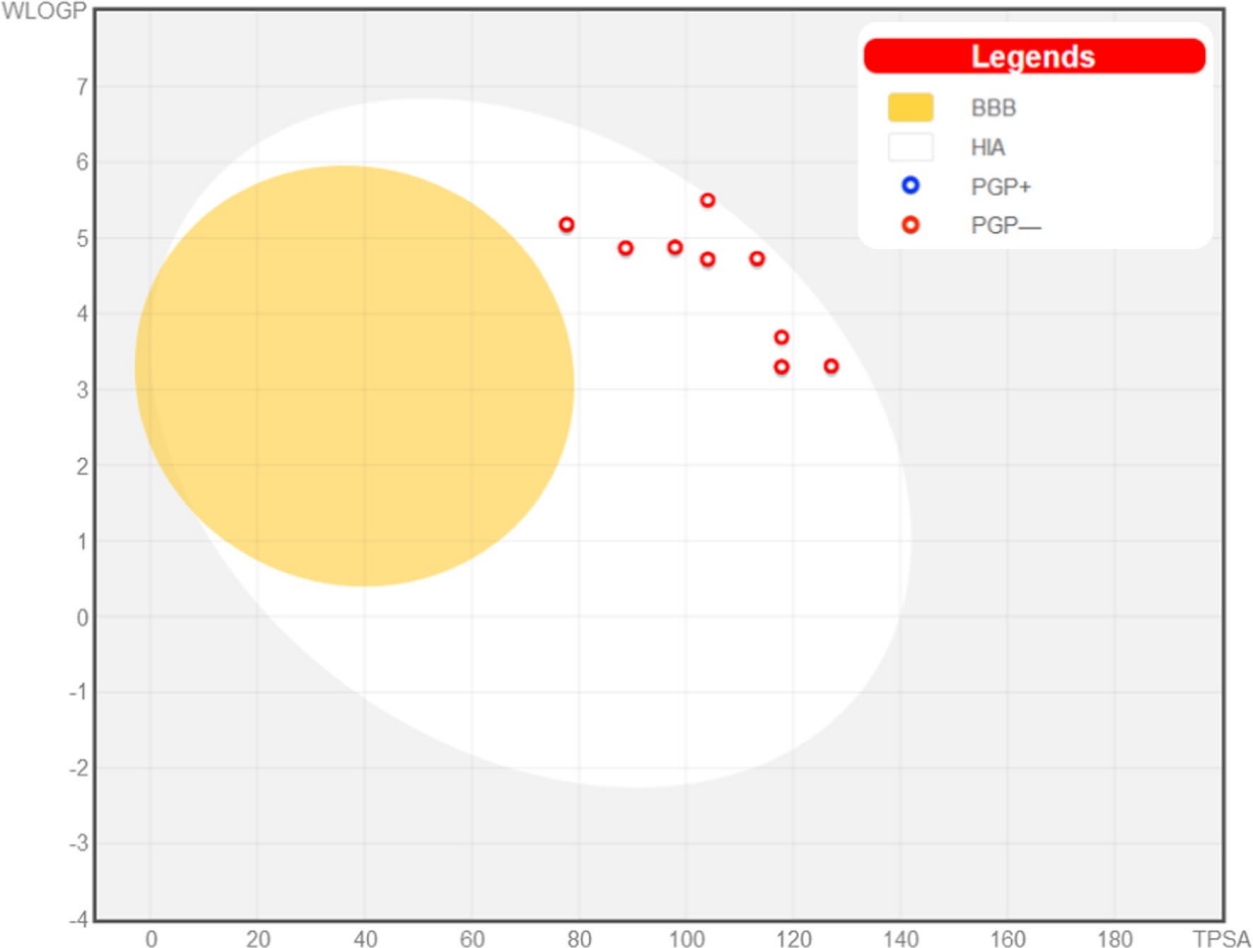
Predicted boiled-egg plot provided by SwissADME for **10a**–**p**

As for their drug-likeness, the findings displayed that the target 5,6-dichlorobenzimidazoles **10a**–**p** follow Lipinski’s rule of 5 with zero to maximum one violation [48], as some compounds have their molecular weight greater than 500. Moreover, they exhibited a promising Abbott bioavailability score of 0.55–0.56 [49] representing their promising bioavailability that was confirmed by the BOILED-Egg graph of the predicted logP *vs*. the calculated topological polar surface area (Fig. 14) [50]. They were in the human intestinal absorption (HIA) white zone with no blood–brain barrier permeation (yellow zone), furthermore, none of them was a P-glycoprotein substrate.

As for their medicinal chemistry friendliness, most of the designed and synthesized 5,6-dichlorobenzimidazole do not exhibit any of the PAIN fragments in this scaffold [51], furthermore, they showed synthetic accessibility range of 3.22–4.34, where 1 is very easy and 10 is difficult to synthesize.

These findings show that the target compounds **10a**–**p** have promising properties besides their promising antiproliferative activity.

## Conclusion

A new series of 5,6-dichlorobenzimidazole **10a**–**p** was designed as dual BRAF_WT/V600E_ inhibitors. The synthesized compounds exhibited varying degrees of inhibitory activity on BRAF_WT_ with % inhibition ranging from 21.86 to 91.20%. Among them, compound **10h**, featuring a peripheral phenyl moiety with a methyl acetate ester, emerged as the most potent derivative, achieving 91.20% inhibition. It demonstrated a potent IC_50_ value of 1.72 and 2.76 µM against BRAF_WT_ and BRAF_V600E_, respectively. Moreover, it displayed a moderate to potent GI_50_ on the tested cancer cell lines with GI_50_ reaching 1.67 µM. Further analysis of the effect of **10h** on cell cycle progression and apoptosis in HT29 colon cancer cell line proved its capability to arrest the cell cycle progression at G2/M phase and its ability to induce apoptosis in the same cell line. Molecular dynamics simulations showed that the binding pattern of compound **10h** in BRAF_WT/V600E_ kinase domains involves the accommodation of the central acylhydrazone moiety in the interface between the gate area and the allosteric hydrophobic back pocket interacting through hydrogen bonding with the key amino acids Glu500 and Asp593. From one side, it directs the 2-substituted-5,6-dichlorobenzimidazole towards the allosteric hydrophobic back pocket interacting through hydrophobic interactions with the surrounding residues. On the other side, this binding pattern directs the 4-substituted phenyl moiety towards the hinge region interacting through hydrogen bonding with the key amino acid Cys531. Analysis of the physicochemical properties of the synthesized series proved its promising drug likeness profile.

## Experimental

### Chemistry

#### General remarks

Reagents and solvents were obtained from commercial suppliers, including Acros, Aldrich, Fluka, Merck, and Sigma. These substances were used without additional purification. Solvents were also employed without the need for further purification or drying. Reaction progress was tracked through analytical thin-layer chromatography (TLC). Melting points, recorded on a Stuart SMP30 melting point apparatus, are reported without correction. ^1^H- and ^13^C-Nuclear Magnetic Resonance (NMR) spectra were obtained on Bruker instruments, with measurements at 500 (125) MHz and 400 (100) MHz, respectively, using DMSO-d_6_ as the solvent. Chemical shifts are presented in parts per million (ppm) relative to the tetramethylsilane (TMS) resonance within the specified solvent. Coupling constants are expressed in Hertz (Hz), and spectral splitting partners are denoted as follows: singlet (s), doublet (d), triplet (t), and multiplet (m). Infrared (IR) spectra (4000–400 cm⁻^1^) were acquired using a Jasco FT/IR 300 E Fourier-transform infrared spectrophotometer. HRMS was measured using a Thermo Exactive Plus Orbitrap Mass Spectrometer [Joseph Banks Laboratories—University of Lincoln—UK].

#### Synthesis of starting and target compounds

##### 6-5, 6-Dichloro-2-(4-methoxyphenyl)-1*H*-benzo[*d*]imidazole (4)

This compound was prepared according to the previously reported procedure [52]. 4-Methoxybenzaldehyde (**1**) (13.6 g, 0.1 mol) was dissolved in methanol (150 mL) and stirred for 15 min. To this, a saturated aqueous solution of Na₂S₂O₅ (18.9 g, 0.1 mol in 20 mL H₂O) was added, and the mixture was stirred at rt for 15 min. The reaction mixture was then cooled in fridge overnight, resulting in the precipitation of 4-methoxybenzaldehyde bisulfite adduct **2**, which was isolated by filtration and dried. Next, 4,5-dichloro-*o*-phenylenediamine (**3**) (1.77 g, 10 mmol) and the bisulfite adduct **2** (2.4 g, 10 mmol) were reacted in DMF (15 mL) under reflux for 2 h. The reaction mixture was then poured into ice water (100 mL), precipitating the crude product. The product was collected by filtration and purified by recrystallization from methanol to obtain compound **4** (2.35 g, 81%) as a white powder; mp 225–227 °C.

##### Methyl 2-(5,6-dichloro-2-(4-methoxyphenyl)-1*H*-benzo[*d*]imidazol-1-yl)acetate (6)

This compound was prepared according to the previously reported procedure with slight modification [52]. A solution of 5,6-dichloro-2-(4-methoxyphenyl)-1*H*-benzo[*d*]imidazole (**4**) (2.0 g, 6.82 mmol) in DMF (20 mL), (2.22 g, 6.82 mmol) of Cs_2_CO_3_ was added and the reaction mixture was stirred for 30 min followed by dropwise addition of methylbromoacetate (**5**) (0.65 mL, 6.82 mmol). The reaction mixture was the stirred at room temperature for 12 h. The reaction mixture was poured onto cold water and the precipitate was filtered, dried and recrystallized from methanol to afford compound** 6** (2.10 g, 85%) as a white powder; mp 210–212 °C.

##### 2-(5,6-Dichloro-2-(4-methoxyphenyl)-1*H*-benzo[*d*]imidazol-1-yl)acetohydrazide (8)

This compound was prepared according to the previously reported procedure with slight modification [52]. Methyl 2-(5,6-dichloro-2-(4-methoxyphenyl)-1*H*-benzo[*d*]imidazol-1-yl)acetate (**6**) (1.5 g, 4.11 mmol) was dissolved in 20 mL of ethanol. Hydrazine hydrate (98%) (0.85 mL, 16.43 mmoL) was added slowly, and the mixture was refluxed at 90 °C for 2 h. Once the reaction was complete, the reaction mixture was cooled, and the solvent was removed under vacuum. The solid that formed was collected by filtration, rinsed with cold ethanol, and dried to give compound **8** (1.24 g, 83%) as a buff powder; mp 268–270 °C.

##### General procedure for the synthesis of benzimidazole derivatives 10a-p

To a solution of 5,6-dicholorbenzimidazole acid hydrazide **8** (100 mg, 0.27 mmol, 2 eq) and the appropriate aldehyde (0.81 mmol, 6 eq) in 5 mL absolute ethanol, (8 µL, 0.14 mmol, 1 eq) of glacial acetic acid was added dropwise and the reaction was stirred at rt for 10 h. Upon completion of the reaction, 20 mL of distilled water was added and the formed precipitate was filtered and recrystallized from ethanol to obtain the desired product.

##### 2-(5,6-Dichloro-2-(4-methoxyphenyl)-1*H*-benzo[*d*]imidazol-1-yl)-*N*'-(3-hydroxybenzylidene)acetohydrazide (10a)

A white precipitate was obtained in a yield of 72%; mp 168–170 °C; IR (KBr) υ_max_ 3406 and 3213 (NH), 3055 (CH aromatic), 2974 and 2940 (CH aliphatic), 1701 (C=O), 1609, 1578, 1458 cm^−1^; ^1^H NMR (400 MHz; DMSO-*d*_6_) major conformer *δ*_H_ 3.80 (s, 3H), 5.51 (s, 2H), 6.83 (dd, ^3^*J* = 8.8 Hz, ^4^*J* = 1.6 Hz, 1H), 7.09–7.11 (m, 3H), 7.12–7.16 (m, 1H), 7.23 (t, ^3^*J* = 8.0 Hz, 1H), 7.64 (d, ^3^*J* = 8.8 Hz, 2H), 7.95 (s, 2H), 8.06 (s, 1H), 9.60 (s, 1H), 11.74 ppm (s, 1H); ^1^H NMR (400 MHz; DMSO-*d*_6_) minor conformer *δ*_H_ 3.83 (s, 3H), 5.07 (s, 2H), 6.83 (ov. dd, ^3^*J* = 8.8 Hz, ^4^*J* = 1.6 Hz, 1H), 7.09–7.11 (ov. m, 3H), 7.12–7.16 (ov. m, 1H), 7.24 (t, ^3^*J* = 7.6 Hz, 1H), 7.70 (d, ^3^*J* = 8.4 Hz, 2H), 7.97 (s, 1H), 7.98 (s, 1H), 8.13 (s, 1H), 9.63 (s, 1H), 11.81 ppm (s, 1H); ^13^C NMR (100 MHz; DMSO-*d*_6_) major conformer *δ*_C_ 46.28, 55.34, 112.86, 113.03, 114.39, 117.43, 118.48, 119.87, 121.48, 124.48, 124.72, 129.87, 130.45, 135.10, 136.54, 142.00, 144.78, 155.96, 157.64, 160.76, 168.02 ppm; ^13^C NMR (100 MHz; DMSO-*d*_6_) minor conformer *δ*_C_ 46.58, 55.41, 112.55, 112.70, 114.42, 117.71, 118.99, 120.02, 121.28, 124.65, 124.79, 129.93, 130.67, 135.16, 136.32, 141.97, 147.91, 155.91, 157.68, 160.85, 163.22 ppm; HRMS (-) ESI *m/z* Calculated for C_23_H_17_Cl_2_N_4_O_3_ [M-H]^−^: 467.0678, Found: 467.0695.

##### 2-(5,6-Dichloro-2-(4-methoxyphenyl)-1*H*-benzo[*d*]imidazol-1-yl)-*N*'-(4-hydroxybenzylidene)acetohydrazide (10b)

A white precipitate was obtained in a yield of 67%; mp 163–165 °C; IR (KBr) υ_max_ 3183 (NH), 3063 (CH aromatic), 2959, 2932 and 2835 (CH aliphatic), 1678 (C=O), 1605, 1578, 1520, 1481 cm^−1^; ^1^H NMR (400 MHz; DMSO-*d*_6_) major conformer *δ*_H_ 3.80 (s, 3H), 5.49 (s, 2H), 6.80 (d, ^3^*J* = 8.4 Hz, 2H), 7.09 (d, ^3^*J* = 8.8 Hz, 2H), 7.53 (d, ^3^* J* = 7.2 Hz, 2H), 7.65 (d, ^3^*J* = 8.8 Hz, 2H), 7.94 (d, ^3^*J* = 6.5 Hz, 2H), 8.04 (s, 1H), 9.93 (s, 1H), 11.60 ppm (s, 1H); ^1^H NMR (400 MHz; DMSO-*d*_6_) minor conformer *δ*_H_ 3.83 (s, 3H), 5.04 (s, 2H), 6.82 (ov. d, ^3^*J* = 8.0 Hz, 2H), 7.13 (d, ^3^*J* = 8.4 Hz, 2H), 7.54 (d, ^3^*J* = 8.4 Hz, 2H), 7.70 (d, ^3^*J* = 8.4 Hz, 2H), 7.95 (ov. d, ^3^*J* = 6.5 Hz, 2H), 8.12 (s, 1H), 9.96 (s, 1H), 11.66 ppm (s, 1H); ^13^C NMR (100 MHz; DMSO-*d*_6_) major conformer *δ*_C_ 46.33, 55.37, 112.84, 114.39, 115.70, 119.88, 121.53, 124.49, 124.73, 124.90, 128.86, 130.50, 136.56, 142.01, 144.87, 156.01, 159.46, 160.77, 167.73 ppm; ^13^C NMR (100 MHz; DMSO-*d*_6_) minor conformer *δ*_C_ 46.57, 55.43, 112.54, 114.43, 115.77, 120.02, 121.31, 124.66, 124.80, 124.87, 129.07, 130.71, 136.32, 141.99, 148.17, 155.96, 159.66, 160.87, 162.86 ppm; HRMS (−) ESI *m/z* Calculated for C_23_H_17_Cl_2_N_4_O_3_ [M−H]^−^: 467.0678, Found: 467.0736.

##### 2-(5,6-Dichloro-2-(4-methoxyphenyl)-1*H*-benzo[*d*]imidazol-1-yl)-*N*'-(3-methoxybenzylidene)acetohydrazide (10c)

A buff precipitate was obtained in a yield of 83%; mp 255–257 °C; IR (KBr) υ_max_ 3213 (NH), 3059 (CH aromatic), 2905 and 2835 (CH aliphatic), 1670 (C=O), 1608, 1512, 1485, 1458 cm^−1^; ^1^H NMR (400 MHz; DMSO-*d*_6_) major conformer *δ*_H_ 3.79 (s, 6H), 5.51 (s, 2H), 6.99 (d, ^3^*J* = 8.8 Hz, 2H), 7.09 (d, ^3^*J* = 8.4 Hz, 2H), 7.65 (dd, ^3^*J* = 8.8 Hz, ^4^*J* = 2.8 Hz, 4H), 7.95 (s, 1H), 7.98 (s, 1H), 8.05 (s, 1H), 11.67 ppm (s, 1H); ^1^H NMR (400 MHz; DMSO-*d*_6_) minor conformer *δ*_H_ 3.78 (s, 3H), 3.83 (s, 3H), 5.05 (s, 2H), 7.01 (ov. d, ^3^*J* = 8.8 Hz, 2H), 7.13 (d, ^3^*J* = 8.8 Hz, 2H), 7.65 (ov. dd, ^3^*J* = 8.8 Hz, ^4^*J* = 2.8 Hz, 2H), 7.70 (d, ^3^*J* = 8.8 Hz, 2H), 7.96 (s, 1H), 7.97 (s, 1H), 8.17 (s, 1H), 11.73 ppm (s, 1H); ^13^C NMR (100 MHz; DMSO-*d*_6_) major conformer *δ*_C_ 46.35, 55.37, 112.85, 114.32, 114.40, 119.90, 121.53, 124.52, 124.76, 126.46, 128.74, 130.52, 136.56, 142.01, 144.51, 156.03, 160.79, 160.91, 167.87 ppm; ^13^C NMR (100 MHz; DMSO-*d*_6_) minor conformer *δ*_C_ 46.61, 55.25, 55.45, 111.79, 112.56, 114.45, 120.05, 121.31, 124.70, 124.83, 128.93, 130.73, 136.32, 144.48, 147.80, 155.98, 160.75, 161.08, 163.03, 168.23 ppm; HRMS (−) ESI *m/z* Calculated for C_24_H_19_Cl_2_N_4_O_3_ [M-H]^−^: 481.0834, Found: 481.0852.

##### 2-(5,6-Dichloro-2-(4-methoxyphenyl)-1*H*-benzo[*d*]imidazol-1-yl)-*N*'-(4-methoxybenzylidene)acetohydrazide (10d)

A white precipitate was obtained in a yield of 70%; mp 265–267 °C; IR (KBr) υ_max_ 3406 and 3175 (NH), 3059 and 3017 (CH aromatic), 2958 and 2835 (CH aliphatic), 1670 (C=O), 1612, 1512, 1485, 1454 cm^−1^; ^1^H NMR (400 MHz; DMSO-*d*_6_) major conformer *δ*_H_ 3.80 (s, 6H), 5.51 (s, 2H), 6.99 (d, ^3^*J* = 8.8 Hz, 2H), 7.09 (d, ^3^*J* = 8.8 Hz, 2H), 7.65 (d, ^3^*J* = 8.8 Hz, 2H), 7.66 (d, ^3^*J* = 8.8 Hz, 2H), 7.95 (s, 1H), 7.98 (s, 1H), 8.05 (s, 1H), 11.67 ppm (s, 1H); ^1^H NMR (400 MHz; DMSO-*d*_6_) minor conformer *δ*_H_ 3.80 (ov. s, 3H), 3.83 (s, 3H), 5.05 (s, 2H), 7.01 (ov. d, ^3^*J* = 8.8 Hz, 2H), 7.13 (d, ^*3*^*J* = 8.8 Hz, 2H), 7.65 (ov. d, ^3^*J* = 8.8 Hz, 2H), 7.70 (d, ^3^*J* = 8.8 Hz, 2H), 7.96 (s, 1H), 7.97 (s, 1H), 8.17 (s, 1H), 11.73 ppm (s, 1H); ^13^C NMR (100 MHz; DMSO-*d*_6_) major conformer *δ*_C_ 46.30, 55.32, 112.80, 114.26, 114.34, 119.85, 121.52, 124.44, 124.68, 126.42, 128.67, 130.46, 136.53, 141.99, 144.40, 155.96, 160.72, 160.84, 167.82 ppm; ^13^C NMR (100 MHz; DMSO-*d*_6_) minor conformer *δ*_C_ 46.56, 55.39, 112.51, 114.34, 114.39, 120.00, 121.29, 124.61, 124.75, 128.85, 130.67, 136.30, 147.70, 155.91, 160.83, 161.02, 162.96 ppm; HRMS (−) ESI *m/z* Calculated for C_24_H_19_Cl_2_N_4_O_3_ [M−H]^−^: 481.0834, Found: 481.0853.

##### 2-(3-((2-(2-(5,6-Dichloro-2-(4-methoxyphenyl)-1*H*-benzo[*d*]imidazol-1-yl)acetyl)hydrazono)methyl)phenoxy)acetic acid (10e)

A white precipitate was obtained in a yield of 68%; mp 153–155 °C; IR (KBr) υ_max_ 3341, 3210 and 3144 (NH), 3082 (CH aromatic), 2940, 2909 and 2839 (CH aliphatic), 1744 and 1667 (C=O), 1609, 1578, 1481 cm^−1^; ^1^H NMR (400 MHz; DMSO-*d*_6_) major conformer *δ*_H_ 3.79 (s, 3H), 4.71 (s, 2H), 5.55 (s, 2H), 6.98 (d, ^3^*J* = 7.2 Hz, 1H), 7.09 (d, ^3^*J* = 8.4 Hz, 2H), 7.28 (s, 1H), 7.30 (s, 1H), 7.34 (d, ^3^*J* = 7.6 Hz, 1H), 7.66 (d, ^3^*J* = 8.8 Hz, 2H), 7.95 (s, 1H), 8.00 (s, 1H), 8.06 (s, 1H), 11.81 ppm (s, 1H); ^1^H NMR (400 MHz; DMSO-*d*_6_) minor conformer *δ*_H_ 3.83 (s, 3H), 4.71 (ov. s, 2H), 5.07 (s, 2H), 6.98 (ov. d, ^3^*J* = 7.2 Hz, 1H), 7.14 (d, ^3^*J* = 8.8 Hz, 2H), 7.23 (s, 1H), 7.28 (ov. s, 1H), 7.37 (d, ^3^*J* = 7.2 Hz, 1H), 7.70 (d, ^3^*J* = 8.8 Hz, 2H), 7.97 (s, 1H), 7.98 (s, 1H), 8.20 (s, 1H), 11.89 (s, 1H); ^13^C NMR (100 MHz; DMSO-*d*_6_) major conformer *δ*_C_ 46.36, 55.38, 64.65, 112.59, 112.85, 114.43, 116.36, 119.92, 120.28, 121.52, 124.56, 124.80, 130.00, 130.53, 135.32, 136.57, 142.02, 144.31, 156.07, 158.11, 160.80, 168.23, 170.19 ppm; ^13^C NMR (100 MHz; DMSO-*d*_6_) minor conformer *δ*_C_ 46.64, 55.46, 64.72, 112.27, 113.65, 114.48, 116.93, 120.06, 120.54, 121.29, 121.45, 123.03, 124.73, 124.88, 130.06, 130.73, 136.34, 147.70, 155.98, 158.15, 160.91, 163.37, 170.15 ppm; HRMS (−) ESI *m/z* Calculated for C_25_H_19_Cl_2_N_4_O_5_ [M-H]^−^: 525.0733, Found: 525.0793.

##### 2-(4-((2-(2-(5,6-Dichloro-2-(4-methoxyphenyl)-1*H*-benzo[*d*]imidazol-1-yl)acetyl)hydrazono)methyl)phenoxy)acetic acid (10f)

A buff precipitate was obtained in a yield of 74%; mp 163–165 °C; IR (KBr) υ_max_ 3213 and 3136 (NH), 2920 and 2839 (CH aliphatic), 1736 and 1663 (C=O), 1605, 1478, 1458 cm^−1^; ^1^H NMR (500 MHz; DMSO-*d*_6_) major conformer *δ*_H_ 3.79 (s, 3H), 4.74 (s, 2H), 5.52 (s, 2H), 6.97 (d, ^3^*J* = 8.5 Hz, 2H), 7.09 (d, ^3^*J* = 8.5 Hz, ^4^*J* = 1.5 Hz, 2H), 7.64–7.67 (m, 4H), 7.95 (s, 1H), 7.98 (s, 1H), 8.05 (s, 1H), 11.69 (s, 1H), 13.05 (br., 1H); ^1^H NMR (500 MHz; DMSO-*d*_6_) minor conformer *δ*_H_ 3.83 (s, 3H), 4.74 (ov. s, 2H), 5.06 (s, 2H),), 6.99 (d, ^3^*J* = 8.5 Hz, 2H), 7.13 (dd, ^3^*J* = 8.5 Hz, ^4^*J* = 1.5 Hz, 2H), 7.64–7.66 (ov. m, 2H), 7.70 (dd, ^3^*J* = 8.0 Hz, ^4^*J* = 1.0 Hz, 2H), 7.96 (s, 2H), 8.18 (s, 1H), 11.76 (s, 1H), 13.05 (ov. br., 1H); ^13^C NMR (125 MHz; DMSO-*d*_6_) major conformer *δ*_C_ 46.27, 55.33, 64.57, 112.78, 114.35, 114.80, 119.95, 124.47, 124.70, 126.93, 128.60, 130.46, 136.52, 141.99, 144.30, 155.99, 159.32, 160.75, 167.83, 169.91 ppm; ^13^C NMR (125 MHz; DMSO-*d*_6_) minor conformer *δ*_C_ 46.26, 55.39, 64.53, 112.49, 114.40, 114.88, 124.48, 124.75, 126.92, 128.78, 130.67, 136.55, 141.97, 144.31, 156.01, 159.34, 160.77, 167.86, 169.95 ppm; HRMS (−) ESI *m/z* Calculated for C_25_H_19_Cl_2_N_4_O_5_ [M-H]^−^: 525.0733, Found: 525.0748.

##### Methyl-2-(3-((2-(2-(5,6-dichloro-2-(4-methoxyphenyl)-1*H*-benzo[*d*]imidazol-1-yl)acetyl)hydrazono) methyl)phenoxy)acetate (10g)

A white precipitate was obtained in a yield of 81%; mp 189–191 °C; IR (KBr) υ_max_ 3252 and 3198 (NH), 3071, 3036 and 3005 (CH aromatic), 2940 (CH aliphatic), 1751, 1721 and 1690 (C=O), 1609, 1582, 1481 cm^−1^; ^1^H NMR (400 MHz; DMSO-*d*_6_) major conformer *δ*_H_ 3.67 (s, 3H), 3.79 (s, 3H), 4.84 (s, 2H), 5.55 (s, 2H), 6.99–7.02 (m, 1H), 7.09 (d, ^3^*J* = 8.8 Hz, 2H), 7.25–7.39 (m, 3H), 7.66 (d, ^3^*J* = 8.8 Hz, 2H), 7.96 (s, 1H), 8.00 (s, 1H), 8.06 (s, 1H), 11.83 ppm (s, 1H); ^1^H NMR (400 MHz; DMSO-*d*_6_) minor conformer *δ*_H_ 3.70 (s, 3H), 3.83 (s, 3H), 4.84 (ov. s, 2H), 5.07 (s, 2H), 6.99–7.02 (ov. m, 1H), 7.14 (d, ^3^*J* = 8.8 Hz, 2H), 7.25–7.39 (ov. m, 3H), 7.70 (d, ^3^*J* = 8.8 Hz, 2H), 7.97 (s, 1H), 7.98 (s, 1H), 8.20 (s, 1H), 11.88 ppm (s, 1H); ^13^C NMR (100 MHz; DMSO-*d*_6_) major conformer *δ*_C_ 46.30, 51.76, 55.30, 64.57, 112.53, 112.77, 114.33, 116.26, 119.85, 120.41, 121.50, 124.43, 124.66, 129.94, 130.42, 135.32, 136.52, 141.99, 144.05, 155.94, 157.84, 160.71, 168.17, 169.08 ppm; ^13^C NMR (100 MHz; DMSO-*d*_6_) minor conformer *δ*_C_ 46.59, 51.82, 55.37, 64.57, 112.53, 112.69, 114.38, 116.62, 120.00, 121.27, 124.59, 124.73, 130.00, 130.63, 135.37, 136.30, 141.96, 147.48, 155.87, 157.88, 160.80, 163.27, 169.02 ppm; HRMS (−) ESI *m/z* Calculated for C_26_H_21_Cl_2_N_4_O_5_ [M−H]^−^: 539.0889, Found: 539.0944.

##### Methyl-2-(4-((2-(2-(5,6-dichloro-2-(4-methoxyphenyl)-1*H*-benzo[*d*]imidazol-1-yl)acetyl)hydrazono)methyl)phenoxy)acetate (10h)

A white precipitate was obtained in a yield of 76%; mp 195–197 °C; IR (KBr) υ_max_ 3175 (NH), 3059 (CH aromatic), 2974, 2947 and 2843 (CH aliphatic), 1771 and 1674 (C=O), 1609, 1512, 1489 cm^−1^; ^1^H NMR (400 MHz; DMSO-*d*_6_) major conformer *δ*_H_ 3.70 (s, 3H), 3.79 (s, 3H), 4.86 (s, 2H), 5.52 (s, 2H), 6.99 (d, ^3^*J* = 8.8 Hz, 2H), 7.09 (d, ^3^*J* = 8.8 Hz, 2H), 7.64 (d, ^3^*J* = 8.8 Hz, 2H), 7.65 (d, ^3^*J* = 8.8 Hz, 2H), 7.95 (s, 1H), 7.98 (s, 1H), 8.05 (s, 1H), 11.69 ppm (s, 1H); ^1^H NMR (400 MHz; DMSO-*d*_6_) minor conformer *δ*_H_ 3.70 (ov. s, 3H), 3.83 (s, 3H), 4.86 (ov. s, 2H), 5.05 (s, 2H), 7.01 (ov. d, ^3^*J* = 8.4 Hz, 2H), 7.13 (d, ^3^*J* = 8.4 Hz, 2H), 7.65 (ov. d, ^3^*J* = 8.8 Hz, 2H), 7.70 (d, ^3^*J* = 8.8 Hz, 2H), 7.96 (s, 2H), 8.17 (s, 1H), 11.75 ppm (s, 1H); ^13^C NMR (100 MHz; DMSO-*d*_6_) major conformer *δ*_C_ 46.34, 51.92, 55.36, 64.62, 112.83, 114.38, 114.87, 119.89, 121.52, 124.50, 124.74, 127.18, 128.67, 130.50, 136.56, 142.01, 144.24, 156.02, 159.13, 160.77, 167.92, 169.04 ppm; ^13^C NMR (100 MHz; DMSO-*d*_6_) minor conformer *δ*_C_ 46.59, 51.92, 55.43, 64.62, 112.55, 114.43, 114.96, 120.03, 121.30, 124.68, 124.81, 127.18, 128.86, 130.71, 136.32, 142.01, 147.58, 155.96, 159.31, 160.87, 163.07, 169.04 ppm; HRMS (−) ESI *m/z* Calculated for C_26_H_21_Cl_2_N_4_O_5_ [M−H]^−^: 539.0889, Found: 539.0927.

##### 3-(4-((2-(2-(5,6-Dichloro-2-(4-methoxyphenyl)-1*H*-benzo[*d*]imidazol-1-yl)acetyl)hydrazono)methyl)phenoxy)propanoic acid (10i)

A yellowish-white precipitate was obtained in a yield of 83%; mp 164–166 °C; IR (KBr) υ_max_ 3190 (NH), 3093 (CH aromatic), 2963, 2936 and 2843 (CH aliphatic), 1690 (C=O), 1609, 1458 cm^−1^; ^1^H NMR (400 MHz; DMSO-*d*_6_) major conformer *δ*_H_ 1.51 (d, ^3^*J* = 6.8 Hz, 3H), 3.79 (s, 3H), 4.91 (q, ^3^*J* = 6.8 Hz, 1H), 5.52 (s, 2H), 6.92 (d, ^3^*J* = 8.8 Hz, 2H), 7.09 (d, ^3^*J* = 8.8 Hz, 2H), 7.63 (d, ^3^*J* = 8.4 Hz, 2H), 7.65 (d, ^3^*J* = 8.8 Hz, 2H), 7.95 (s, 1H), 7.96 (s, 1H), 8.04 (s, 1H), 11.68 ppm (s, 1H); ^1^H NMR (400 MHz; DMSO-*d*_6_) minor conformer *δ*_H_ 1.51 (ov. d, ^3^*J* = 6.8 Hz, 3H), 3.83 (s, 3H), 4.91 (ov. q, ^3^*J* = 6.8 Hz, 1H), 5.05 (s, 2H), 6.94 (ov. d, ^3^*J* = 8.4 Hz, 2H), 7.13 (d, ^3^*J* = 8.8 Hz, 2H), 7.63 (ov. d, ^3^*J* = 8.4 Hz, 2H), 7.70 (d, ^3^*J* = 8.8 Hz, 2H), 7.96 (s, 2H), 8.16 (s, 1H), 11.74 ppm (s, 1H); ^13^C NMR (100 MHz; DMSO-*d*_6_) major conformer *δ*_C_ 18.19, 46.30, 55.32, 71.54, 112.80, 114.34, 115.03, 119.85, 121.52, 124.44, 124.68, 126.83, 128.62, 130.46, 136.54, 141.99, 144.20, 155.97, 159.02, 160.73, 167.86, 172.83 ppm; ^13^C NMR (100 MHz; DMSO-*d*_6_) minor conformer *δ*_C_ 18.19, 46.57, 55.39, 71.54, 112.52, 114.39, 115.10, 120.00, 121.29, 124.61, 124.75, 126.83, 128.80, 130.67, 136.30, 141.97, 147.56, 155.91, 159.19, 160.82, 162.99, 172.83 ppm; HRMS (−) ESI *m/z* Calculated for C_26_H_21_Cl_2_N_4_O_5_ [M−H]^−^: 539.0889, Found: 539.0939.

##### Ethyl-2-(4-((2-(2-(5,6-dichloro-2-(4-methoxyphenyl)-1*H*-benzo[*d*]imidazol-1-yl)acetyl) hydrazono)methyl)phenoxy)propanoate (10j)

A white precipitate was obtained in a yield of 71%; mp 200–202 °C; IR (KBr) υ_max_ 3202 and 3117 (NH), 3093 and 3052 (CH aromatic), 2982, 2943 and 2839 (CH aliphatic), 1751 and 1701 (C=O), 1609, 1512, 1454 cm^−1^; ^1^H NMR (400 MHz; DMSO-*d*_6_) major conformer *δ*_H_ 1.17 (t, ^3^*J* = 6.8 Hz, 3H), 1.52 (d, ^3^*J* = 6.8 Hz, 3H), 3.79 (s, 3H), 4.15 (q, ^3^*J* = 6.8 Hz, 2H), 5.03 (q, ^3^*J* = 6.8 Hz, 1H), 5.51 (s, 2H), 6.93 (d, ^3^*J* = 8.8 Hz, 2H), 7.09 (d, ^3^*J* = 8.8 Hz, 2H), 7.63–7.66 (m, 4H), 7.95 (s, 1H), 7.97 (s, 1H), 8.04 (s, 1H), 11.68 ppm (s, 1H); ^1^H NMR (400 MHz; DMSO-*d*_6_) minor conformer *δ*_H_ 1.17 (ov. t, ^3^*J* = 6.8 Hz, 3H), 1.52 (ov. d, ^3^*J* = 6.8 Hz, 3H), 3.83 (s, 3H), 4.15 (ov. q, ^3^*J* = 7.2 Hz, 2H), 5.01–5.05 (ov. m, 3H), 6.97 (d, ^3^*J* = 8.8 Hz, 2H), 7.13 (d, ^3^*J* = 8.8 Hz, 2H), 7.63 – 7.66 (ov. m, 2H), 7.70 (d, ^3^*J* = 8.8 Hz, 2H), 7.97 (ov. s, 2H), 8.16 (s, 1H), 11.75 ppm (s, 1H); ^13^C NMR (100 MHz; DMSO-*d*_6_) major conformer *δ*_C_ 13.98, 18.14, 46.31, 55.33, 60.89, 71.65, 112.80, 114.34, 115.13, 119.86, 121.52, 124.44, 124.68, 127.08, 128.65, 130.46, 136.54, 142.00, 144.13, 155.98, 158.76,, 160.73, 167.87, 171.23 ppm; ^13^C NMR (100 MHz; DMSO-*d*_6_) minor conformer *δ*_C_ 13.98, 18.40, 46.57, 55.39, 60.89, 71.65, 112.52, 114.40, 115.21, 120.00, 121.29, 124.61, 124.75, 127.08, 128.83, 130.67, 136.30, 141.97, 147.49, 155.91, 158.94, 160.83, 163.01, 171.23 ppm; HRMS (−) ESI *m/z* Calculated for C_28_H_25_Cl_2_N_4_O_5_ [M−H]^−^: 567.1202, Found: 567.1222.

##### 2-(5,6-Dichloro-2-(4-methoxyphenyl)-1*H*-benzo[*d*]imidazol-1-yl)-*N*'-(3-hydroxy-4-methoxy benzylidene)acetohydrazide (10k)

A buff precipitate was obtained in a yield of 69%; mp 156–158 °C; IR (KBr) υ_max_ 3202 (NH), 3044 and 3009 (CH aromatic), 2967 and 2936 (CH aliphatic), 1694 and 1659 (C=O), 1605, 1516, 1458 cm^−1^; ^1^H NMR (400 MHz; DMSO-*d*_6_) major conformer *δ*_H_ 3.78 (s, 3H), 3.79 (s, 3H), 5.51 (s, 2H), 6.81 (d, ^3^*J* = 8.0 Hz, 1H), 7.09 (d, ^3^*J* = 8.8 Hz, 3H), 7.28 (d, ^4^*J* = 1.6 Hz, 1H), 7.66 (d, ^3^*J* = 8.8 Hz, 2H), 7.92 (s, 1H), 7.95 (s, 1H), 8.04 (s, 1H), 9.55 (s, 1H), 11.64 ppm (s, 1H); ^1^H NMR (400 MHz; DMSO-*d*_6_) minor conformer *δ*_H_ 3.79 (ov. s, 3H), 3.83 (s, 3H), 5.05 (s, 2H), 6.83 (d, ^3^*J* = 8.0 Hz, 1H), 7.13 (d, ^3^*J* = 8.8 Hz, 3H), 7.28 (ov. d, ^4^*J* = 1.6 Hz, 1H), 7.70 (d, ^3^*J* = 8.8 Hz, 2H), 7.95 (s, 1H), 7.96 (s, 1H), 8.10 (s, 1H), 9.77 (s, 1H), 11.68 ppm (s, 1H); ^13^C NMR (100 MHz; DMSO-*d*_6_) major conformer *δ*_C_ 46.38, 55.36, 55.66, 109.73, 112.81, 114.40, 115.53, 119.90, 121.54, 121.67, 124.52, 124.74, 125.32, 130.52, 136.54, 142.01, 145.03, 148.00, 149.00, 156.00, 160.79, 167.85 ppm; ^13^C NMR (100 MHz; DMSO-*d*_6_) minor conformer *δ*_C_ 46.58, 55.44, 55.58, 109.18, 112.54, 114.43, 115.48, 120.03, 121.32, 122.33, 124.67, 124.80, 125.29, 128.74, 130.72, 136.32, 148.07, 148.37, 149.23, 155.98, 160.88, 162.90 ppm; HRMS (−) ESI *m/z* Calculated for C_24_H_19_Cl_2_N_4_O_4_ [M−H]^−^: 497.0783, Found: 497.0844.

##### 2-(5,6-Dichloro-2-(4-methoxyphenyl)-1*H*-benzo[*d*]imidazol-1-yl)-*N*'-(4-hydroxy-3-methoxy benzylidene)acetohydrazide (10l)

A white precipitate was obtained in a yield of 78%; mp 151–153 °C; IR (KBr) υ_max_ 3190 (NH), 3086 (CH aromatic), 2970 (CH aliphatic), 1686 (C=O), 1609, 1577, 1516, 1458 cm^−1^; ^1^H NMR (400 MHz; DMSO-*d*_6_) major conformer *δ*_H_ 3.80 (s, 6H), 5.49 (s, 2H), 6.96 (d, ^3^*J* = 8.4 Hz, 1H), 7.05 (dd, ^3^*J* = 8.0 Hz, ^4^*J* = 1.6 Hz, 1H), 7.10 (d, ^3^*J* = 8.8 Hz, 2H), 7.19 (d, ^4^*J* = 1.6 Hz, 1H), 7.64 (d, ^3^*J* = 8.4 Hz, 2H), 7.90 (s, 1H), 7.95 (s, 1H), 8.05 (s, 1H), 9.18 (s, 1H), 11.63 ppm (s, 1H); ^1^H NMR (400 MHz; DMSO-*d*_6_) minor conformer 3.80 (ov. s, 3H), 3.83 (s, 3H), 5.04 (s, 2H), 6.97 (d, ^3^*J* = 8.0 Hz, 1H), 7.05 (ov. dd, ^3^*J* = 8.0 Hz, ^4^*J* = 1.6 Hz, 1H), 7.14 (d, ^3^*J* = 8.8 Hz, 2H), 7.22 (d, ^4^*J* = 1.6 Hz, 1H), 7.70 (d, ^3^*J* = 8.8 Hz, 2H), 7.96 (s, 1H), 7.97 (s, 1H), 8.07 (s, 1H), 9.30 (s, 1H), 11.68 ppm (s, 1H); ^13^C NMR (100 MHz; DMSO-*d*_6_) major conformer *δ*_C_ 46.25, 55.35, 55.64, 111.80, 112.49, 112.87, 114.39, 119.86, 120.20, 121.49, 124.56, 124.70, 126.70, 130.46, 136.55, 141.99, 144.86, 146.76, 149.80, 155.95, 160.76, 167.73 ppm; ^13^C NMR (100 MHz; DMSO-*d*_6_) minor conformer *δ*_C_ 46.56, 55.41, 55.59, 111.85, 112.32, 112.54, 114.42, 120.01, 120.20, 121.30, 124.63, 124.76, 126.70, 130.68, 136.31, 141.99, 146.88, 147.97, 150.01, 155.92, 160.84, 162.91 ppm; HRMS (−) ESI *m/z* Calculated for C_24_H_19_Cl_2_N_4_O_4_ [M−H]^−^: 497.0783, Found: 497.0805.

##### 2-(4-((2-(2-(5,6-Dichloro-2-(4-methoxyphenyl)-1*H*-benzo[*d*]imidazol-1-yl)acetyl)hydrazono)methyl) -2-methoxyphenoxy)acetic acid (10m)

A yellowish-white precipitate was obtained in a yield of 72%; mp 262–264 °C; IR (KBr) υ_max_ 3206 (NH), 3063 (CH aromatic), 2932 and 2835 (CH aliphatic), 1728 and 1670 (C=O), 1609, 1578, 1512, 1458 cm^−1^; ^1^H NMR (400 MHz; DMSO-*d*_6_) major conformer *δ*_H_ 3.79 (s, 6H), 4.71 (s, 2H), 5.53 (s, 2H), 6.89 (d, ^3^*J* = 8.4 Hz, 1H), 7.09 (d, ^3^*J* = 8.8 Hz, 2H), 7.17 (dd, ^3^*J* = 8.0 Hz, ^4^*J* = 1.2 Hz, 1H), 7.35 (d, ^4^*J* = 1.6 Hz, 1H), 7.67 (d, ^3^*J* = 8.8 Hz, 2H), 7.96 (s, 2H), 8.05 (s, 1H), 11.72 (s, 1H), 12.97 ppm (br., 1H); ^1^H NMR (400 MHz; DMSO-*d*_6_) minor conformer *δ*_H_ 3.81 (s, 3H), 3.83 (s, 3H), 4.72 (s, 2H), 5.06 (s, 2H), 6.91 (d, ^3^*J* = 8.0 Hz, 1H), 7.14 (d, ^3^*J* = 8.4 Hz, 2H), 7.17 (dd, ^3^*J* = 8.3 Hz, ^4^*J* = 1.2 Hz,1H), 7.32 (d, ^4^*J* = 1.6 Hz, 1H), 7.70 (d, ^3^*J* = 8.8 Hz, 2H), 7.97 (s, 2H), 8.15 (s, 1H), 11.76 (s, 1H), 12.97 ppm (br., 1H); ^13^C NMR (100 MHz; DMSO-*d*_6_) major conformer *δ*_C_ 46.37, 55.36, 55.64, 64.94, 109.36, 112.75, 112.81, 114.40, 119.90, 121.20, 121.53, 124.51, 124.74, 127.24, 130.51, 136.54, 142.00, 144.50, 149.09, 149.13, 156.00, 160.78, 168.00, 169.99 ppm; ^13^C NMR (100 MHz; DMSO-*d*_6_) minor conformer *δ*_C_ 46.60, 55.43, 55.57, 64.92, 108.94, 112.55, 112.69, 114.44, 120.03, 121.31, 121.70, 124.67, 124.80, 127.20, 130.71, 136.32, 142.00, 144.50, 147.88, 149.30, 155.97, 160.87, 163.06, 169.99 ppm; HRMS (−) ESI *m/z* Calculated for C_26_H_21_Cl_2_N_4_O_6_ [M−H]^−^: 555.0838, Found: 555.0853.

##### 2-(5-((2-(2-(5,6-Dichloro-2-(4-methoxyphenyl)-1*H*-benzo[*d*]imidazol-1-yl)acetyl)hydrazono)methyl)-2-methoxyphenoxy)acetic acid (10n)

A white precipitate was obtained in a yield of 80%; mp 260–263 °C; IR (KBr) υ_max_ 3267 and 3167 (NH), 3067 (CH aromatic), 2943 and 2839 (CH aliphatic), 1736 and 1694 (C=O), 1609, 1578, 1516, 1458 cm^−1^; ^1^H NMR (400 MHz; DMSO-*d*_6_) major conformer *δ*_H_ 3.79 (s, 3H), 3.81 (s, 3H), 4.69 (s, 2H), 5.51 (s, 2H), 7.03 (d, ^3^*J* = 8.4 Hz, 1H), 7.09 (d, ^3^*J* = 8.8 Hz, 2H), 7.23 (d, ^4^*J* = 2.0 Hz, 1H), 7.25 (s, 1H), 7.66 (d, ^3^*J* = 8.8 Hz, 2H), 7.95 (s, 1H), 7.96 (s, 1H), 8.05 (s, 1H), 11.74 ppm (s, 1H); ^1^H NMR (400 MHz; DMSO-*d*_6_) minor conformer *δ*_H_ 3.82 (s, 3H), 3.83 (s, 3H), 4.71 (s, 2H), 5.09 (s, 2H), 7.05 (d, ^3^*J* = 8.4 Hz, 1H), 7.13 (d, ^3^*J* = 8.8 Hz, 2H), 7.19 (d, ^4^*J* = 2.0 Hz, 1H), 7.25 (ov. s, 1H), 7.72 (d, ^3^*J* = 8.4 Hz, 2H), 7.98 (s, 2H), 8.19 (s, 1H), 12.07 (s, 1H); ^13^C NMR (100 MHz; DMSO-*d*_6_) major conformer *δ*_C_ 46.23, 55.33, 55.69, 65.13, 110.45, 112.02, 112.79, 114.39, 119.88, 121.47, 121.81, 124.47, 124.71, 126.48, 130.46, 136.54, 141.98, 144.37, 147.38, 150.76, 155.94, 160.74, 167.85, 170.05 ppm; ^13^C NMR (100 MHz; DMSO-*d*_6_) minor conformer *δ*_C_ 46.57, 55.40, 55.67, 64.95, 109.91, 111.97, 112.56, 114.39, 119.97, 121.29, 122.46, 124.61, 124.74, 126.43, 130.70, 136.27, 141.97, 147.45, 147.78, 150.94, 155.97, 160.82, 162.98, 169.97 ppm; HRMS (−) ESI *m/z* Calculated for C_26_H_21_Cl_2_N_4_O_6_ [M−H]^−^: 555.0838, Found: 555.0897.

##### Methyl-2-(5-((2-(2-(5,6-dichloro-2-(4-methoxyphenyl)-1*H*-benzo[*d*]imidazol-1-yl)acetyl)hydrazono)methyl)-2-methoxyphenoxy)acetate (10o)

A buff precipitate was obtained in a yield of 69%; mp 186–188 °C; IR (KBr) υ_max_ 3221 (NH), 3070 (CH aromatic), 2920 (CH aliphatic), 1739 and 1674 (C=O), 1516, 1458 cm^−1^; ^1^H NMR (500 MHz; DMSO-*d*_6_) major conformer *δ*_H_ 3.61 (s, 3H), 3.80 (s, 3H), 3.82 (s, 3H), 4.80 (s, 2H), 5.51 (s, 2H), 7.04–7.14 (m, 3H), 7.27 (br, 2H), 7.66 (d, ^3^*J* = 7.0 Hz, 2H), 7.96 (s, 2H), 8.05 (s, 1H), 11.74 ppm (s, 1H); ^1^H NMR (500 MHz; DMSO-*d*_6_) minor conformer *δ*_H_ 3.69 (s, 3H), 3.82 (ov. s, 6H), 4.80 (ov. s, 2H), 5.06 (s, 2H), 7.04–7.14 (ov. m, 3H), 7.22 (br., 2H), 7.70 (d, ^3^*J* = 8.5 Hz, 2H), 7.94 (s, 2H), 8.14 (s, 1H), 11.74 ppm (ov. s, 1H); ^13^C NMR (125 MHz; DMSO-*d*_6_) major conformer *δ*_C_ 46.18, 51.59, 55.26, 55.67, 65.24, 110.86, 112.14, 112.69, 114.29, 119.83, 121.44, 122.07, 124.41, 124.64, 126.48, 130.37, 136.46, 141.96, 144.17, 150.80, 155.83, 160.69, 167.82, 168.99 ppm; ^13^C NMR (125 MHz; DMSO-*d*_6_) minor conformer *δ*_C_ 46.55, 51.72, 55.32, 55.70, 65.26, 110.87, 112.43, 112.53, 114.31, 119.94, 121.48, 122.48, 124.43, 124.68, 126.45, 130.60, 136.50, 141.98, 144.20, 147.12, 150.99, 155.84, 160.78, 168.99 ppm; HRMS (−) ESI *m/z* Calculated for C_27_H_23_Cl_2_N_4_O_6_ [M−H]^−^: 569.0995, Found: 569.1019.

##### Methyl-2-(4-((2-(2-(5,6-dichloro-2-(4-methoxyphenyl)-1*H*-benzo[*d*]imidazol-1-yl)acetyl)hydrazono)methyl)-2-methoxyphenoxy)acetate (10p)

A white precipitate was obtained in a yield of 77%; mp 190–192 °C; IR (KBr) υ_max_ 3198 (NH), 3055 (CH aromatic), 2935 and 2839 (CH aliphatic), 1751 and 1663 (C=O), 1613, 1577, 1512, 1458 cm^−1^; ^1^H NMR (400 MHz; DMSO-*d*_6_) major conformer *δ*_H_ 3.70 (s, 3H), 3.79 (s, 3H), 3.81 (s, 3H), 4.84 (s, 2H), 5.53 (s, 2H), 6.92 (d, ^3^*J* = 8.4 Hz, 1H), 7.09 (d, ^3^*J* = 8.8 Hz, 2H), 7.18 (dd, ^3^*J* = 8.4 Hz, ^4^*J* = 1.2 Hz, 1H), 7.36 (d, ^4^*J* = 1.2 Hz, 1H), 7.66 (d, ^3^*J* = 8.8 Hz, 2H), 7.95 (s, 1H), 7.96 (s, 1H), 8.05 (s, 1H), 11.74 ppm (s, 1H); ^1^H NMR (400 MHz; DMSO-*d*_6_) minor conformer *δ*_H_ 3.71 (s, 3H), 3.83 (s, 3H), 3.85 (s, 3H), 4.94 (s, 2H), 5.06 (s, 2H), 6.94 (ov. d, ^3^*J* = 8.4 Hz, 1H), 7.13 (d, ^3^*J* = 8.8 Hz, 2H), 7.17 (ov. dd, ^3^*J* = 8.4 Hz, ^4^*J* = 1.2 Hz, 1H), 7.33 (d, ^4^*J* = 1.2 Hz, 1H), 7.70 (d, ^3^*J* = 8.8 Hz, 2H), 7.96 (ov. s, 2H), 8.15 (s, 1H), 11.77 ppm (s, 1H); ^13^C NMR (100 MHz; DMSO-*d*_6_) major conformer *δ*_C_ 46.39, 51.91, 55.37, 55.68, 65.08, 109.48, 112.80, 113.05, 114.40, 119.92, 121.14, 121.53, 124.55, 124.77, 127.58, 130.52, 136.54, 142.01, 144.46, 148.88, 149.15, 156.02, 160.80, 168.04, 169.06 ppm; ^13^C NMR (100 MHz; DMSO-*d*_6_) minor conformer *δ*_C_ 46.60, 52.01, 55.44, 55.61, 65.00, 109.04, 110.22, 112.59, 113.00, 114.45, 120.04, 121.31, 121.69, 124.70, 124.83, 127.55, 130.73, 136.33, 147.85, 149.08, 149.26, 155.98, 160.89, 163.12, 168.74 ppm; HRMS (−) ESI *m/z* Calculated for C_27_H_24_Cl_2_N_4_O_6_ [M−H]^−^: 569.0995, Found: 569.1063.

### Biology

#### Screening of the inhibitory activity of dichlorobenzimidazoles 10a-p on BRAFWT

The inhibitory activities of **10a**–**p** on BRAF_WT_ was examined utilizing BRAF_WT_ assay kit (BPS Biosciences—San Diego—CA—US) (For further information see Additional file A).

#### Growth inhibitory activity on different types of NCI-USA cancer cell lines

The synthesized dichlorobenzimidazoles **10a**–**p** were assayed for their influence on divers cancer cell lines according to the method presented in the Additional file A.

#### In vitro anticancer screening of 10h on HSF cell line

The dichlorobenzimidazole derivative **10h** was tested in Nawah scientific—Cairo—Egypt for its cytotoxic activity on HSF cell line as stated in the Additional file A [53, 54].

#### Cell cycle analysis assay

The distribution of cells in different stages of the cell cycle of HT29 cell line was detected before and after treatment with **10h** at its GI_50_ concentration (For further details see Additional file A) [55, 56].

#### Apoptosis assay

As stated in the Additional file A, the populations of apoptotic and necrotic cells of HT29 cell line were detected after treatment with **10h** employing Annexin V-FITC apoptosis detection kit (For further details see Additional file A).

### Molecular modeling

First molecular docking was carried out using Molecular Operating Environment (MOE 2022.02) to perform ligand placement of compound **10h** in the target kinase domains. Starting from the obtained molecular docking **10h**/BRAF and **10h**/BRAF_V600E_ complexes, MD simulations were performed using Groningen Machine for Chemical Simulations (GROMACS) 2021.3 package [43]. The obtained poses in BRAF_WT_ and BRAF_V600E_ kinase domains were scored using Molecular Mechanics/Generalized Born Surface Area (MM/GBSA) binding free energy calculation method implemented in fastDRH webserver (http://cadd.zju.edu.cn/fastdrh/) [44].

#### Molecular docking

MOE 2022.02 were initially used for ligand placement of compound **10h** in BRAF and BRAF_V600E_ kinase domains using the protein structures PDB ID: 1UWH [45] and PDB ID: 1UWJ [45] (See Additional file A).

#### Molecular dynamics simulations

MD simulations for the most promising compound **10h**, were carried out in the kinase domains of BRAF_WT/V600E_. MD simulations were performed using GROMACS 2021.3 package [43] for 100 ns starting from the obtained molecular docking **10h**/BRAF and **10h**/BRAF_V600E_ complexes. The obtained poses of compound **10h** in BRAF and BRAF_V600E_ kinase domains in the dominant clusters were scored using Molecular Mechanics/Generalized Born Surface Area (MM/GBSA) binding free energy calculation method implemented in fastDRH webserver (http://cadd.zju.edu.cn/fastdrh/) [44] and were compared to that of the co-crystalized ligand sorafenib in its experimental poses (See Additional file A).

#### Physicochemical and pharmacokinetic properties prediction

SwissADME online web tool was used to predict the physicochemical and ADME properties of the target compounds **10a**–**p**. The compounds’ SMILES were produced using (MOE, 2022.02) software then they were submitted to the SwissADME [38, 50, 57].

## Supplementary Information

Additional file 1.

## Data Availability

Data is provided within the supplementary information file.
