# Supplementary material for: Novel 5,6-dichlorobenzimidazole derivatives as dual BRAFWT and BRAFV600E inhibitors: design, synthesis, anti-cancer activity and molecular dynamics simulations
Source: BMC Chem. 2025 Feb 21;19(1):45. doi: 10.1186/s13065-025-01402-8 (PMC11844072; doi:10.1186/s13065-025-01402-8)
Supplement: Supplementary file 1 — Additional file 1. [file 13065_2025_1402_MOESM1_ESM.pdf]

Supporting Information

Novel 5,6-Dichlorobenzimidazole Derivatives as Dual $BRAF_{WT}$ and $BRAF_{V600E}$ Inhibitors: Design, Synthesis, Anti-cancer Activity and Molecular Dynamics Simulations

Ahmed Temirak[1], Ahmed M. El Kerdawy[2,3], Amira M. Nageeb[4], Heba T. Abdel-Mohsen[1]*

[1]Chemistry of Natural and Microbial Products Department, Pharmaceutical and Drug Industries Research Institute, National Research Centre, Dokki, P.O. 12622, Cairo, Egypt.

[2]School of Pharmacy, College of Health and Science, University of Lincoln, Joseph Banks Laboratories, Green Lane, Lincoln, United Kingdom.

[3]Department of Pharmaceutical Chemistry, Faculty of Pharmacy, Cairo University, Kasr El-Aini Street, P.O. Box 11562, Cairo, Egypt.

[4]High Throughput Molecular and Genetic Technology Lab, Center of Excellence for Advanced Sciences, Biochemistry Department, Biotechnology Research Institute, National Research Centre, Dokki, P.O. 12622, Cairo, Egypt.

* Correspondence: Heba T. Abdel-Mohsen; email address; hebabdelmohsen@gmail.com; ht.abdel-mohsen@nrc.sci.eg

Contents **Page**

**1.**	**IR Spectra of the target dichlorobenzimidazole derivatives 10a-p**	**3**
**2.**	**NMR Spectra of the target dichlorobenzimidazole derivatives 10a-p**	**19**
**3.**	**HRMS spectra of dichlorobenzimidazole derivatives 10a-p**	**51**
**4.**	**Biochemical kinase assays**	**55**
**5.**	**Screening of cytotoxic activity against a panel of sixty human tumor cell lines**	**56**
**5.1.**	**Procedure**	**56**
**5.2.**	**Dose response curves of the dichlorobenzimidazoles 10h, 10o and 10p on NCI cancer cell lines**	**58**
**6.**	**Analysis of 10h on HSF normal cell line**	**61**
**6.1.**	**Procedure**	**61**
**6.2.**	**Dose response curve of 10h on HSF normal cell line**	**61**
**7.**	**Analysis of cell cycle distribution**	**62**
**8.**	**Apoptosis assay**	**62**
**9.**	**Molecular modeling**	**63**
**9.1.**	**Molecular docking**	**63**
**9.2.**	**Molecular dynamic simulations**	**68**
**10.**	**References**	**70**

1. IR Spectra of the target dichlorobenzimidazole derivatives 10a-p

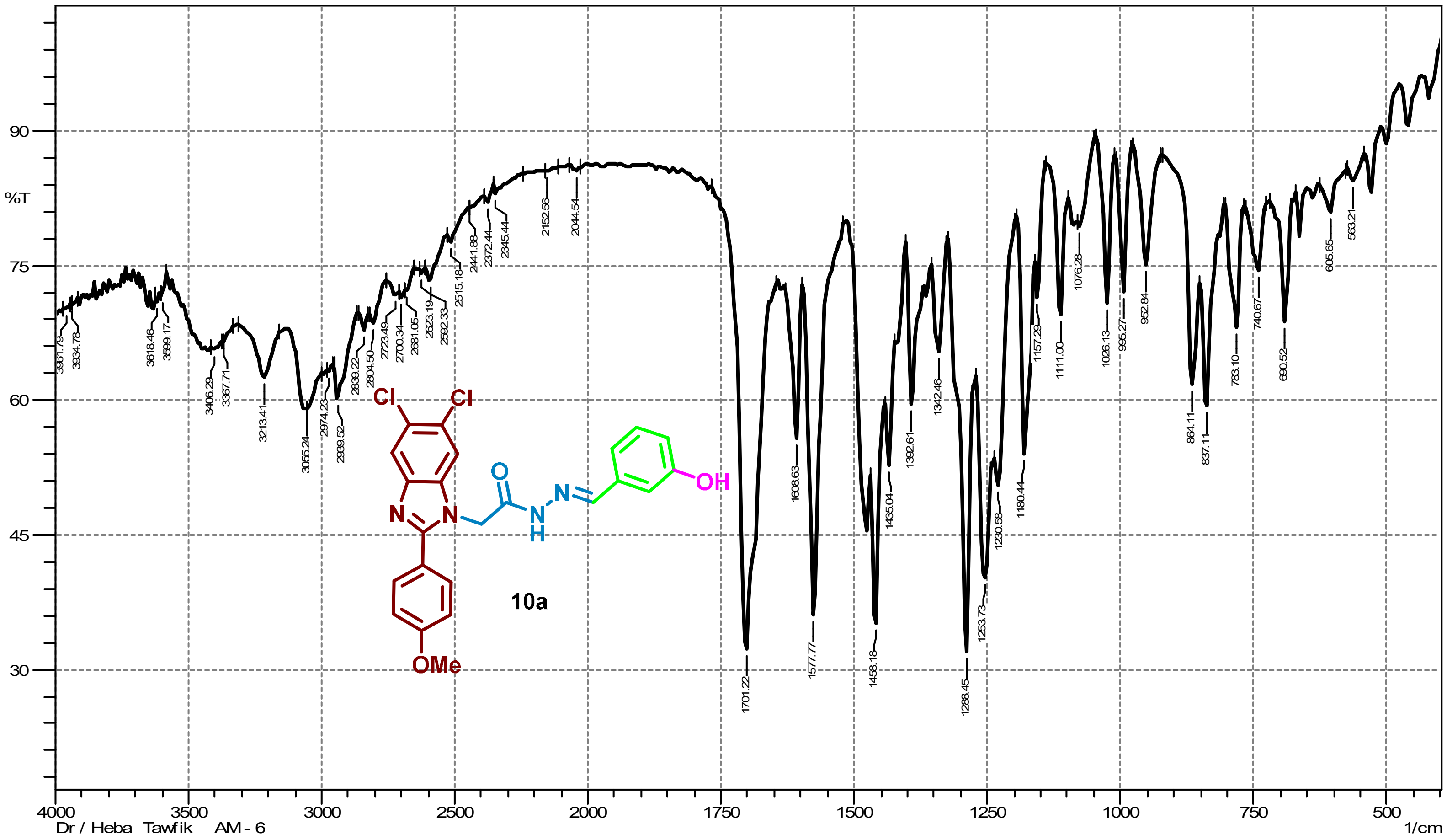

Figure 1. IR spectrum of **10a**

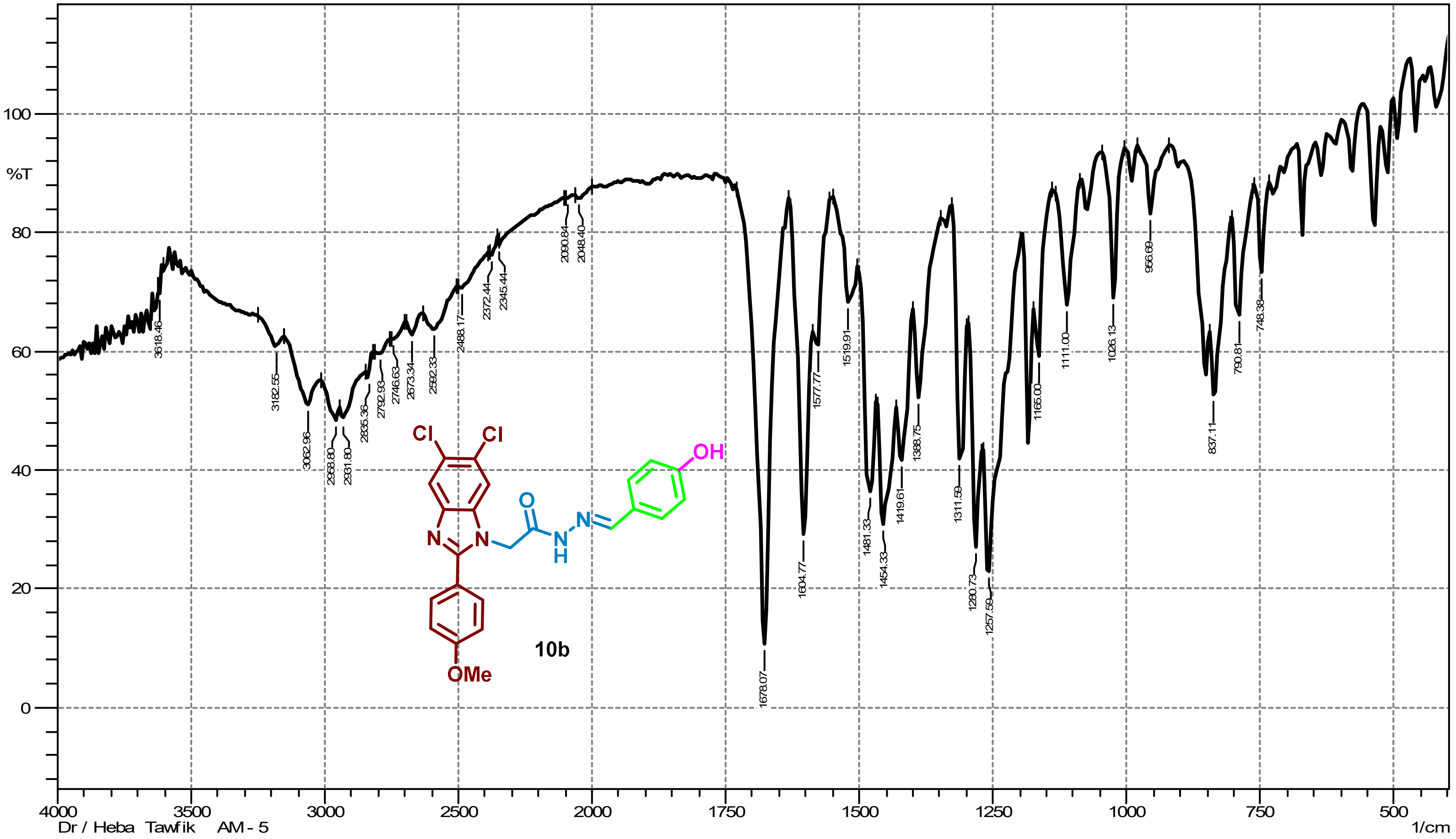

Figure 2. IR spectrum of **10b**

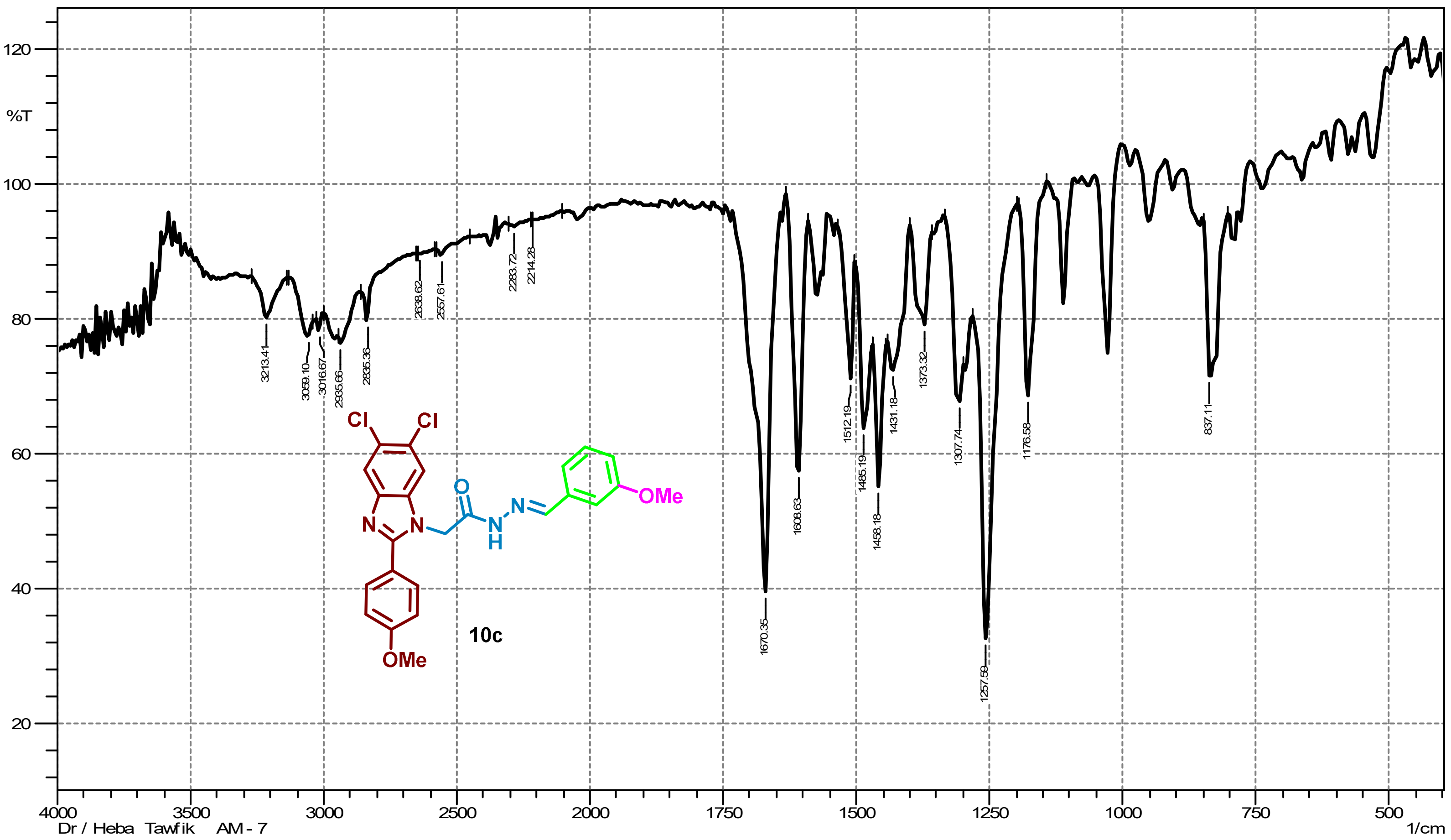

Figure 3. IR spectrum of **10c**

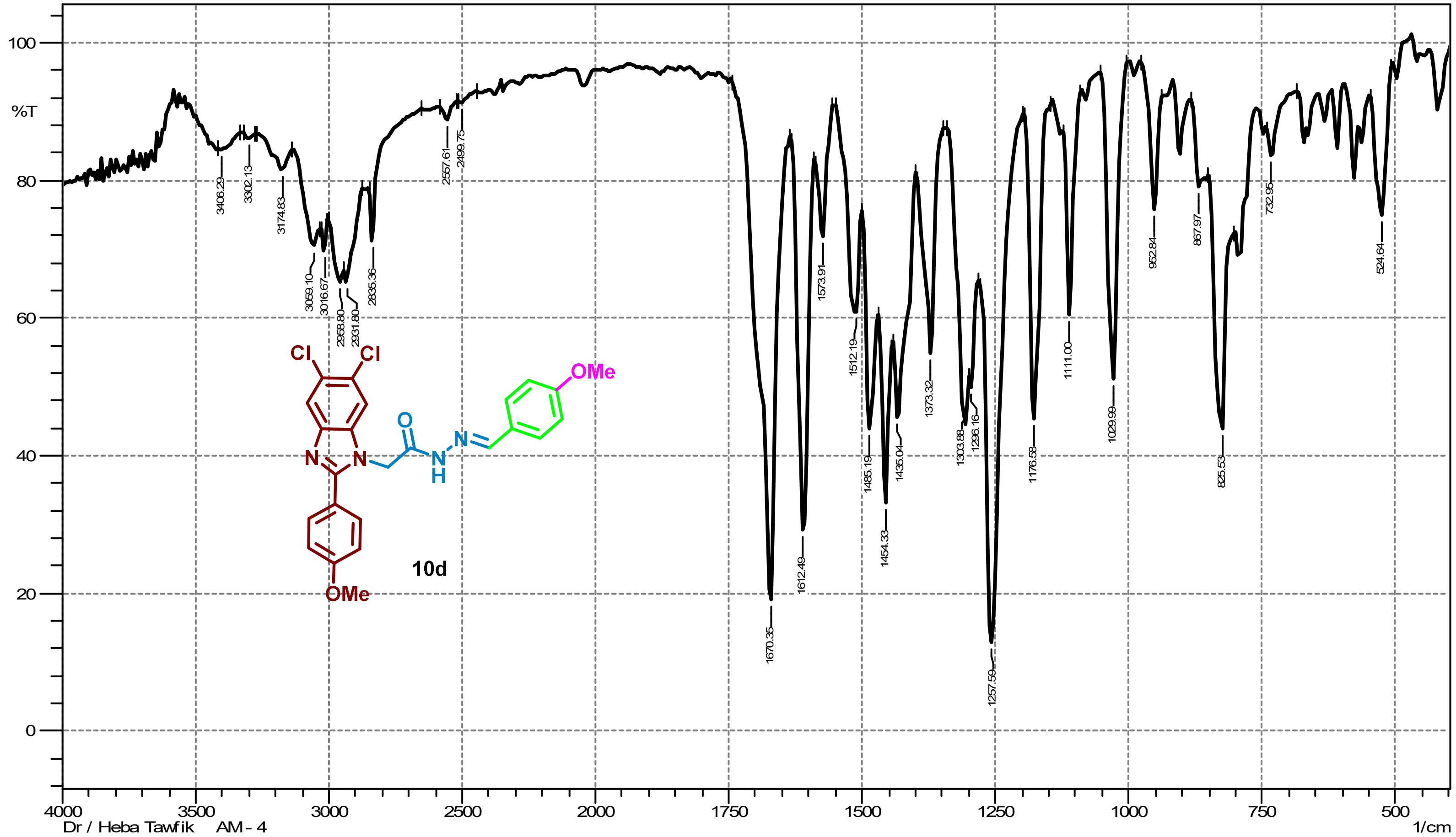

Figure 4. IR spectrum of **10d**

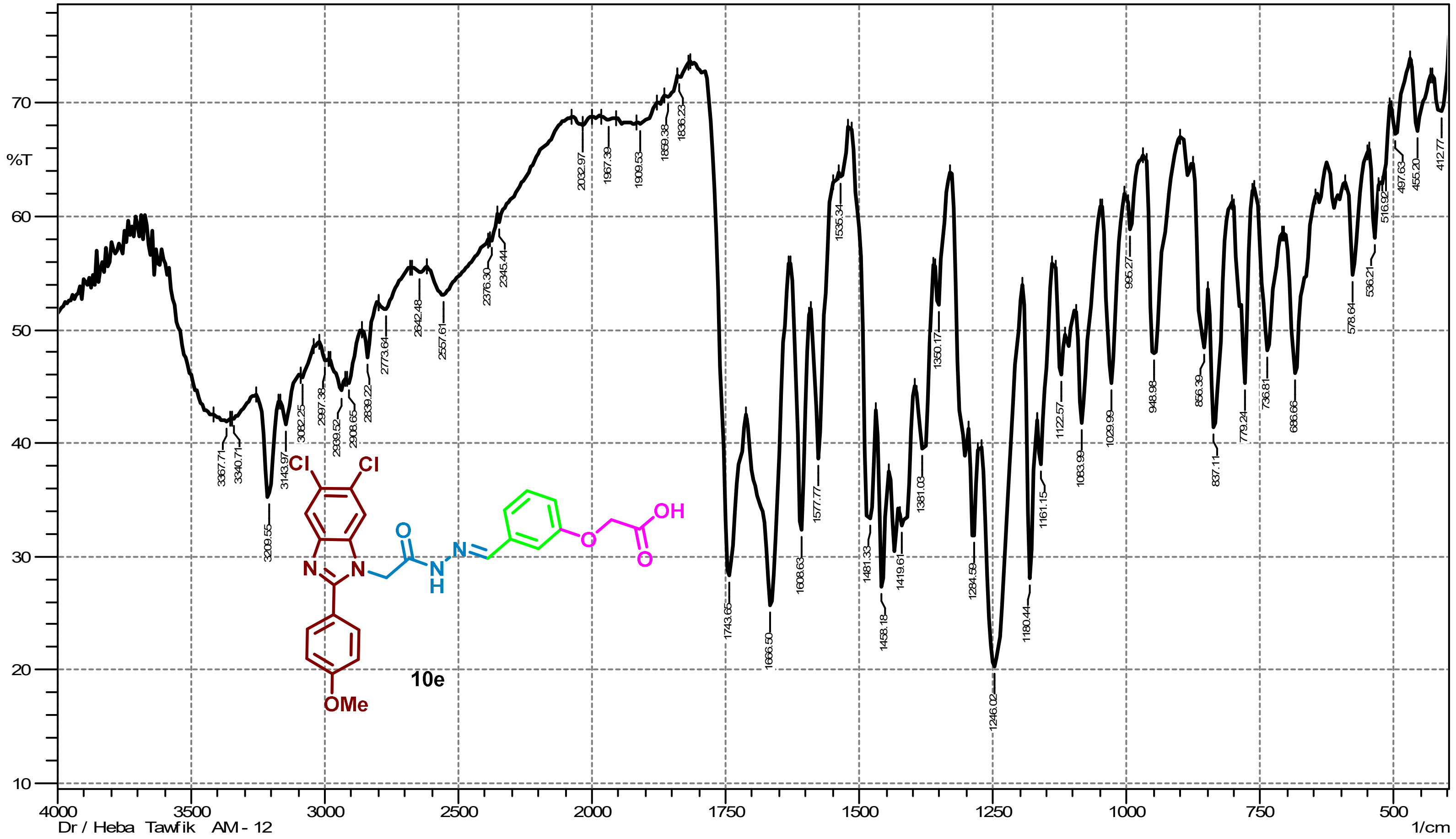

Figure 5. IR Spectrum of **10e**

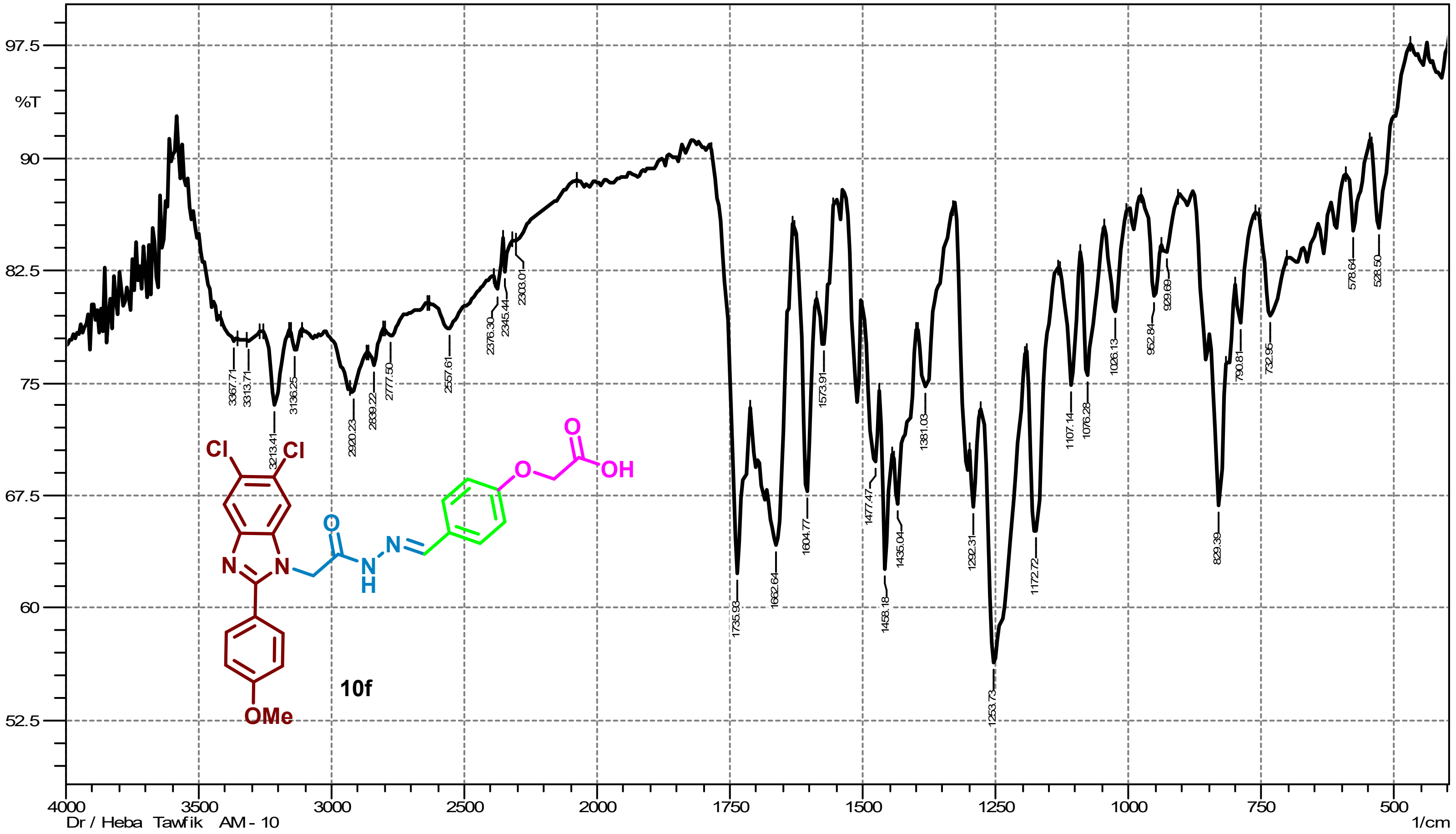

Figure 6. IR spectrum of **10f**

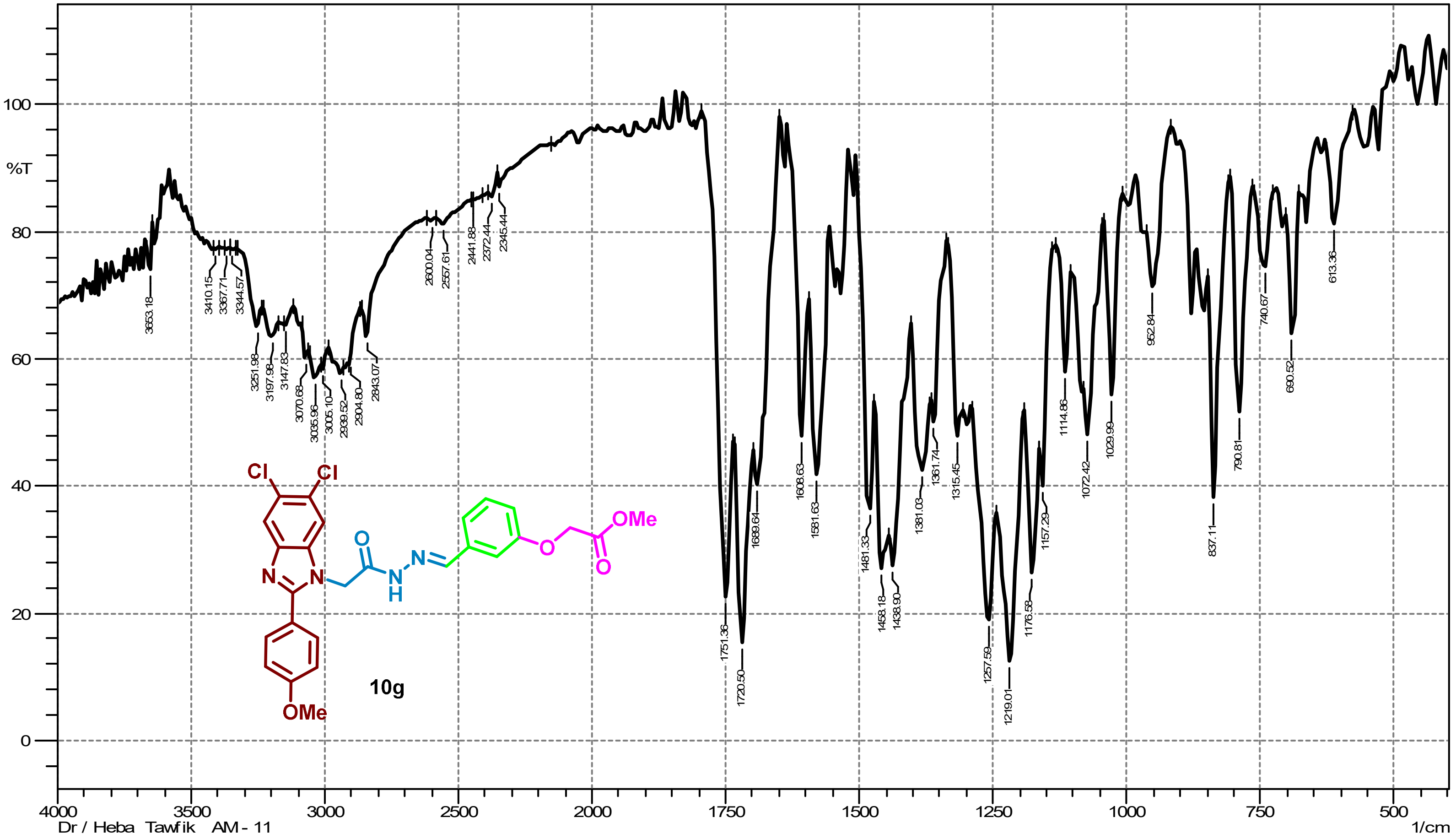

Figure 7. IR spectrum of **10g**

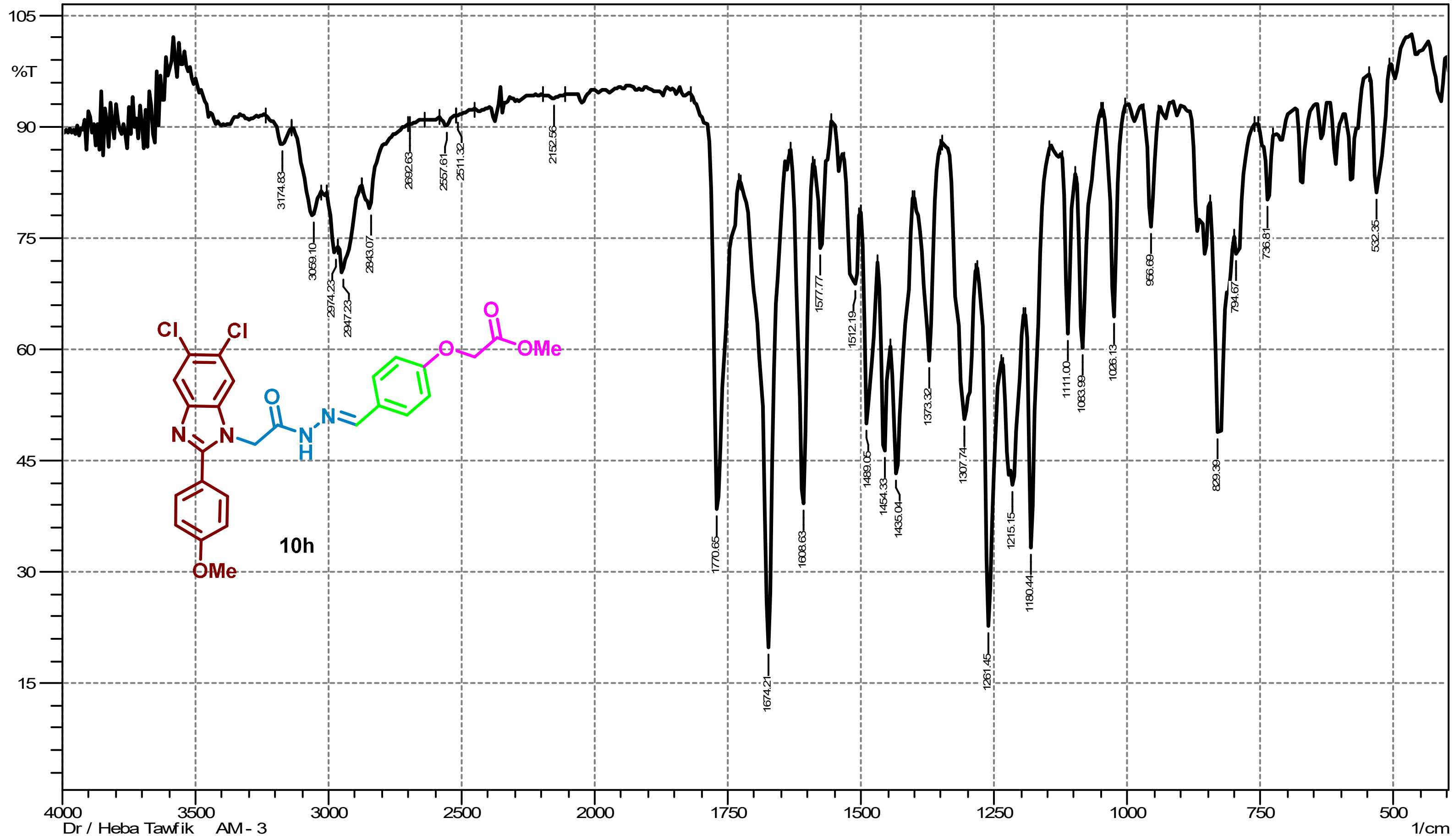

Figure 8. IR spectrum of **10h**

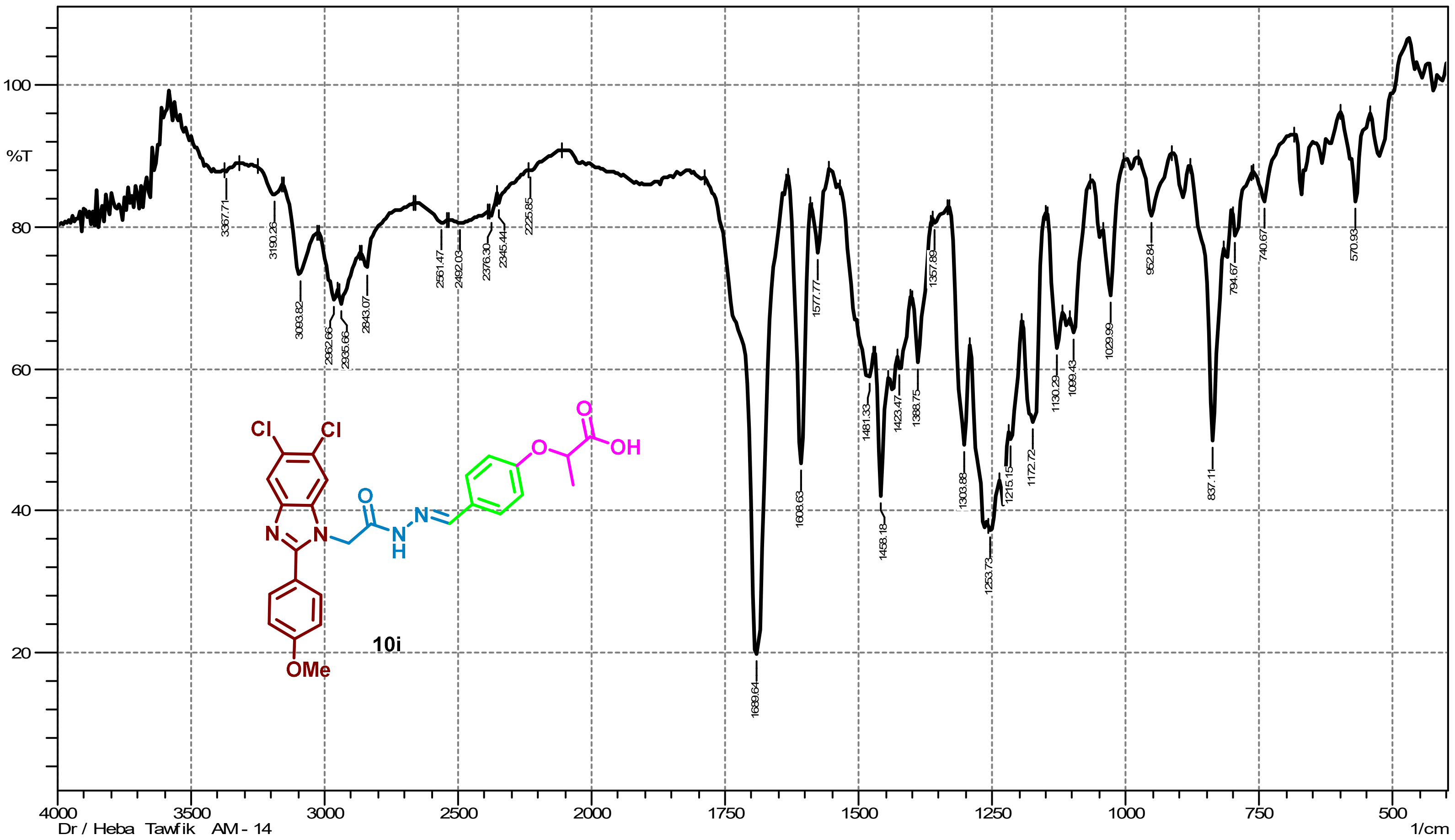

Figure 9. IR spectrum of **10i**

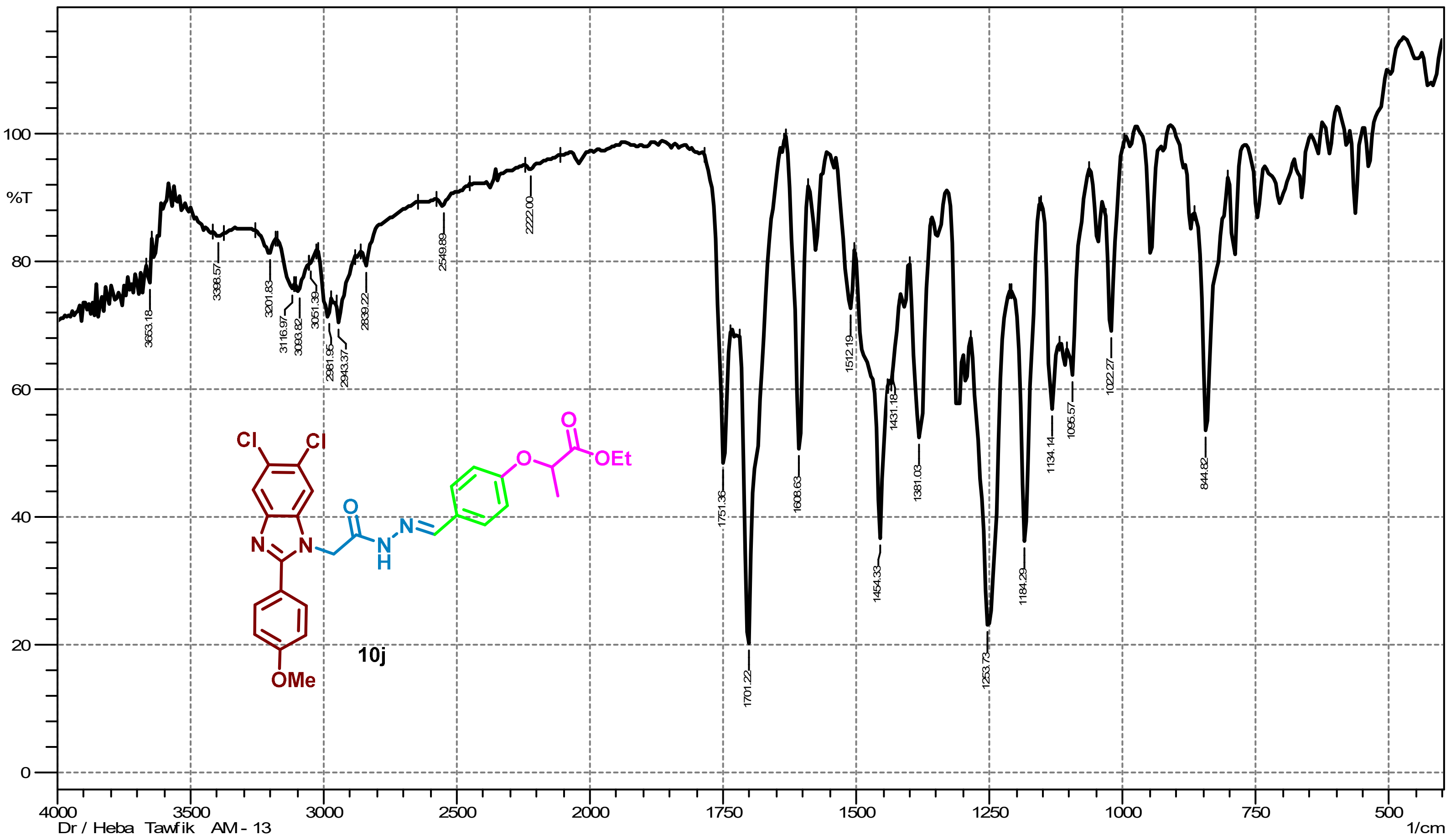

Figure 10. IR spectrum of **10j**

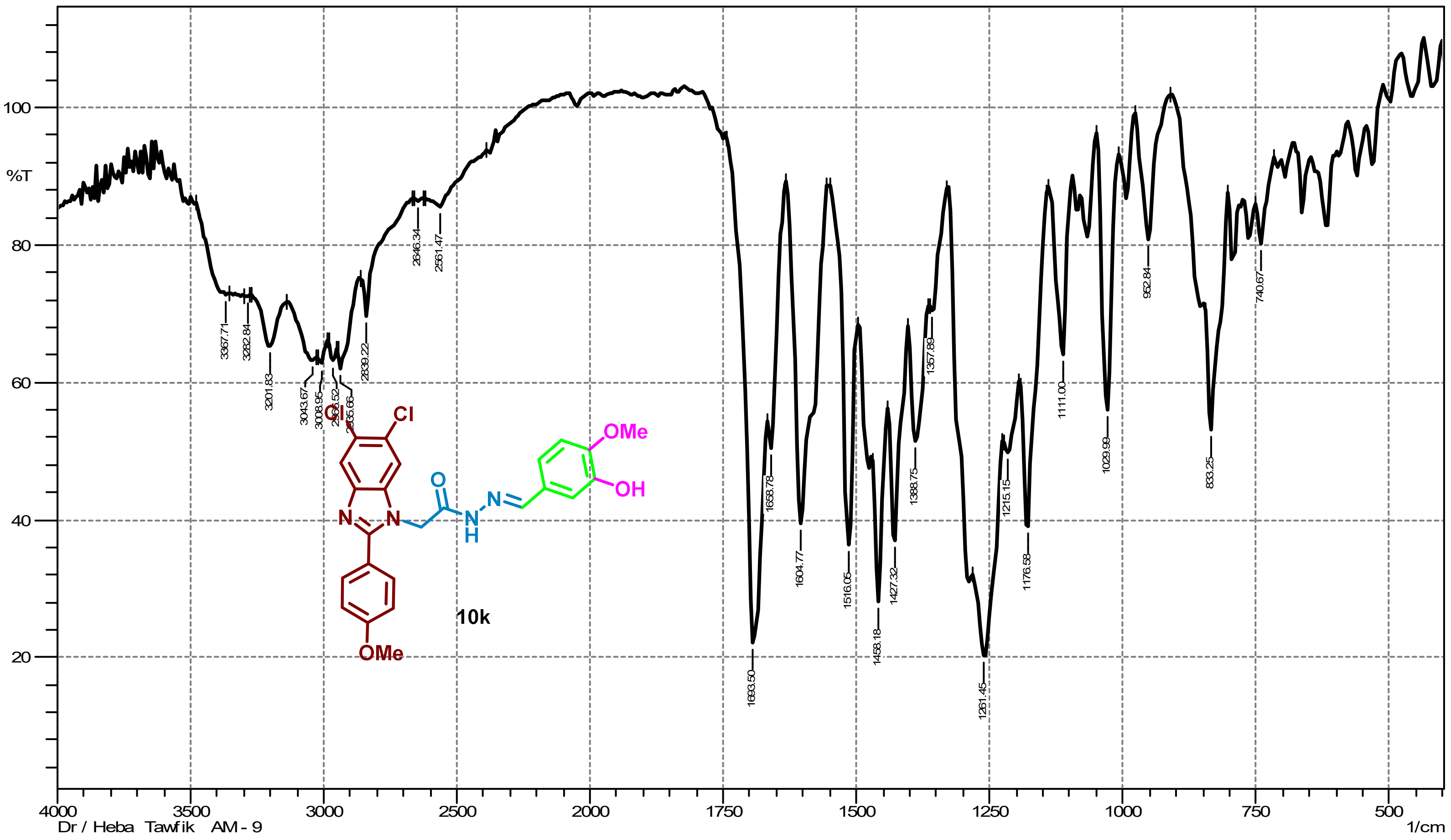

Figure 11. IR spectrum of **10k**

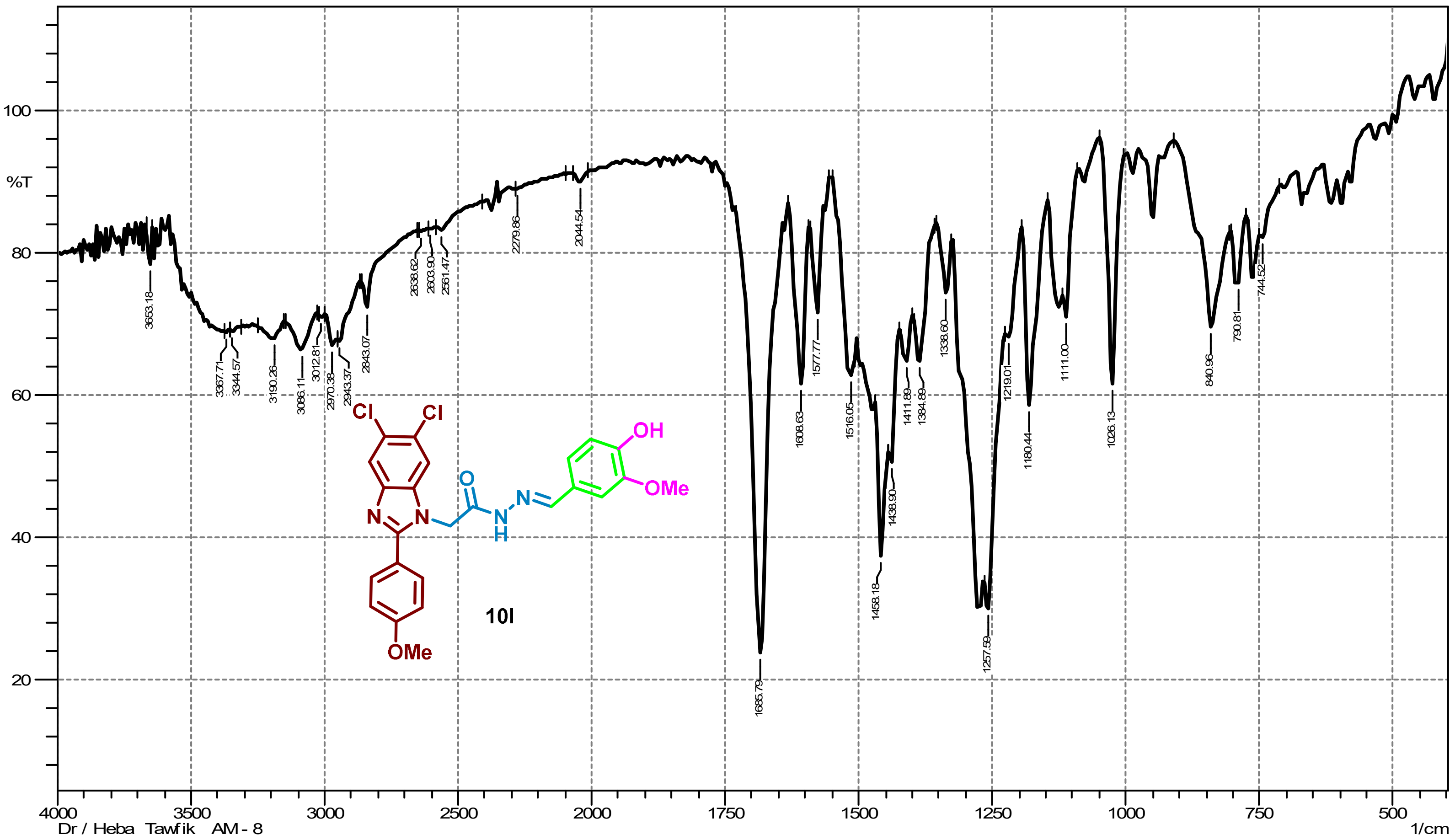

Figure 12. IR spectrum of **10l**

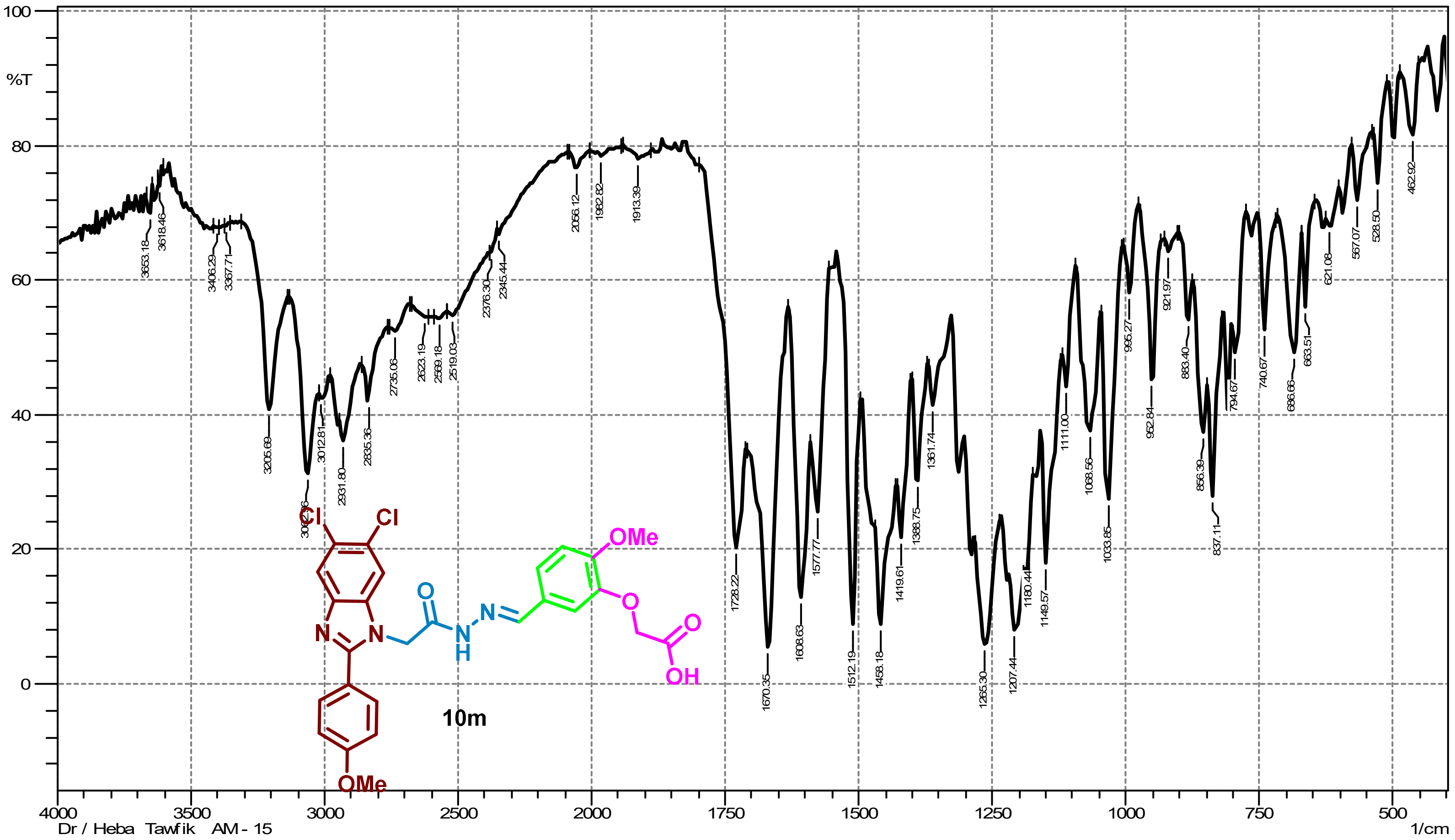

Figure 13. IR spectrum of **10m**

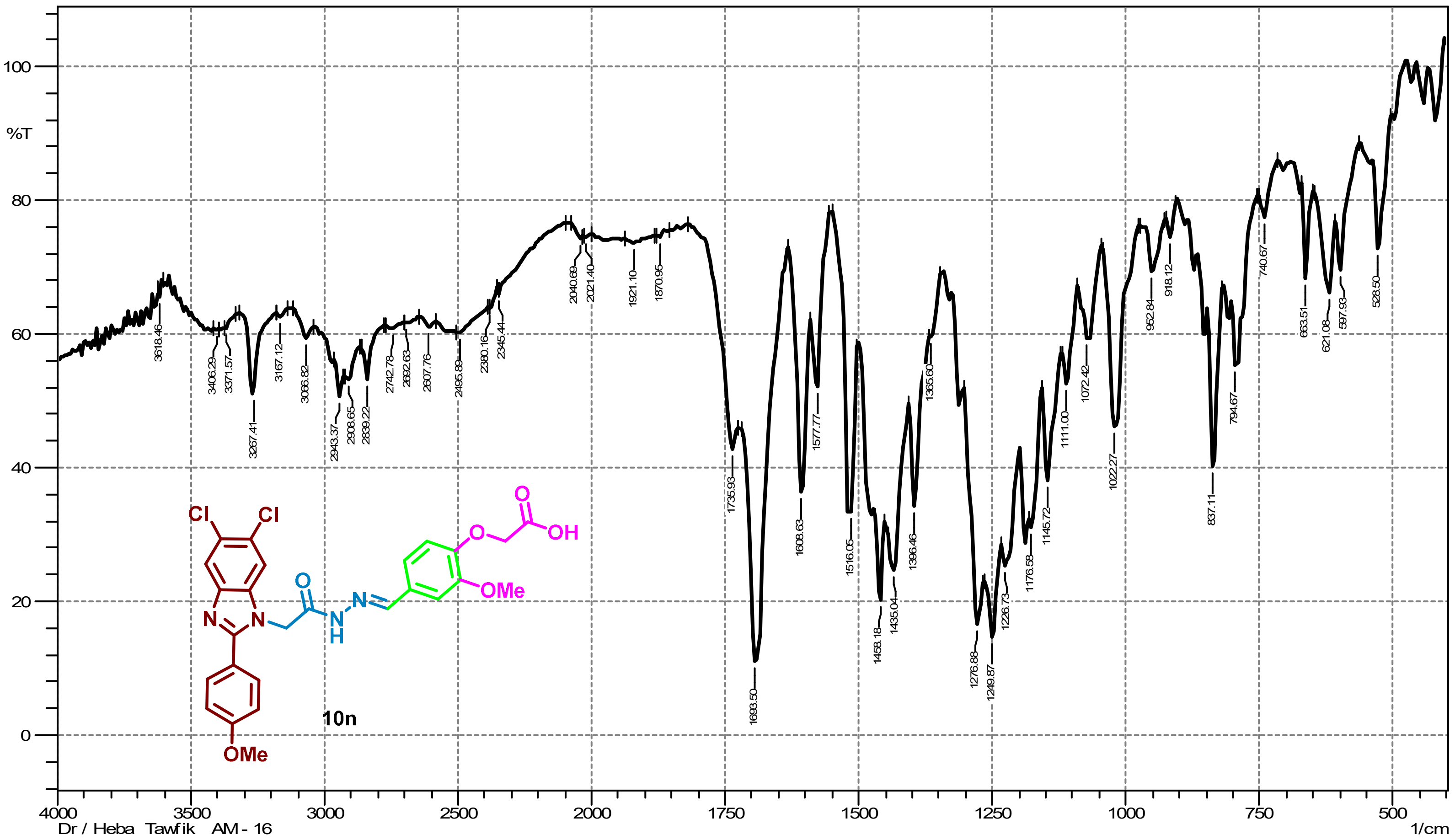

Figure 14. IR spectrum of **10n**

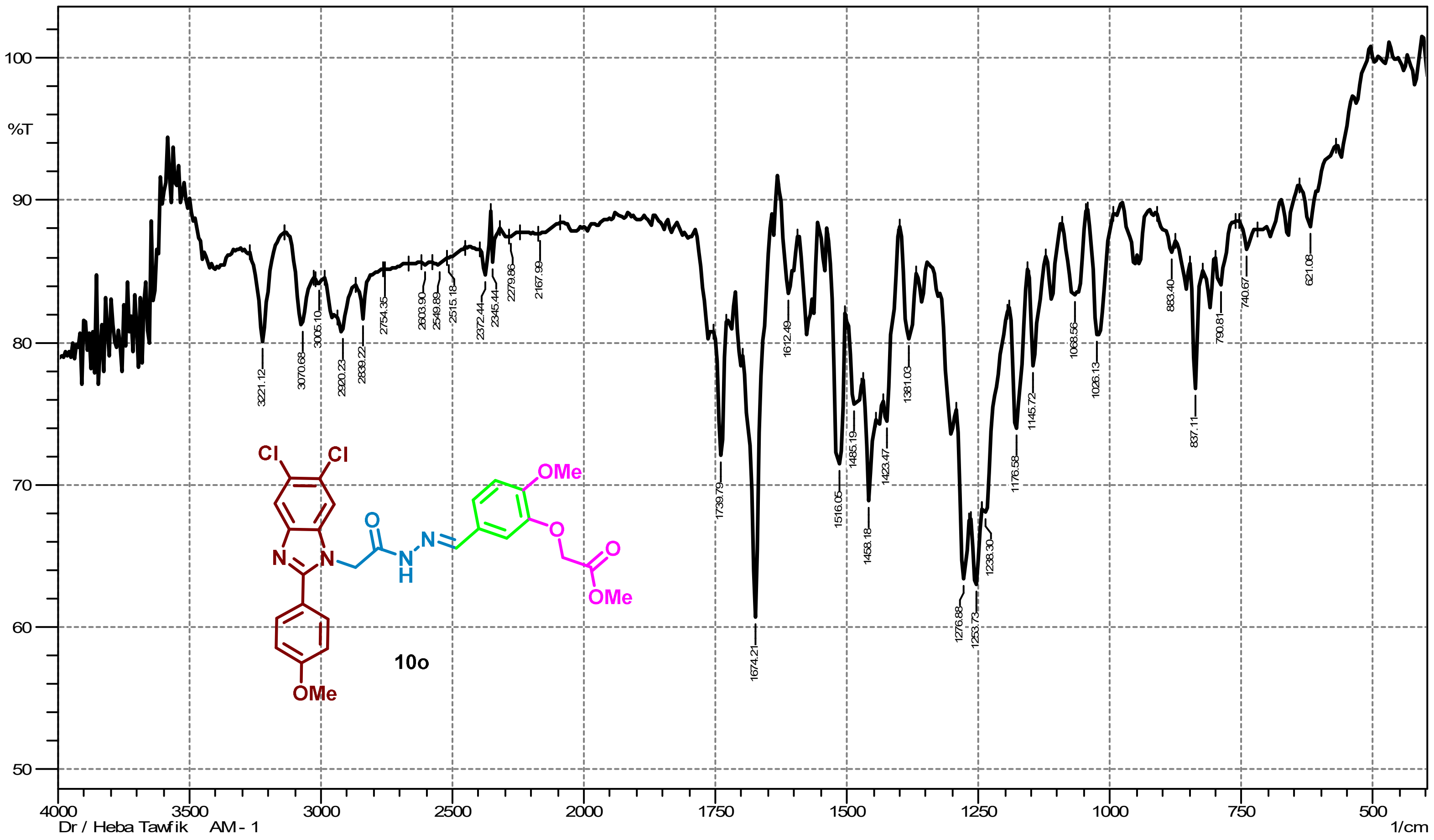

Figure 15. IR spectrum of **10o**

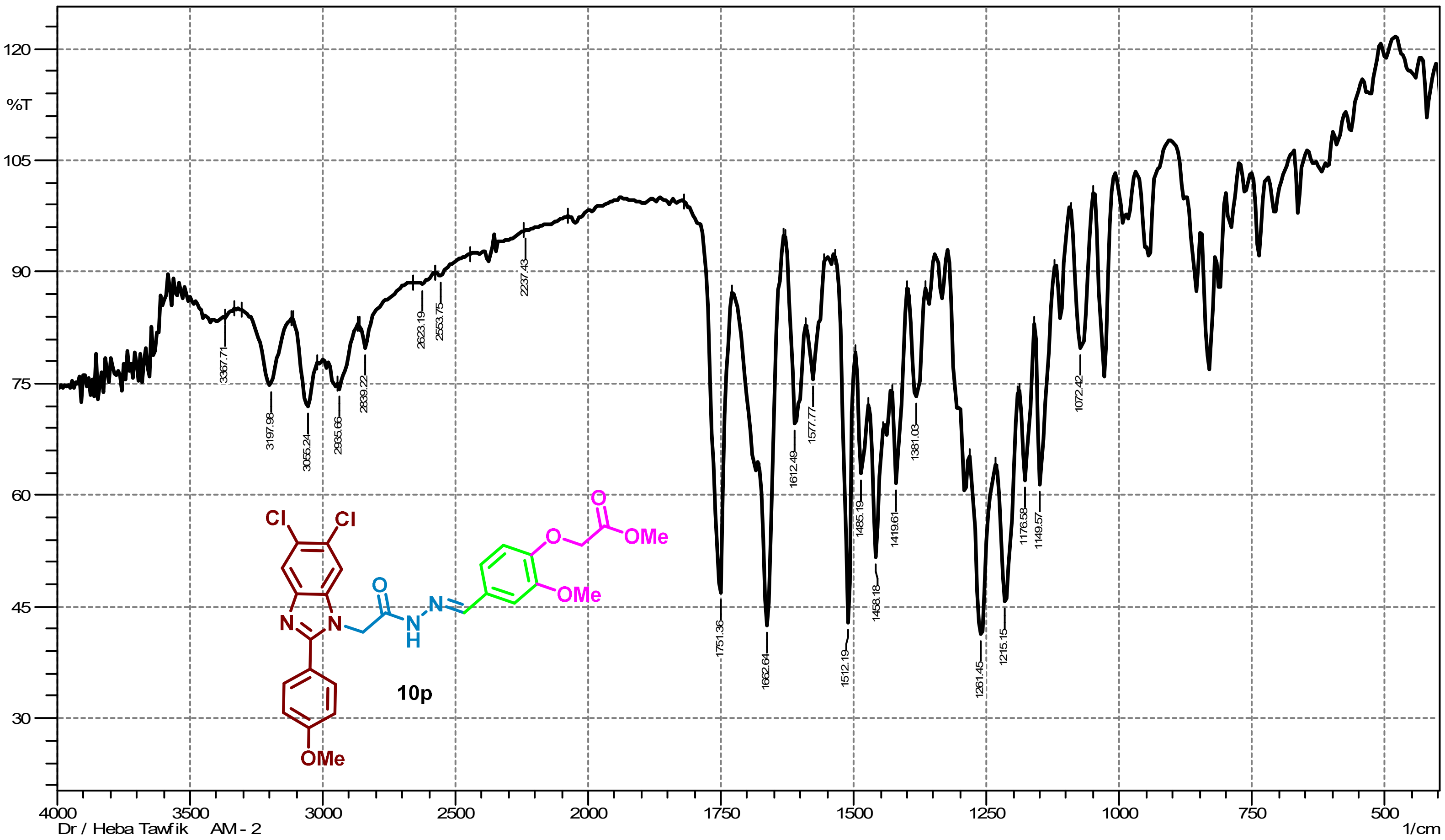

Figure 16. IR spectrum of **10p**

2. NMR Spectra of target dichlorobenzimidazole derivatives 10a-p

2-(5,6-Dichloro-2-(4-methoxyphenyl)-1*H*-benzo[*d*]imidazol-1-yl)-*N*'-(3-hydroxybenzylidene) acetohydrazide (10a)

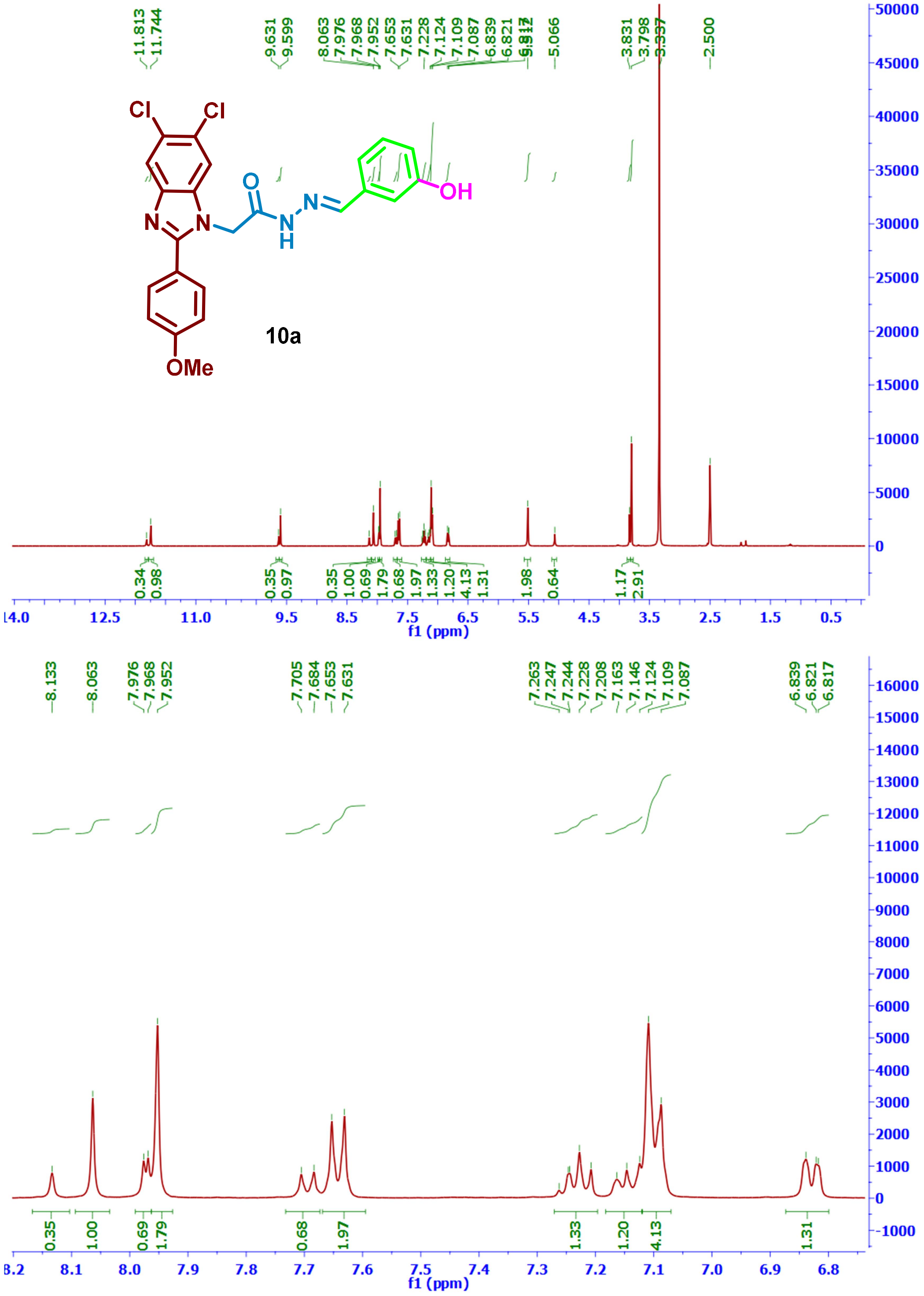

Figure 17. ^{1}H (400 MHz) NMR spectrum of **10a** in DMSO-d_6

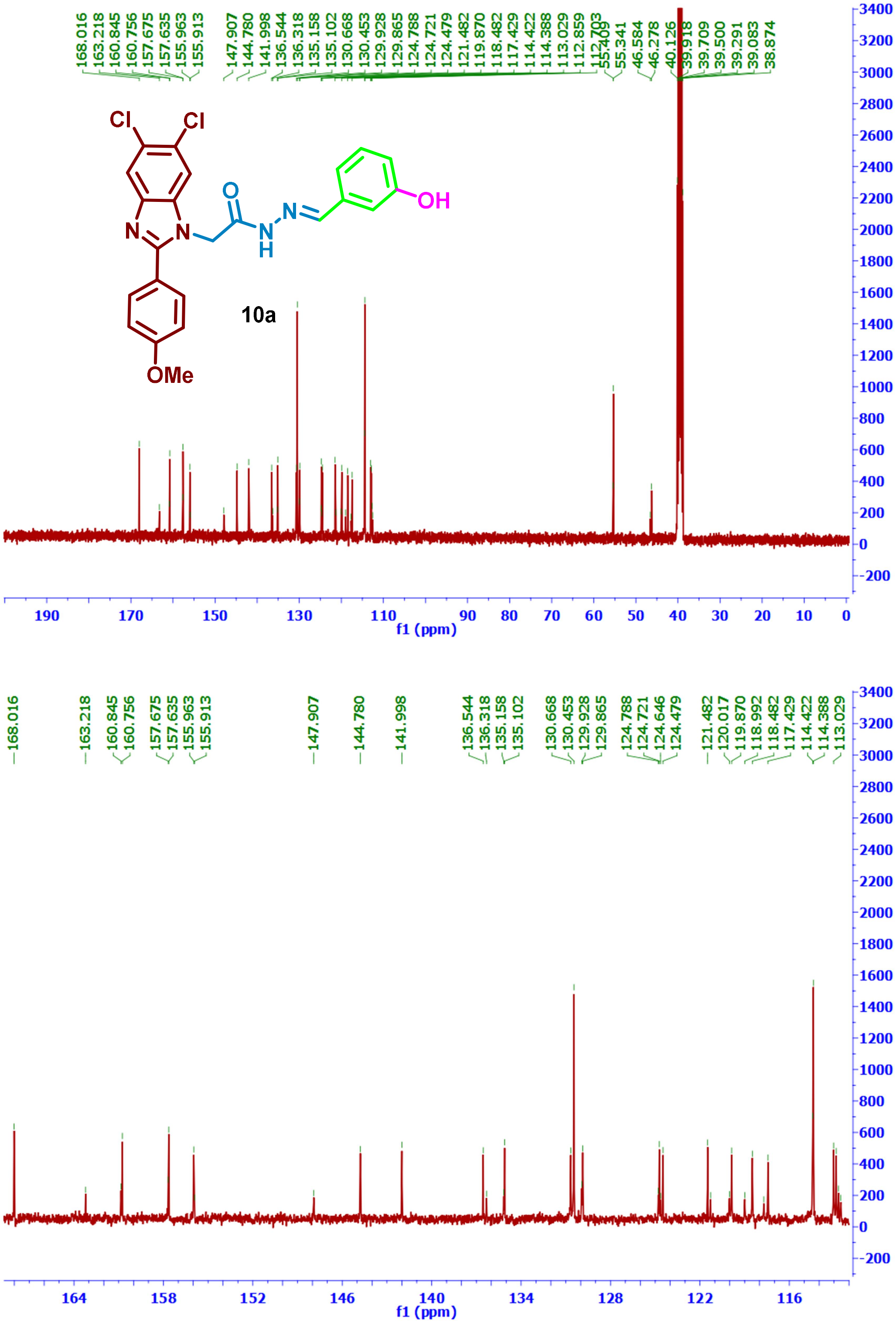

Figure 18. ^{13}C (100 MHz) NMR spectrum of **10a** in DMSO-d_6

2-(5,6-Dichloro-2-(4-methoxyphenyl)-1*H*-benzo[*d*]imidazol-1-yl)-*N*'-(4-hydroxybenzylidene)acetohydrazide (10b)

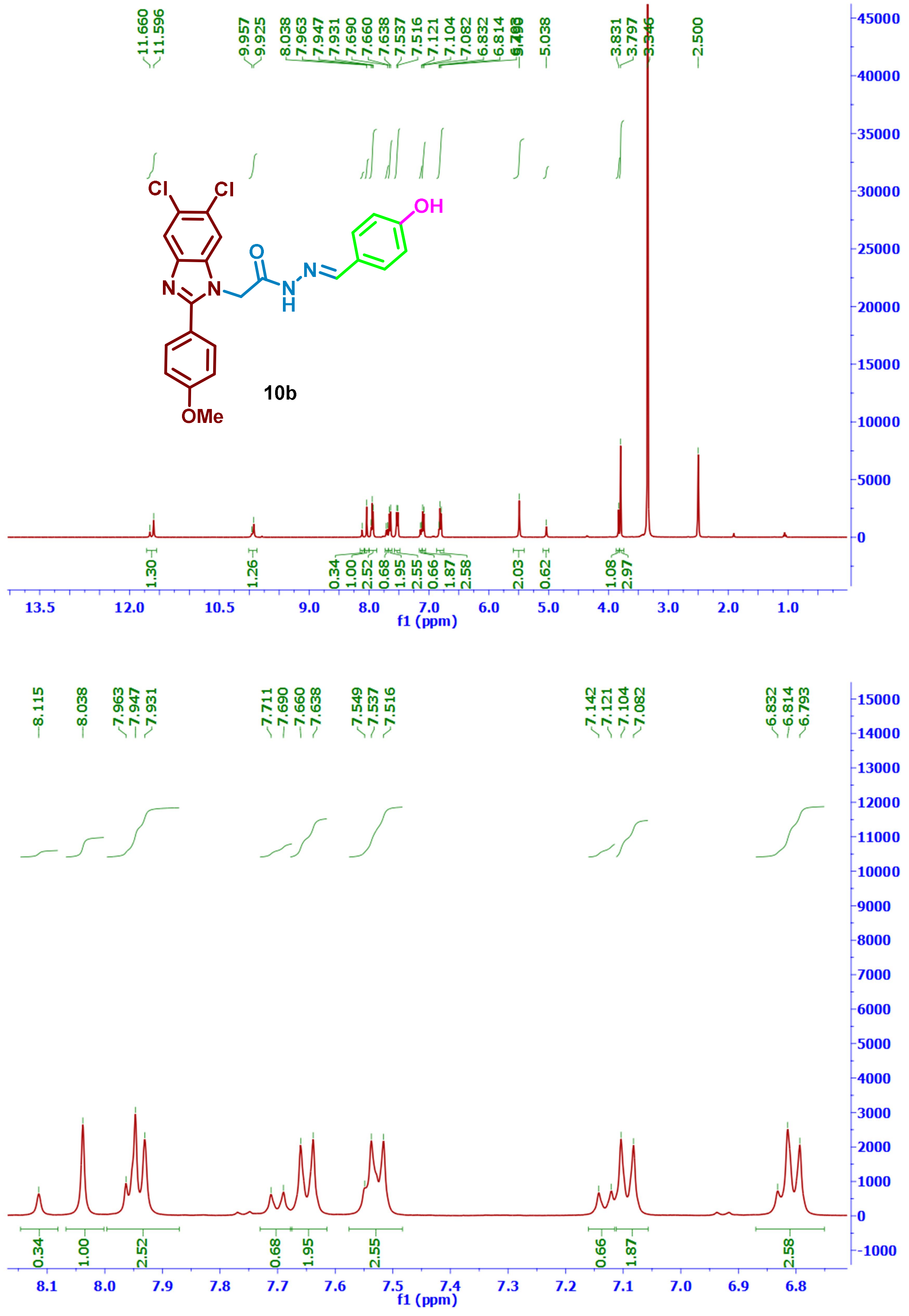

Figure 19. ^{1}H (400 MHz) NMR spectrum of **10b** in DMSO-d_6

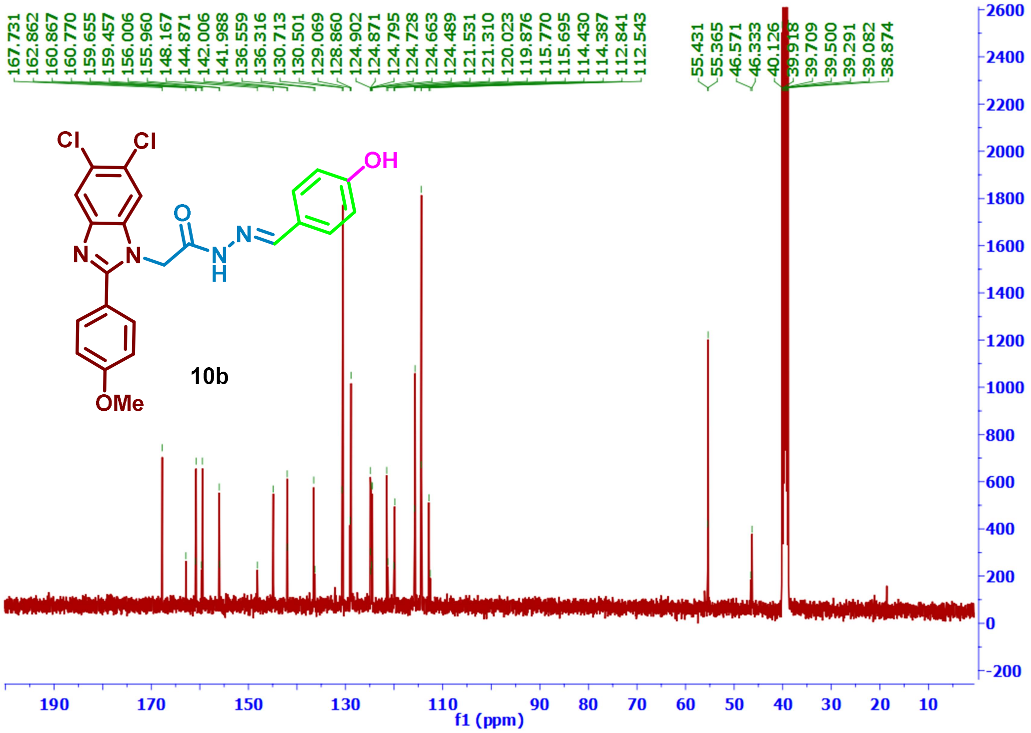

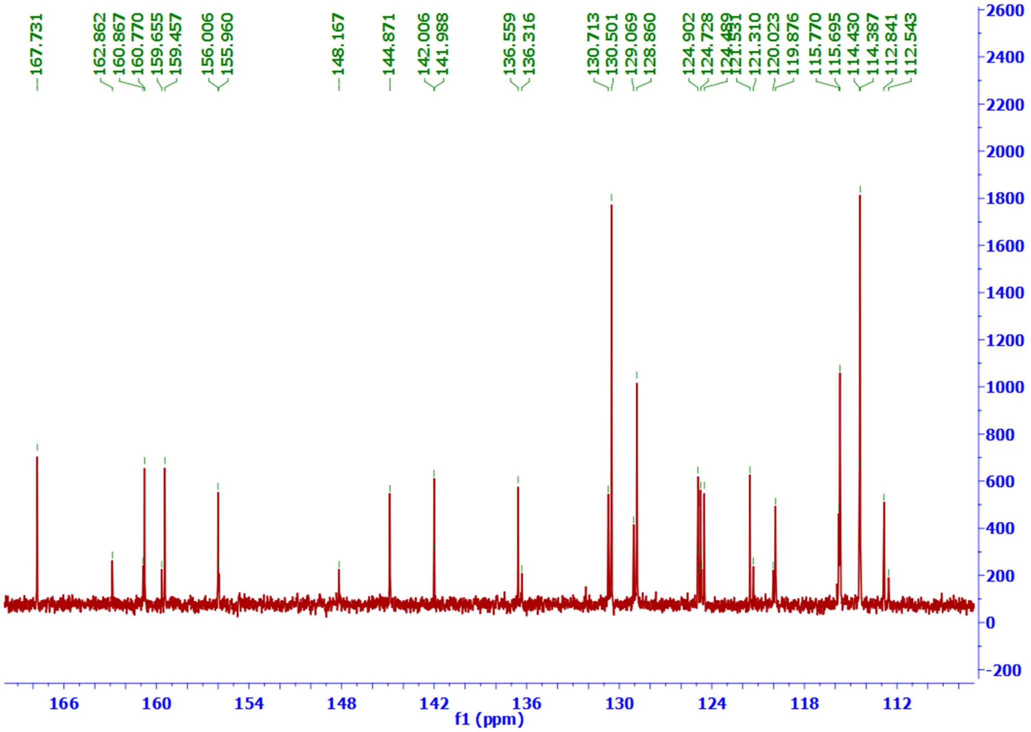

Figure 20. ^{13}C (100 MHz) NMR spectrum of **10b** in DMSO-d_6

2-(5,6-Dichloro-2-(4-methoxyphenyl)-1*H*-benzo[*d*]imidazol-1-yl)-*N*'-(3-methoxybenzylidene)acetohydrazide (10c)

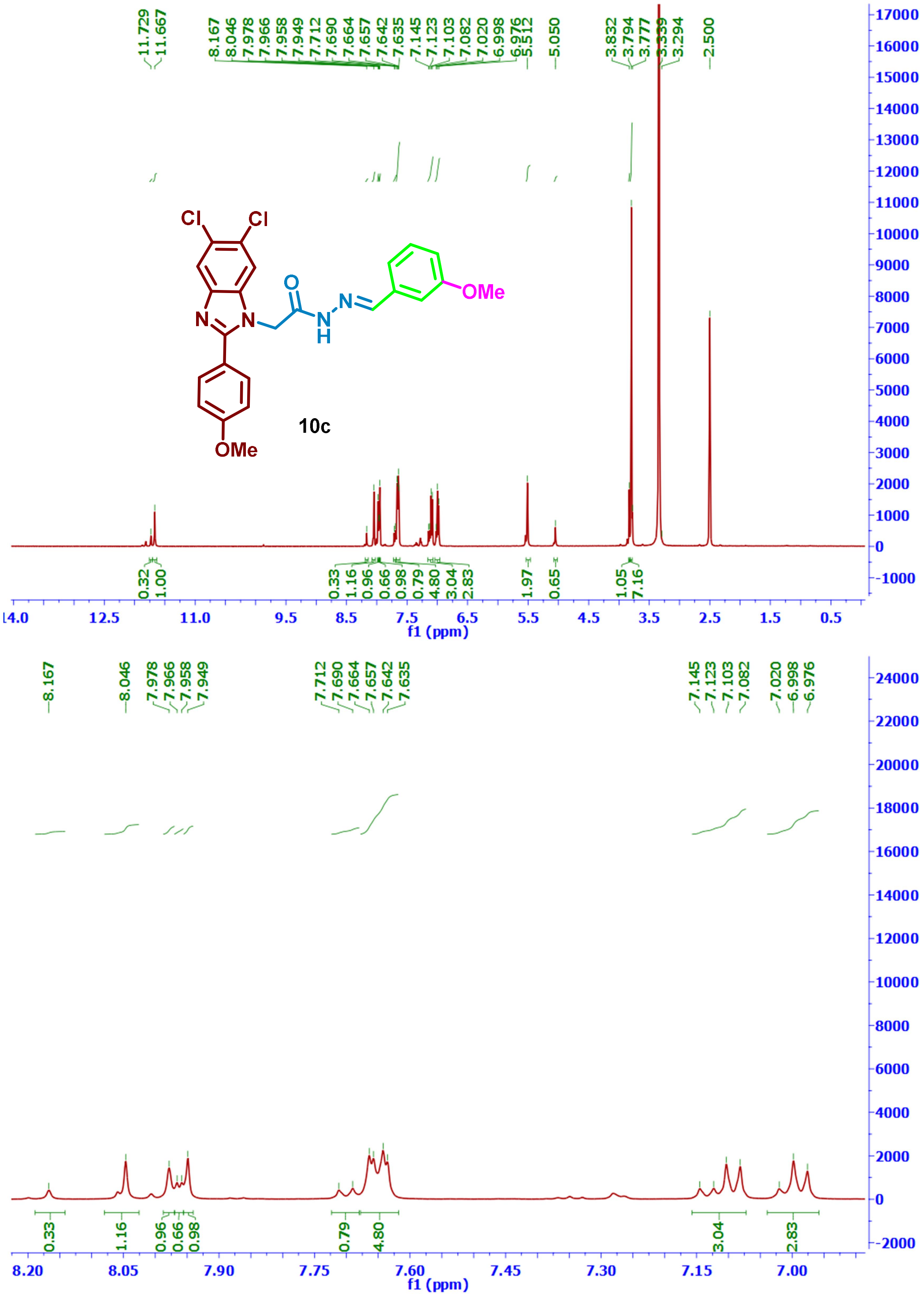

Figure 21. ^{1}H (400 MHz) NMR spectrum of **10c** in DMSO-d_6

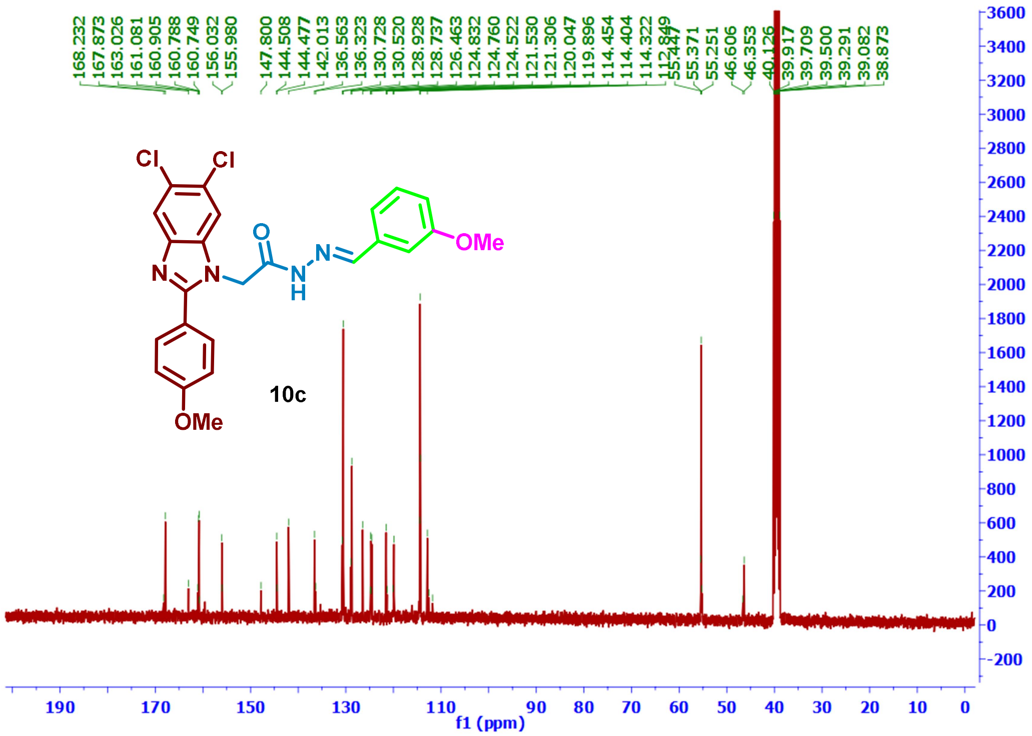

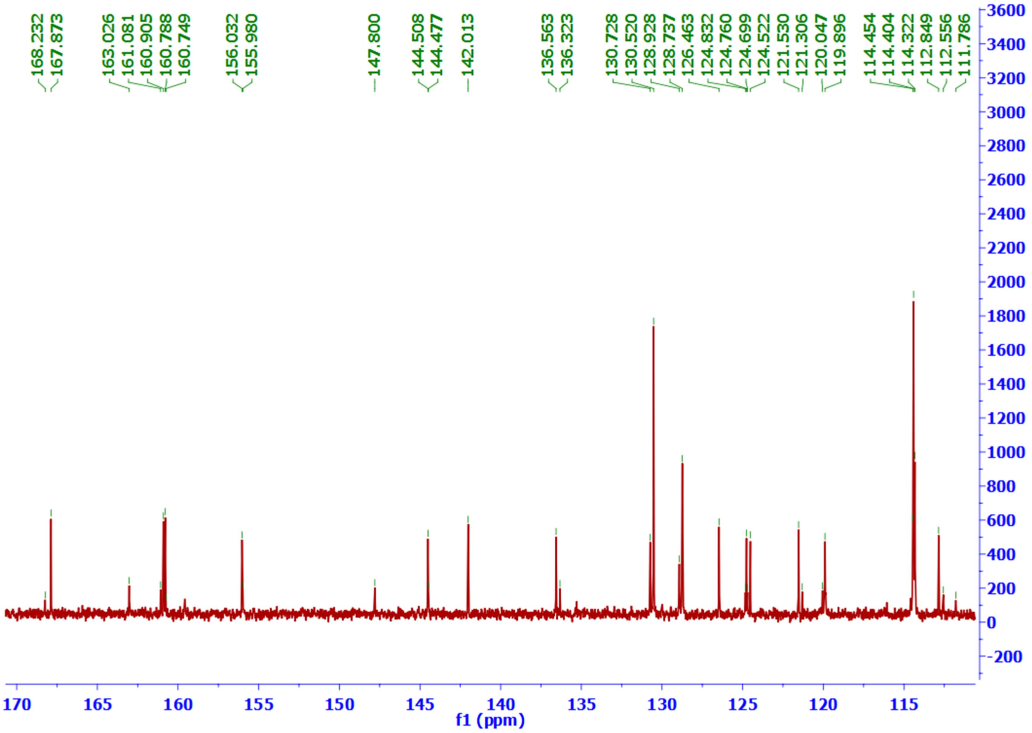

Figure 22. ^{13}C (100 MHz) NMR spectrum of **10c** in DMSO-d_6

2-(5,6-Dichloro-2-(4-methoxyphenyl)-1*H*-benzo[*d*]imidazol-1-yl)-*N*'-(4-methoxybenzylidene)acetohydrazide (10d)

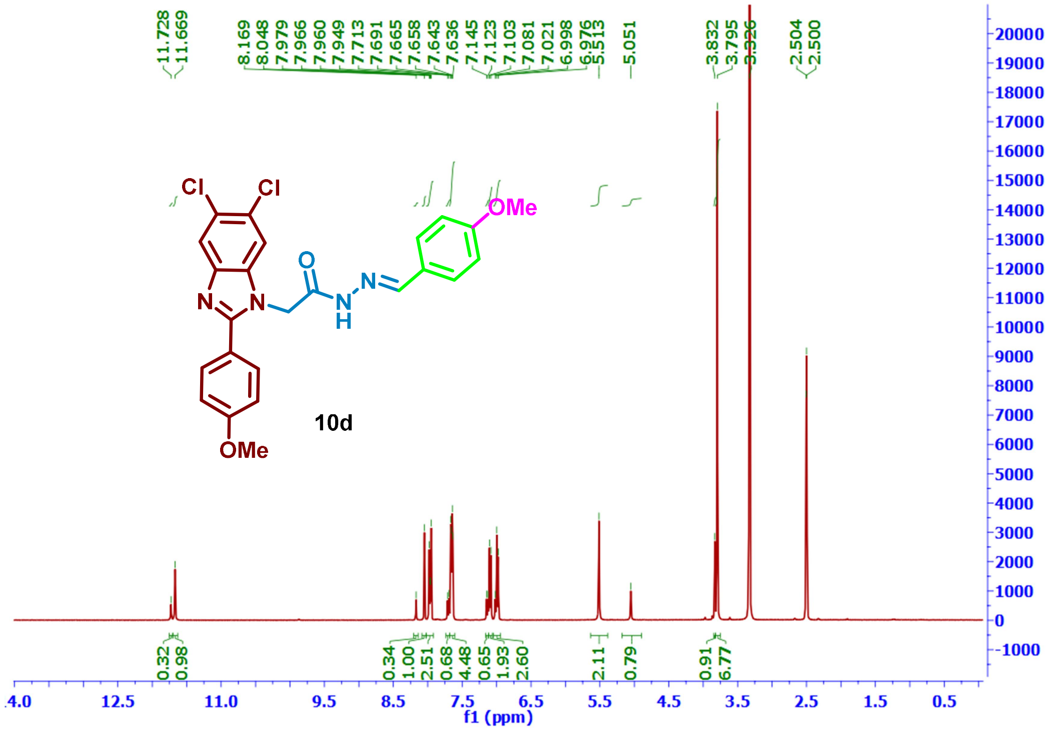

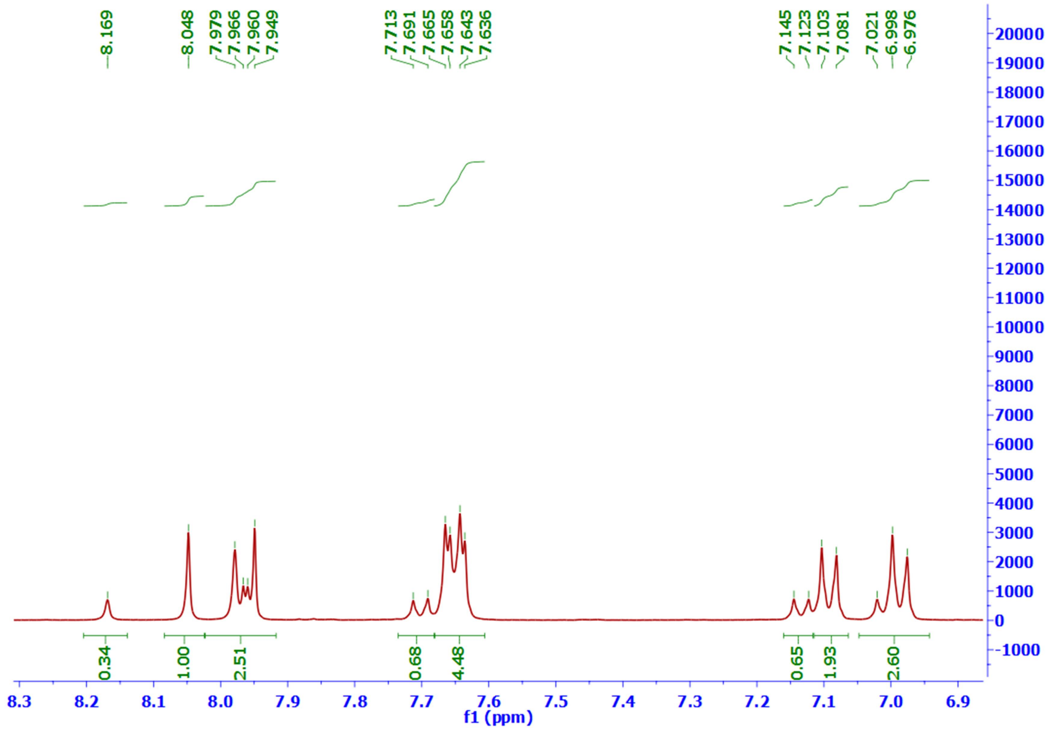

Figure 23. ^{1}H (400 MHz) NMR spectrum of **10d** in DMSO-d_6

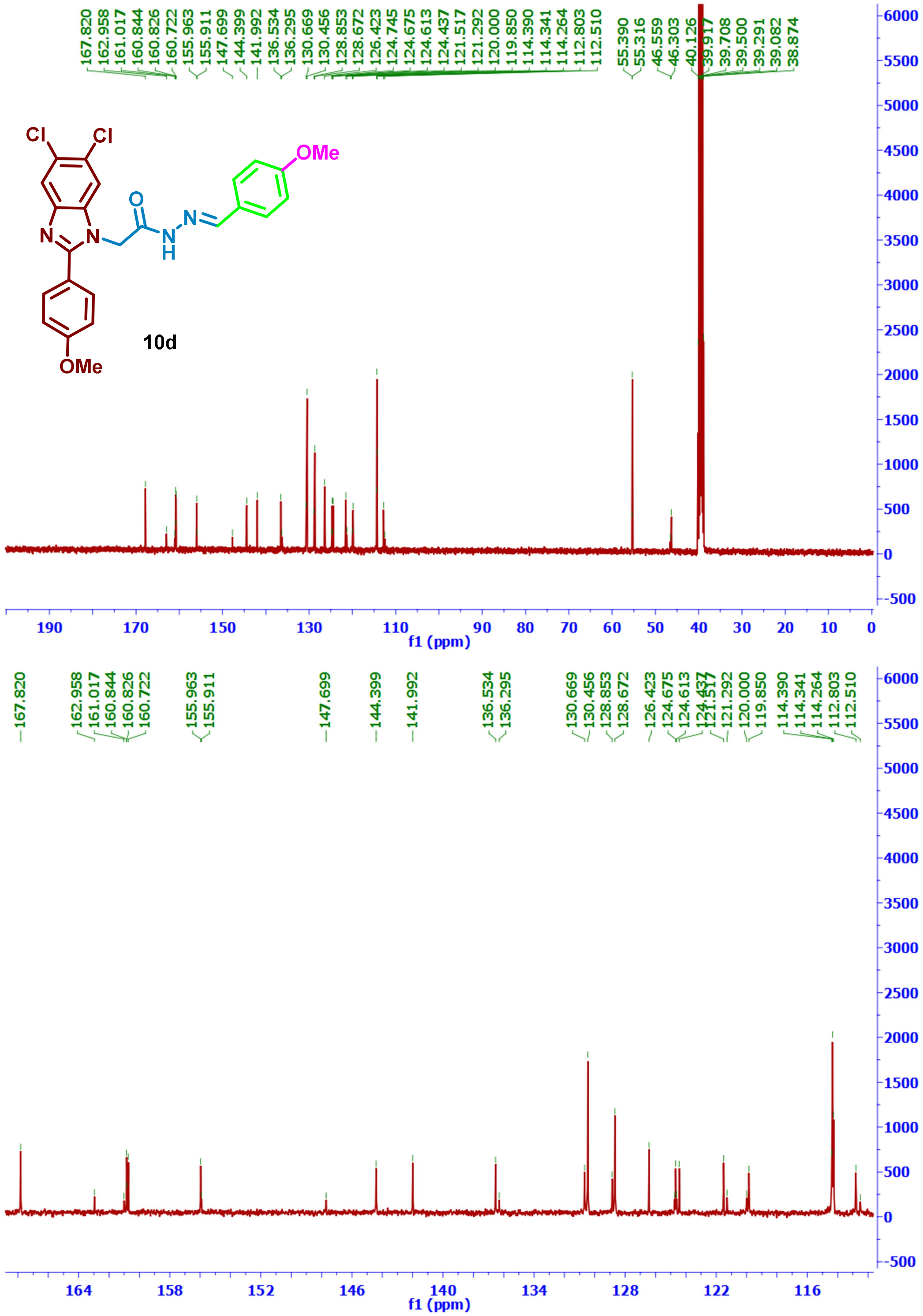

Figure 24. ^{13}C (100 MHz) NMR spectrum of **10d** in DMSO-d_6

2-(3-((2-(2-(5,6-Dichloro-2-(4-methoxyphenyl)-1*H*-benzo[*d*]imidazol-1-yl)acetyl)hydrazono)methyl)phenoxy)acetic acid (10e)

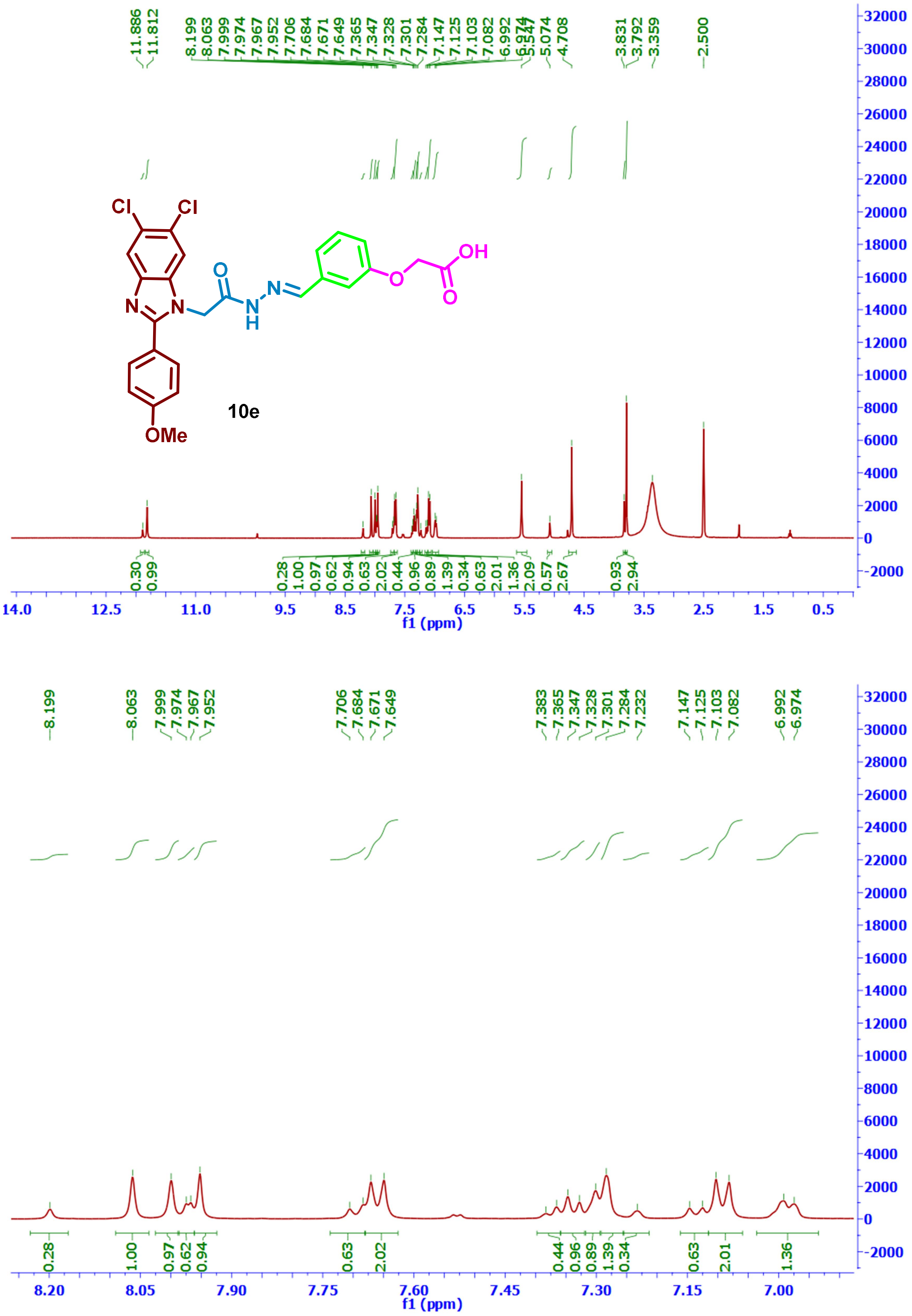

Figure 25. ^{1}H (400 MHz) NMR spectrum of **10e** in DMSO-d_6

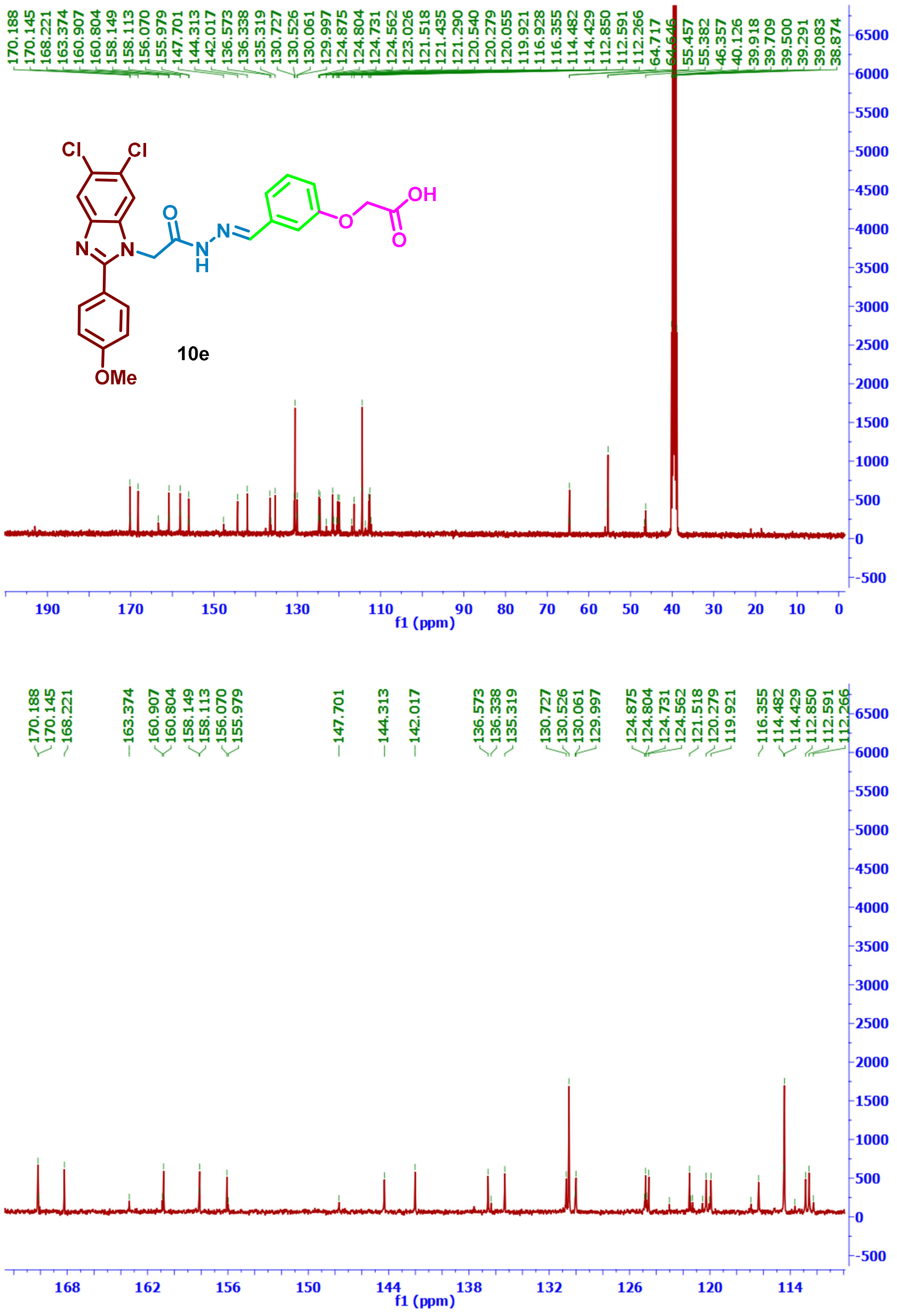

Figure 26. ^{13}C (100 MHz) NMR spectrum of **10e** in DMSO-d_6

2-(4-((2-(2-(5,6-Dichloro-2-(4-methoxyphenyl)-1*H*-benzo[*d*]imidazol-1-yl)acetyl)hydrazono)methyl) phenoxy)acetic acid (10f)

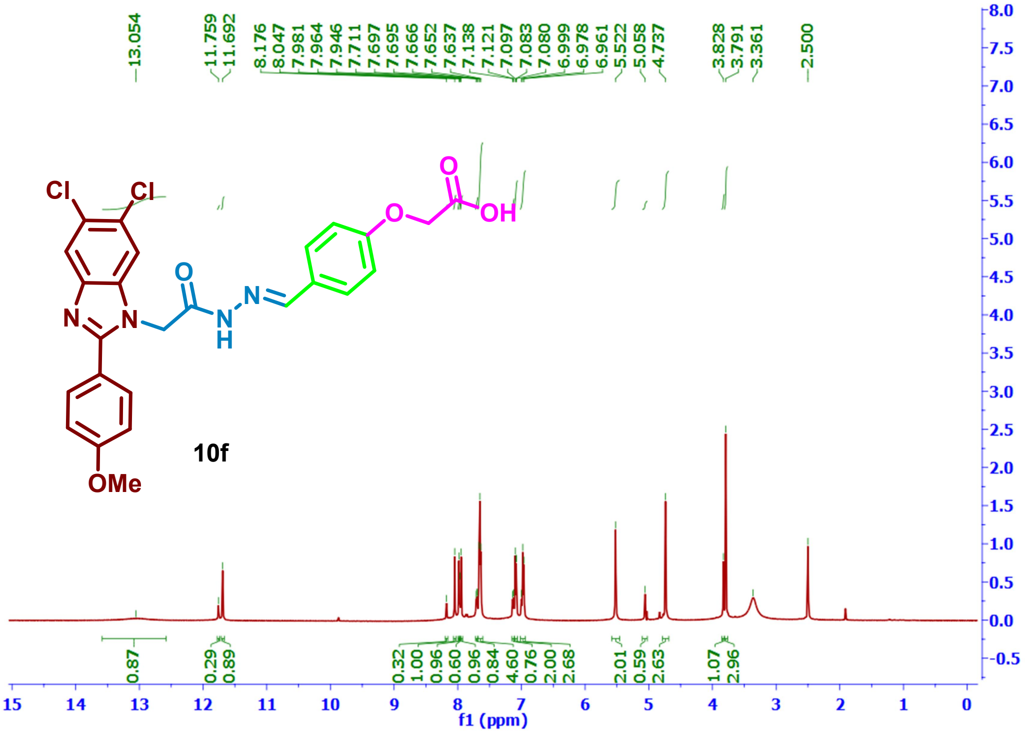

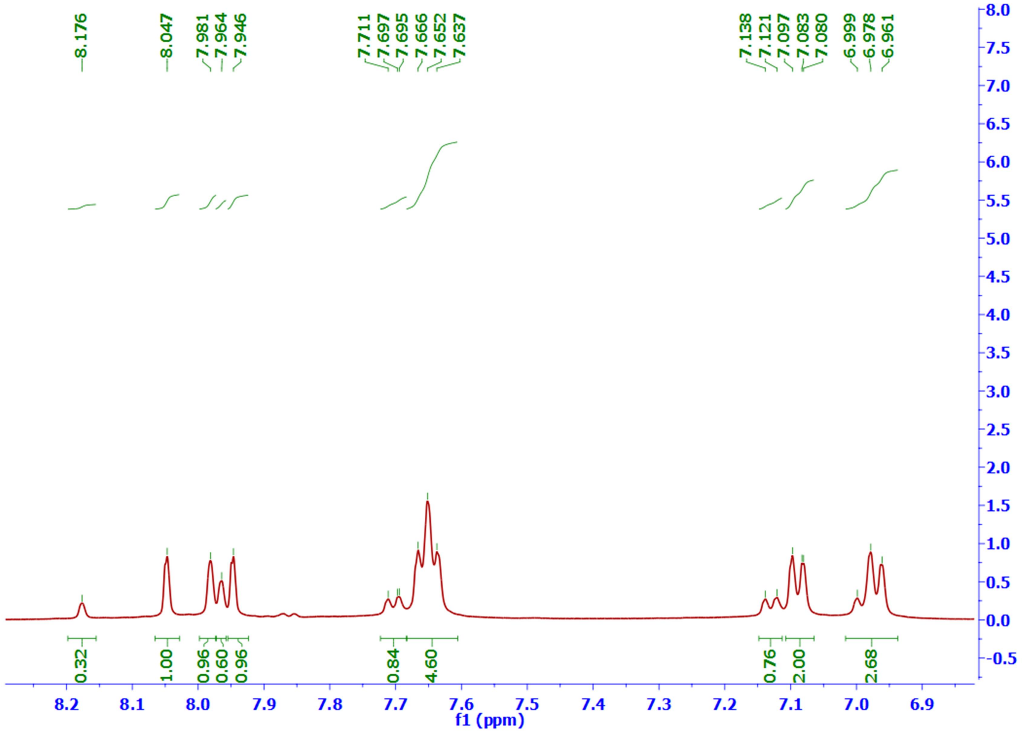

Figure 27. ^{1}H (500 MHz) NMR spectrum of **10f** in DMSO-d_6

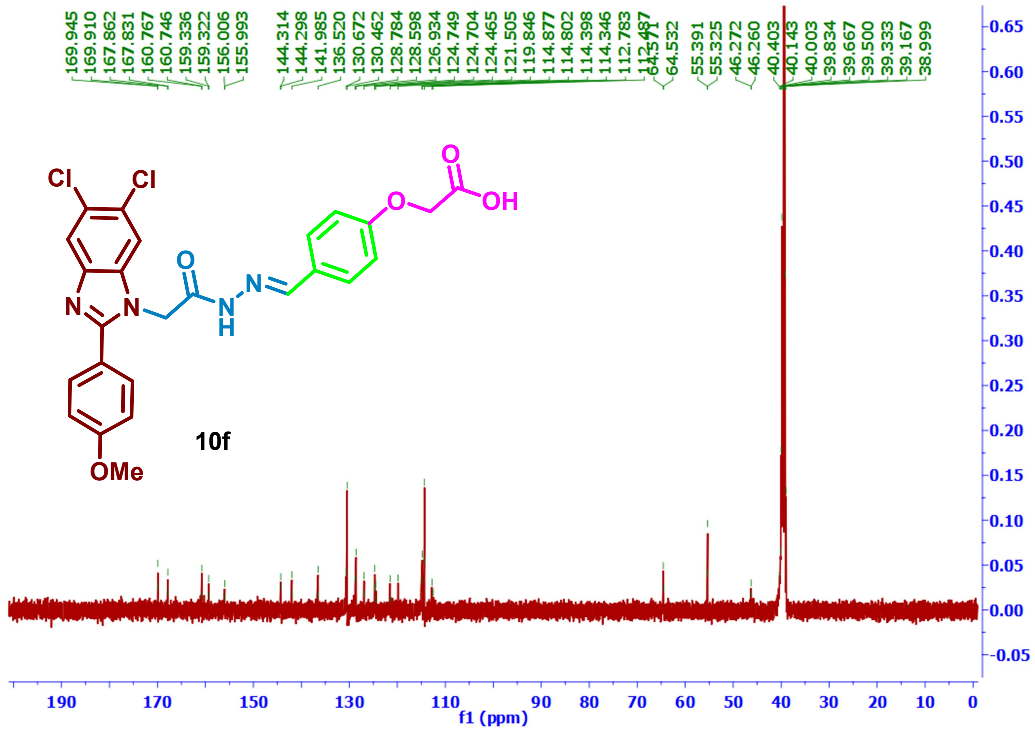

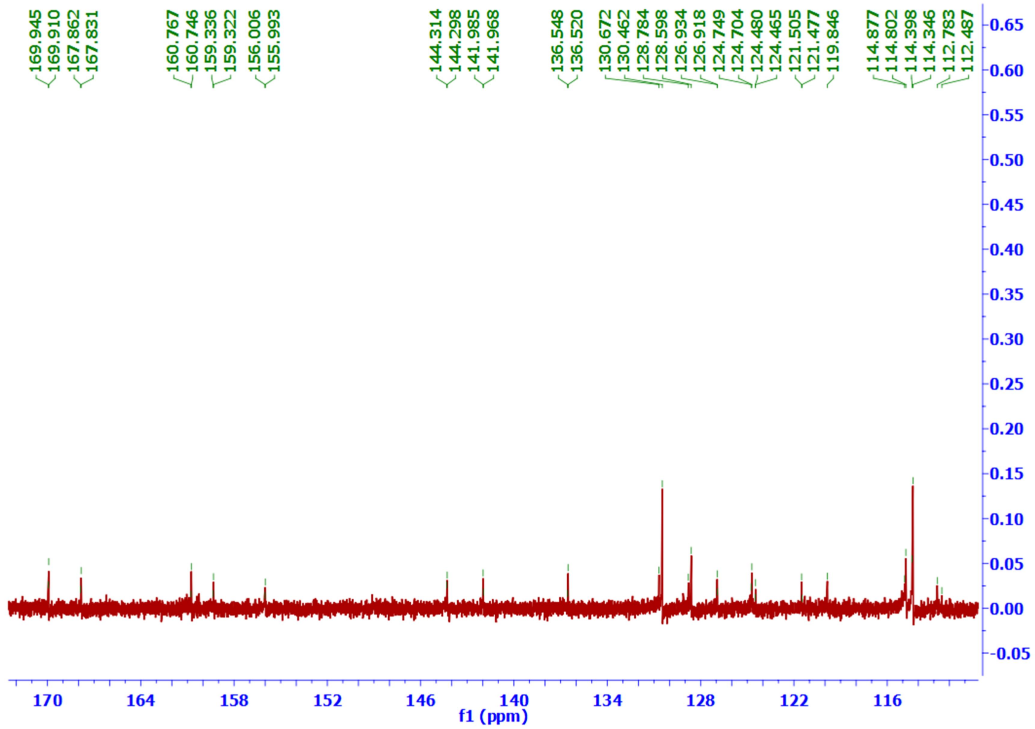

Figure 28. ^{13}C (125 MHz) NMR spectrum of **10f** in DMSO-d_6

Methyl-2-(3-((2-(2-(5,6-dichloro-2-(4-methoxyphenyl)-1*H*-benzo[*d*]imidazol-1-yl)acetyl)hydrazono)methyl)phenoxy)acetate (10g)

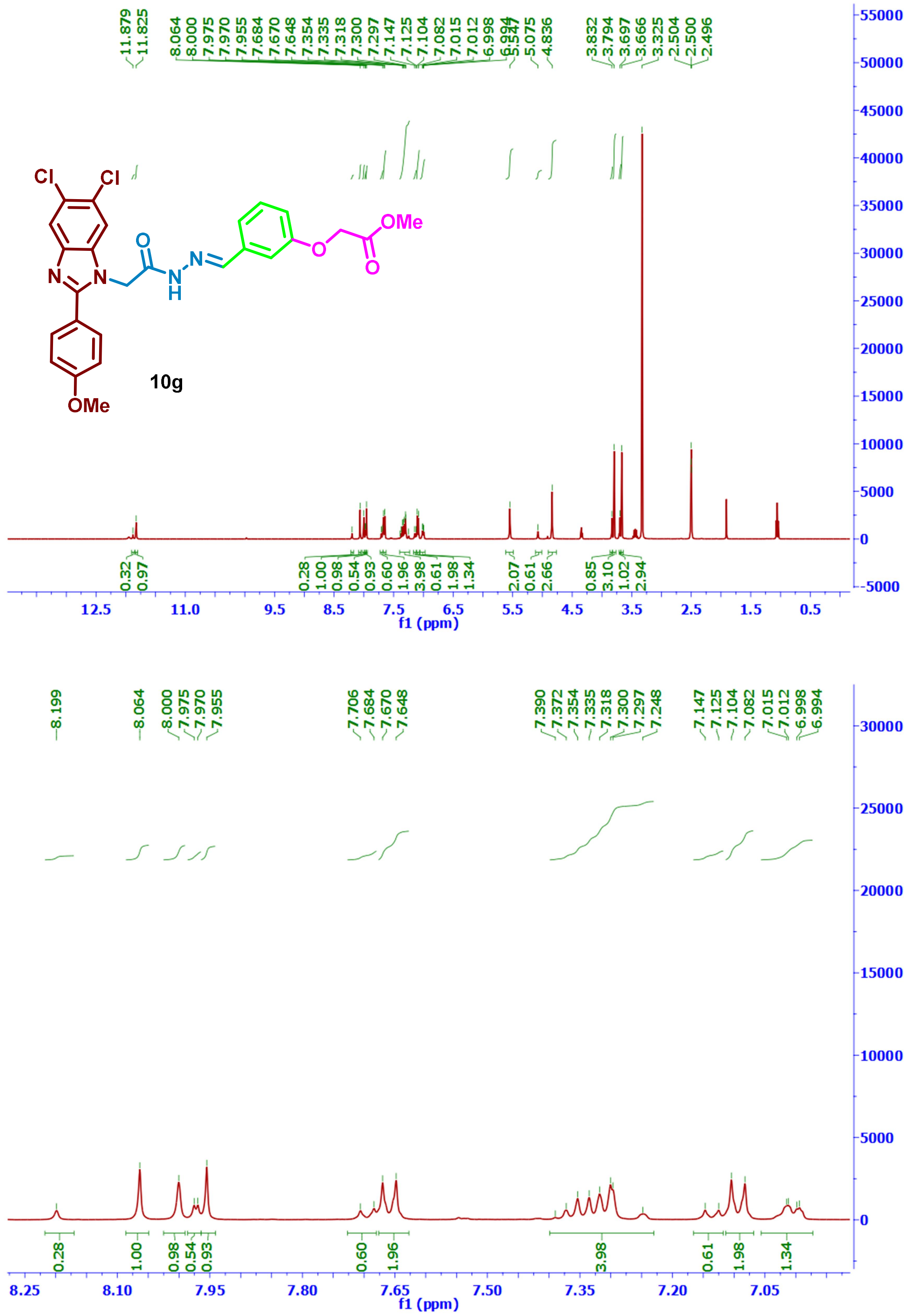

Figure 29. ^{1}H (400 MHz) NMR spectrum of **10g** in DMSO-d_6

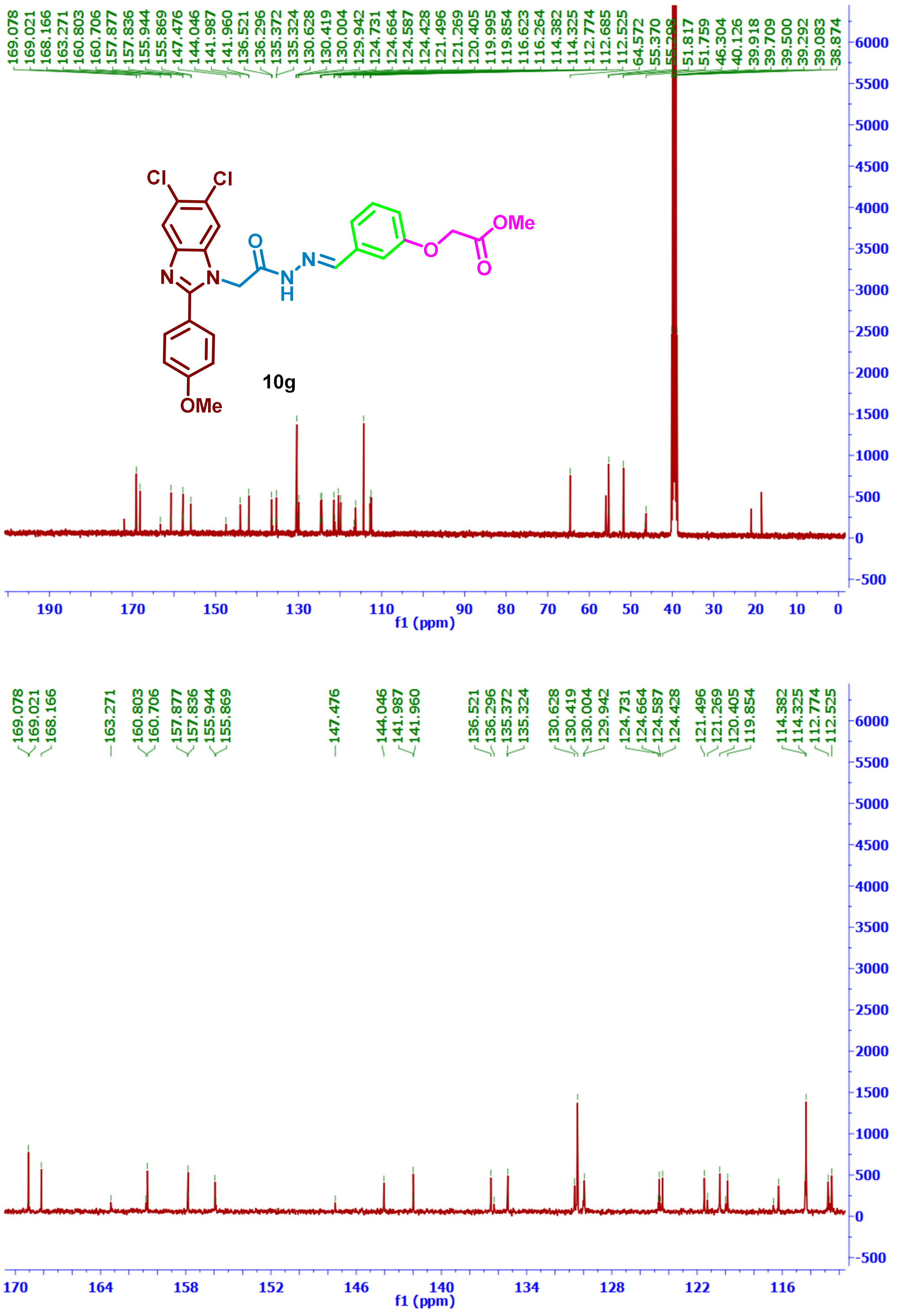

Figure 30. ^{13}C (100 MHz) NMR spectrum of **10g** in DMSO-d_6

Methyl-2-(4-((2-(2-(5,6-dichloro-2-(4-methoxyphenyl)-1*H*-benzo[*d*]imidazol-1-yl)acetyl)hydrazono) methyl)phenoxy)acetate (10h)

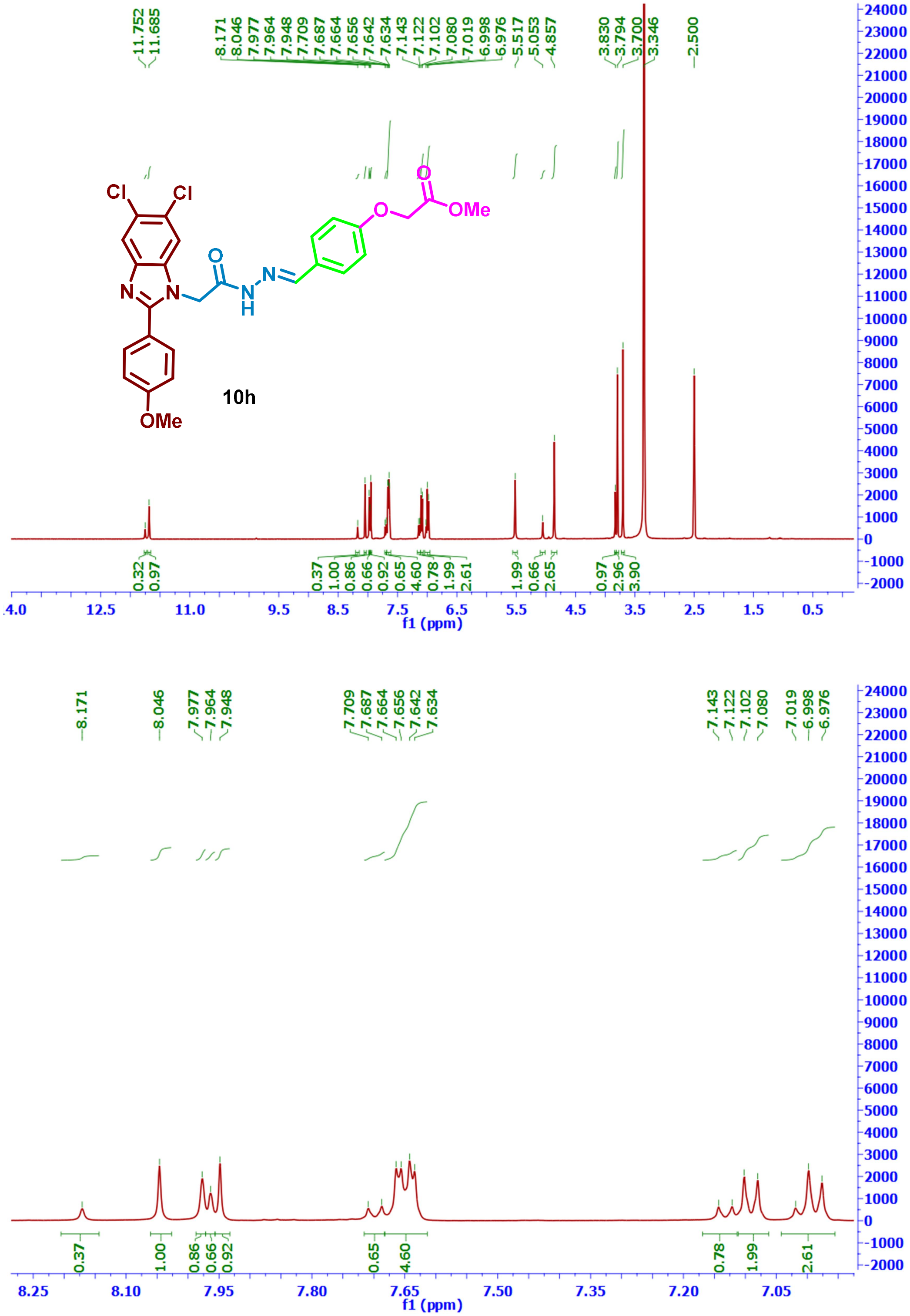

Figure 31. ^{1}H (400 MHz) NMR spectrum of **10h** in DMSO-d_6

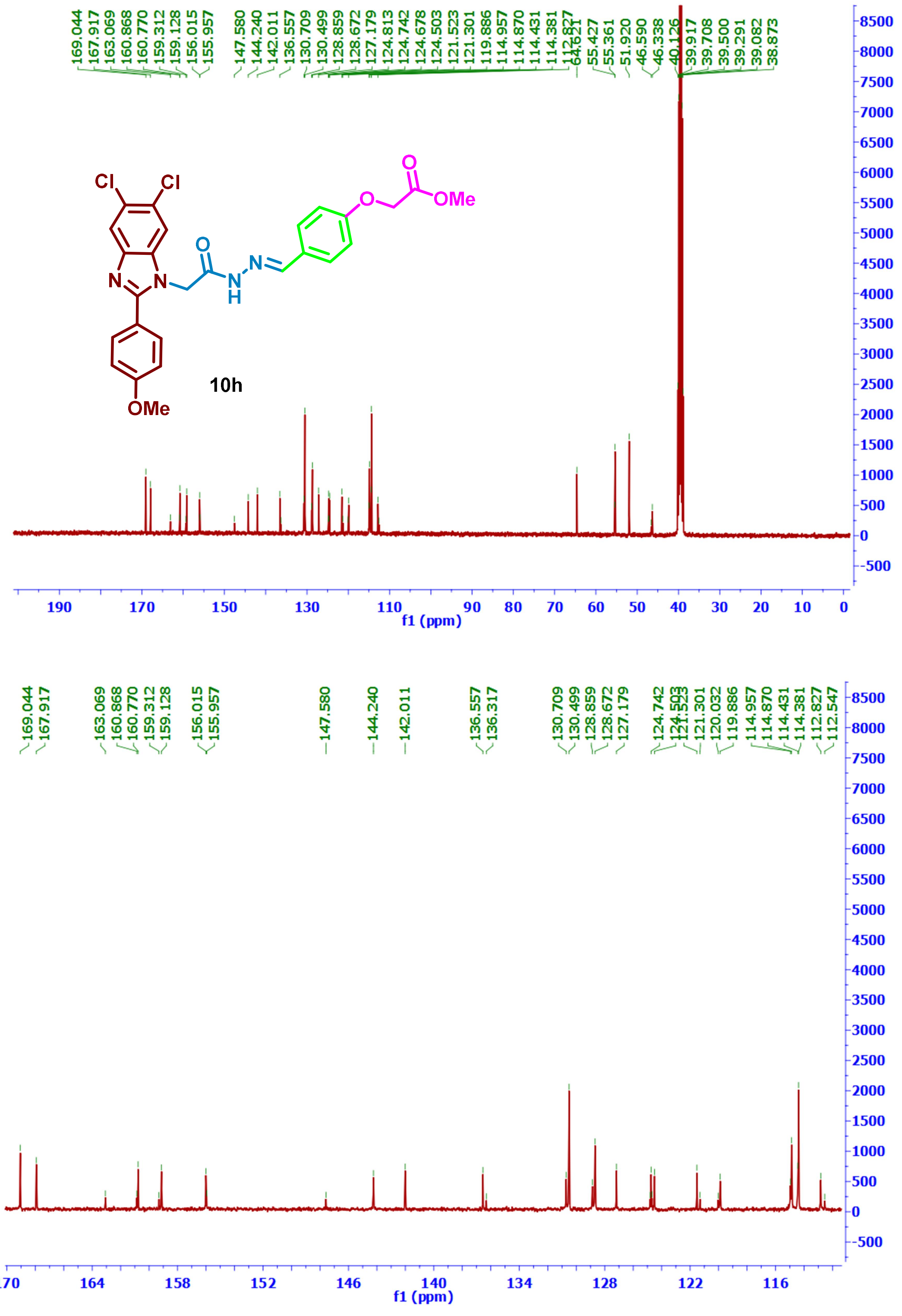

Figure 32. ^{13}C (100 MHz) NMR spectrum of **10h** in DMSO-d_6

3-(4-((2-(2-(5,6-Dichloro-2-(4-methoxyphenyl)-1*H*-benzo[*d*]imidazol-1-yl)acetyl)hydrazono)methyl)phenoxy)propanoic acid (10i)

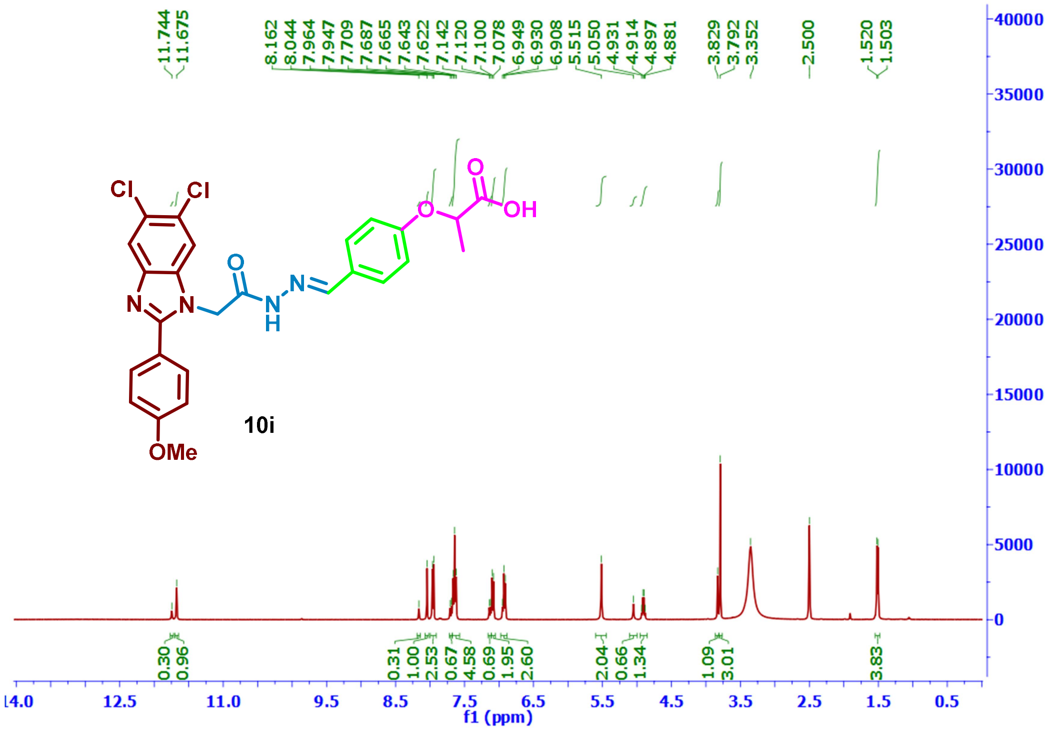

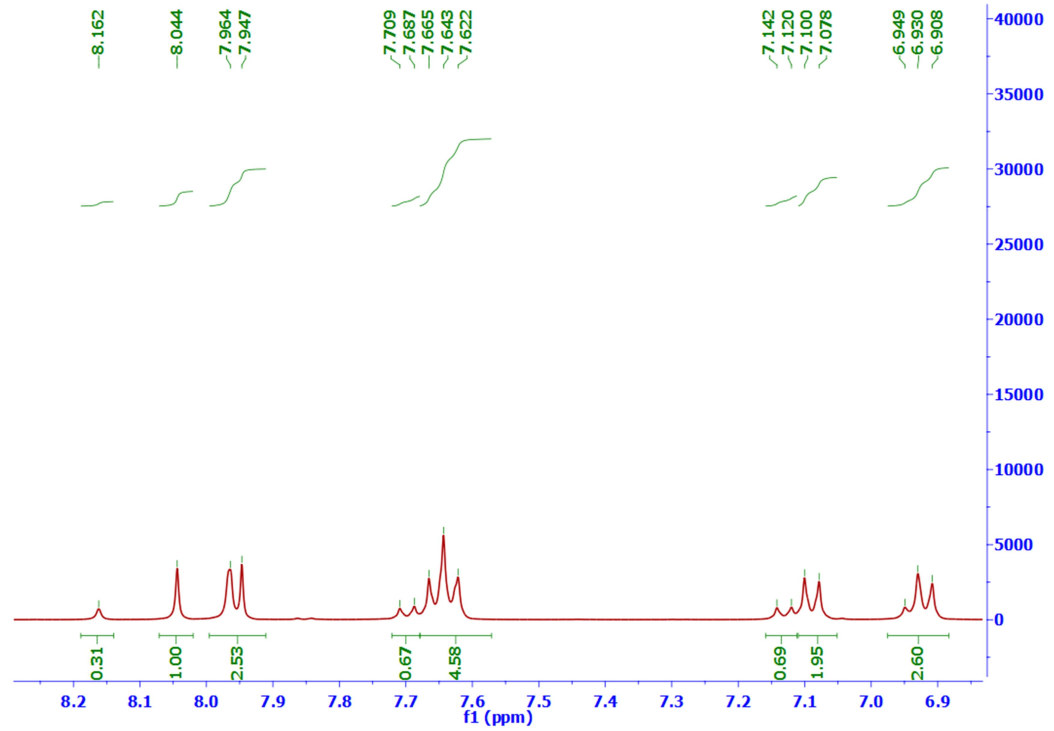

Figure 33. ^{1}H (400 MHz) NMR spectrum of **10i** in DMSO-d_6

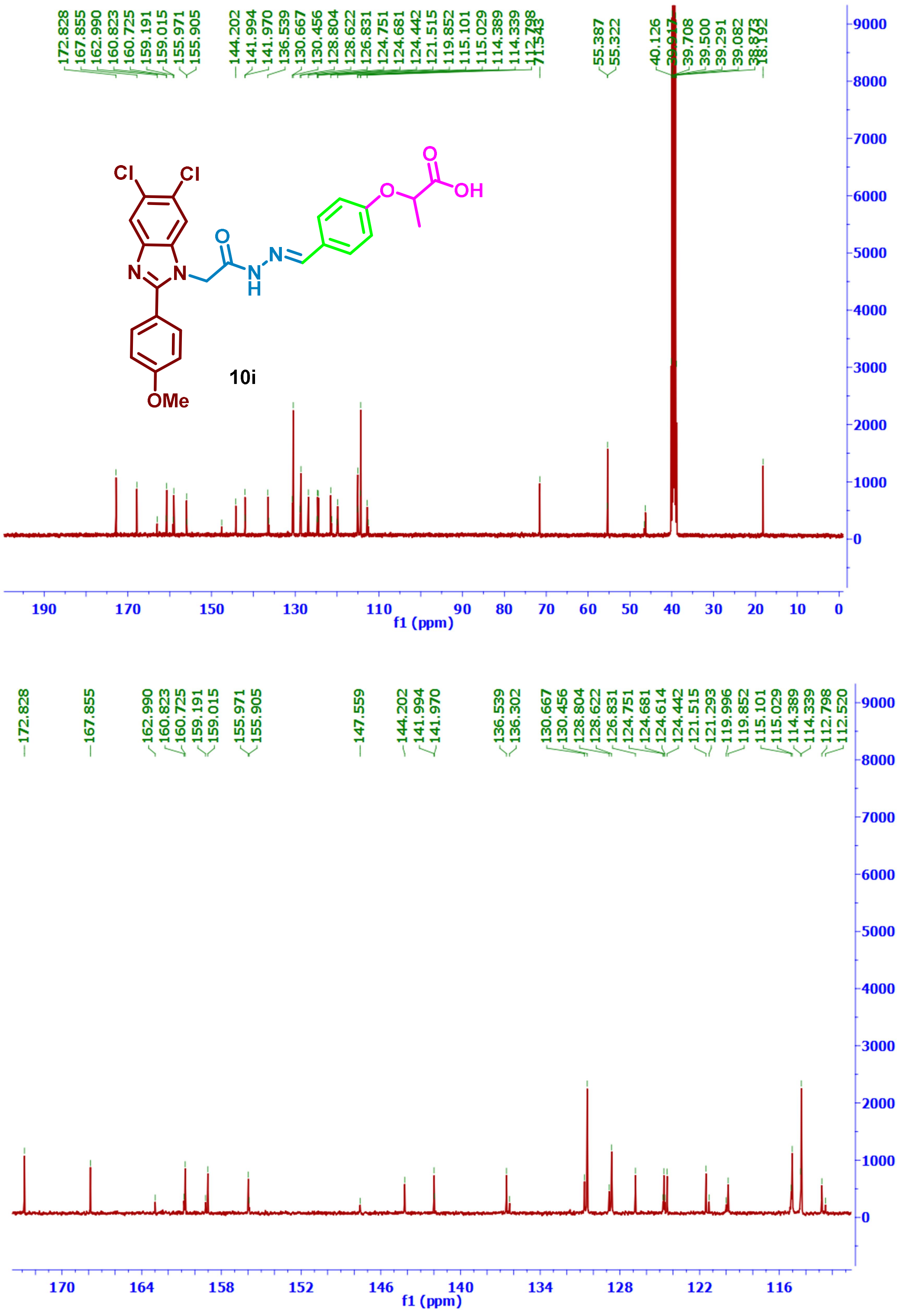

Figure 34. ^{13}C (100 MHz) NMR spectrum of **10i** in DMSO-d_6

Ethyl-2-(4-((2-(2-(5,6-dichloro-2-(4-methoxyphenyl)-1*H*-benzo[*d*]imidazol-1-yl)acetyl) hydrazono)methyl)phenoxy)propanoate (10j)

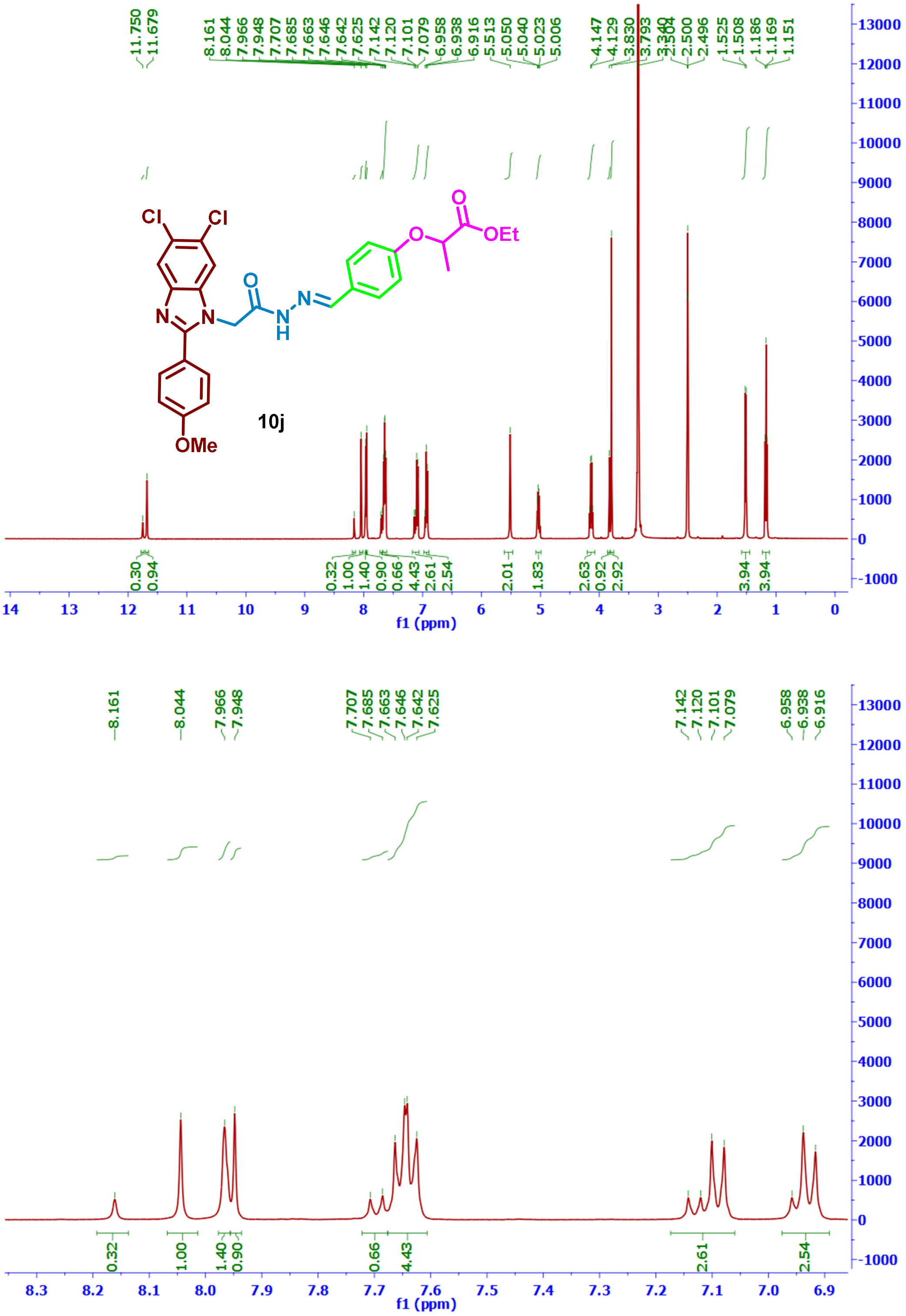

Figure 35. ^{1}H (400 MHz) NMR spectrum of **10j** in DMSO-d_6

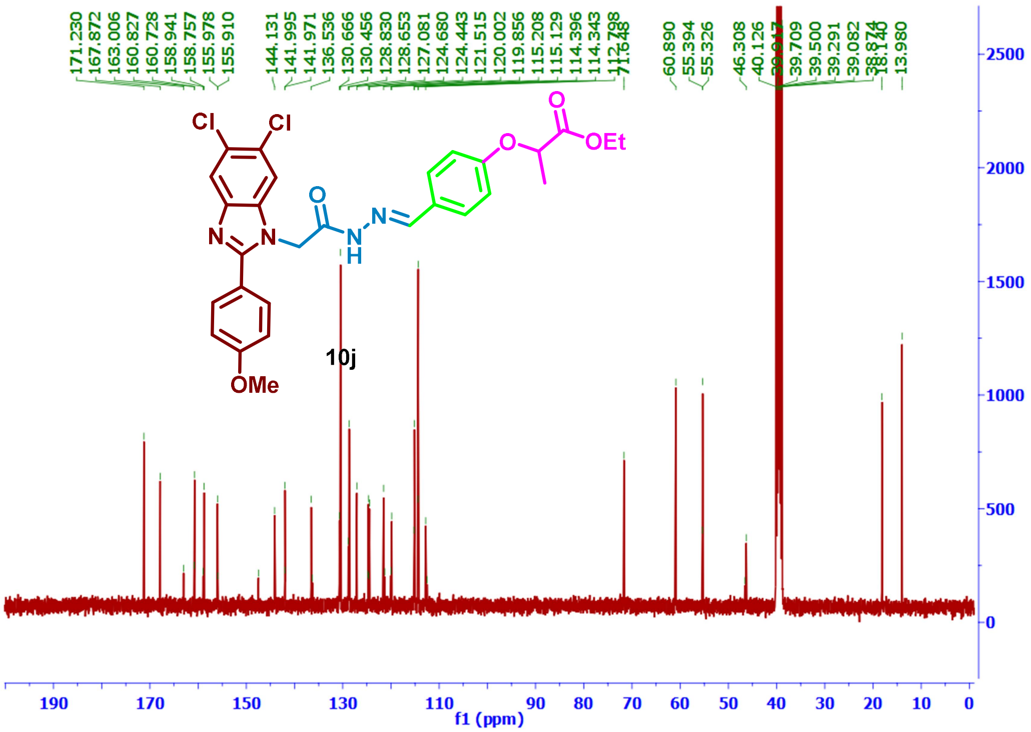

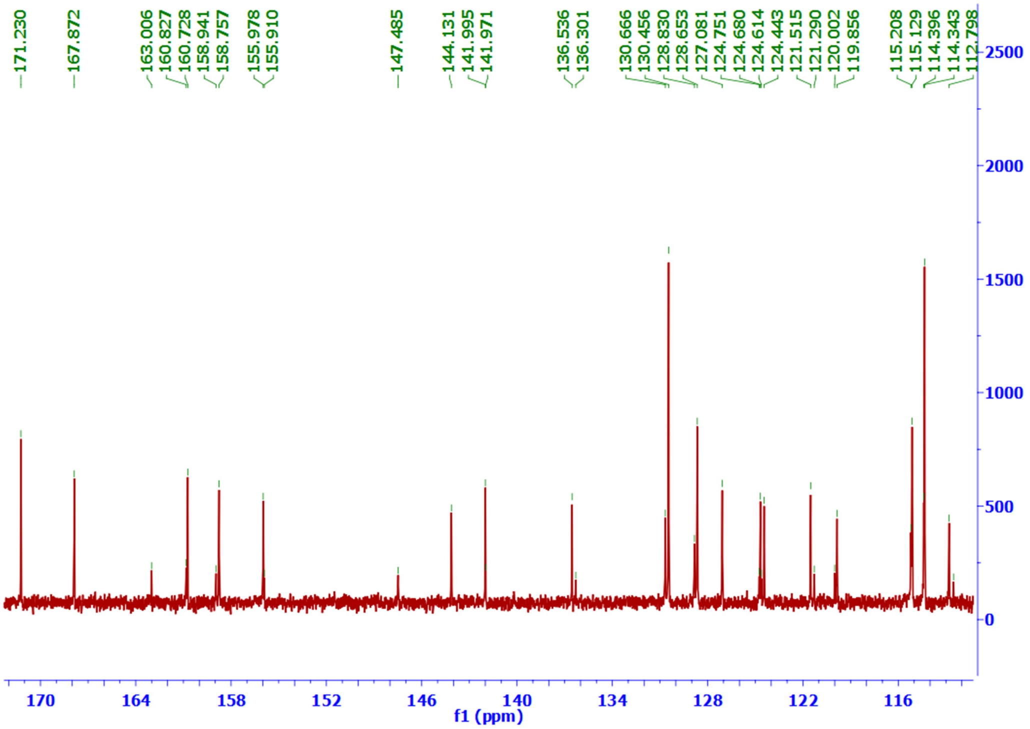

Figure 36. ^{13}C (100 MHz) NMR spectrum of **10j** in DMSO-d_6

2-(5,6-Dichloro-2-(4-methoxyphenyl)-1*H*-benzo[*d*]imidazol-1-yl)-*N'*-(3-hydroxy-4-methoxy benzylidene)acetohydrazide (10k)

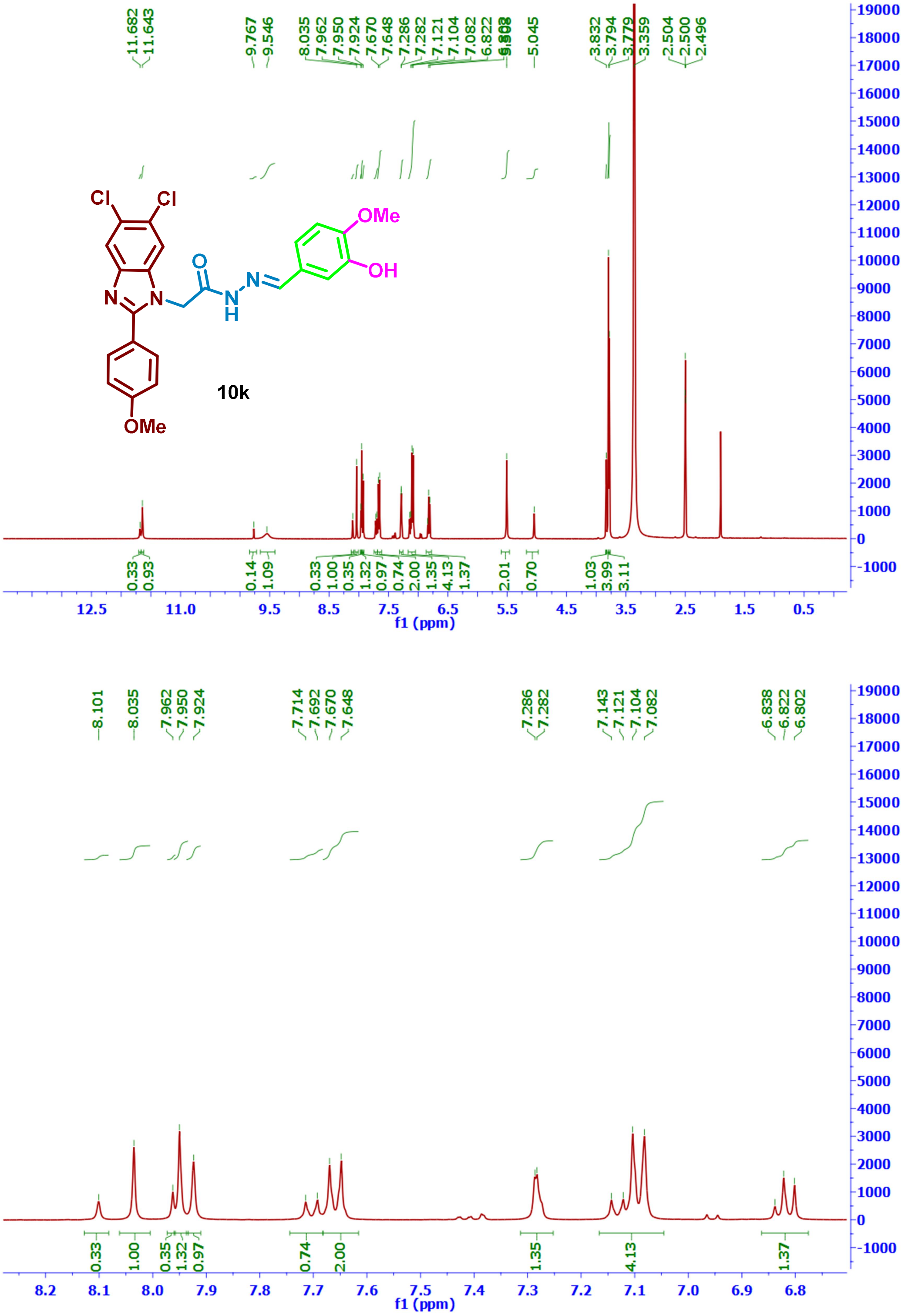

Figure 37. ^{1}H (400 MHz) NMR spectrum of **10k** in DMSO-d_6

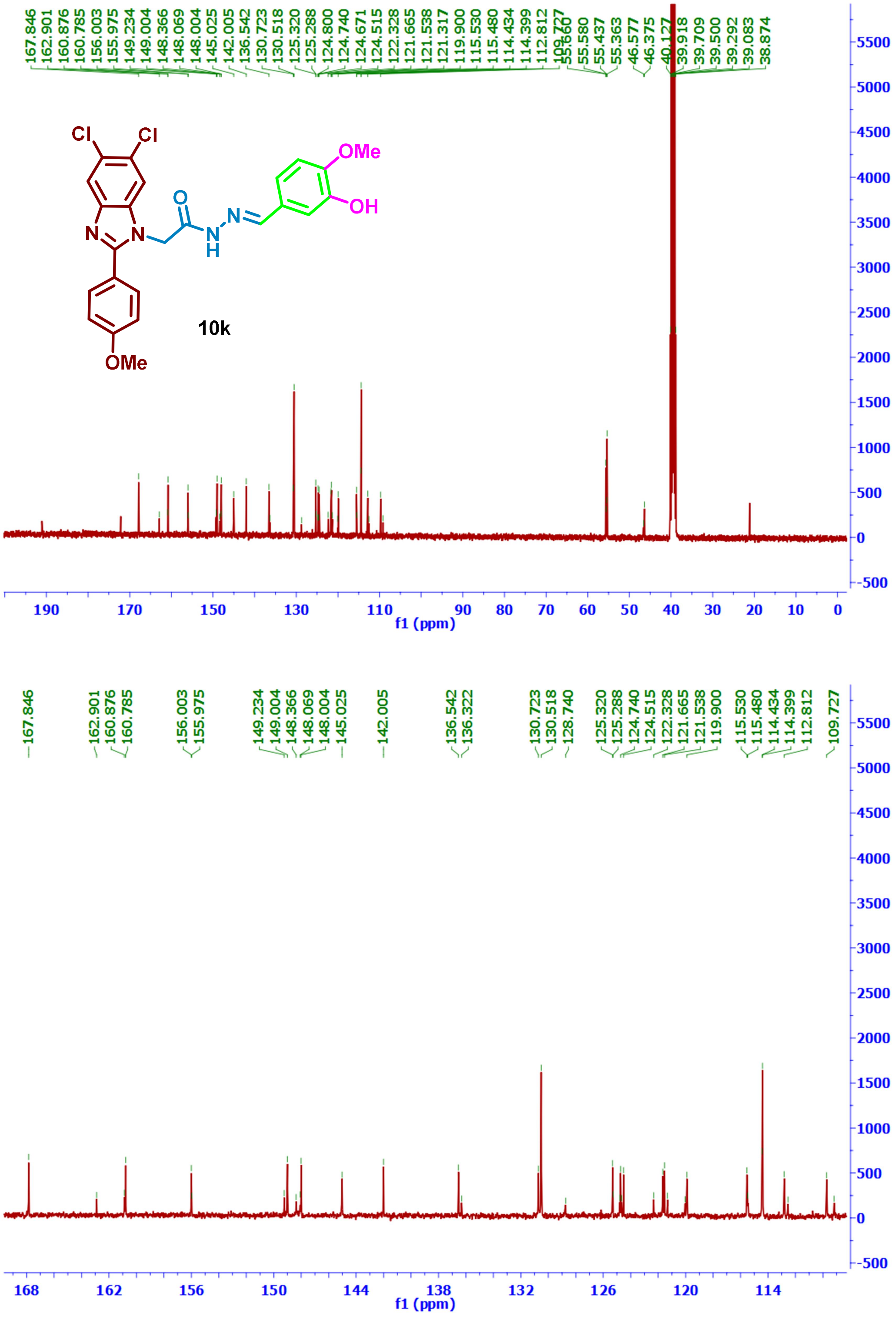

Figure 38. ^{13}C (100 MHz) NMR spectrum of **10k** in DMSO-d_6

2-(5,6-Dichloro-2-(4-methoxyphenyl)-1*H*-benzo[*d*]imidazol-1-yl)-*N'*-(4-hydroxy-3-methoxy benzylidene)acetohydrazide (10l)

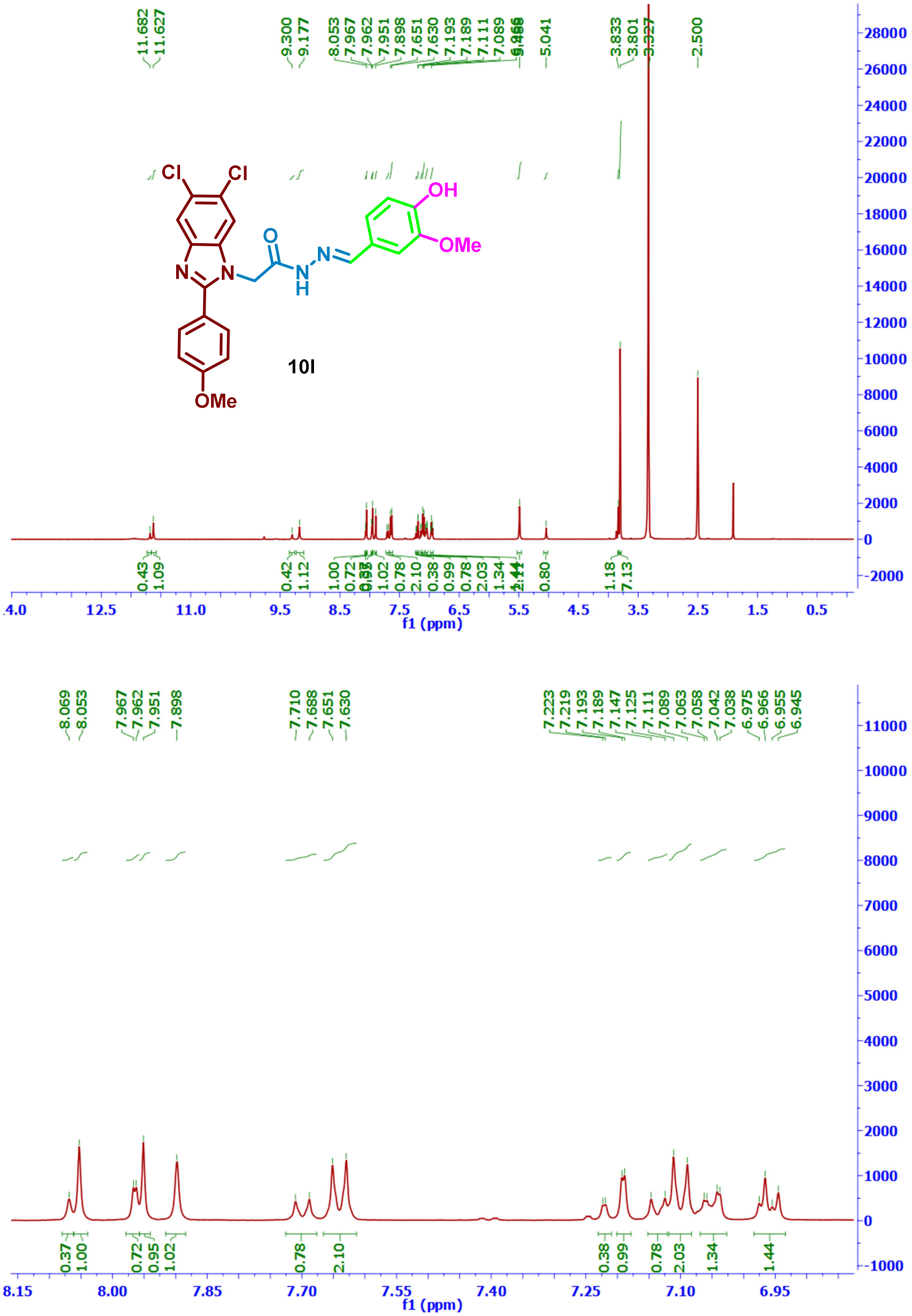

Figure 39. ^{1}H (400 MHz) NMR spectrum of **10l** in DMSO-d_6

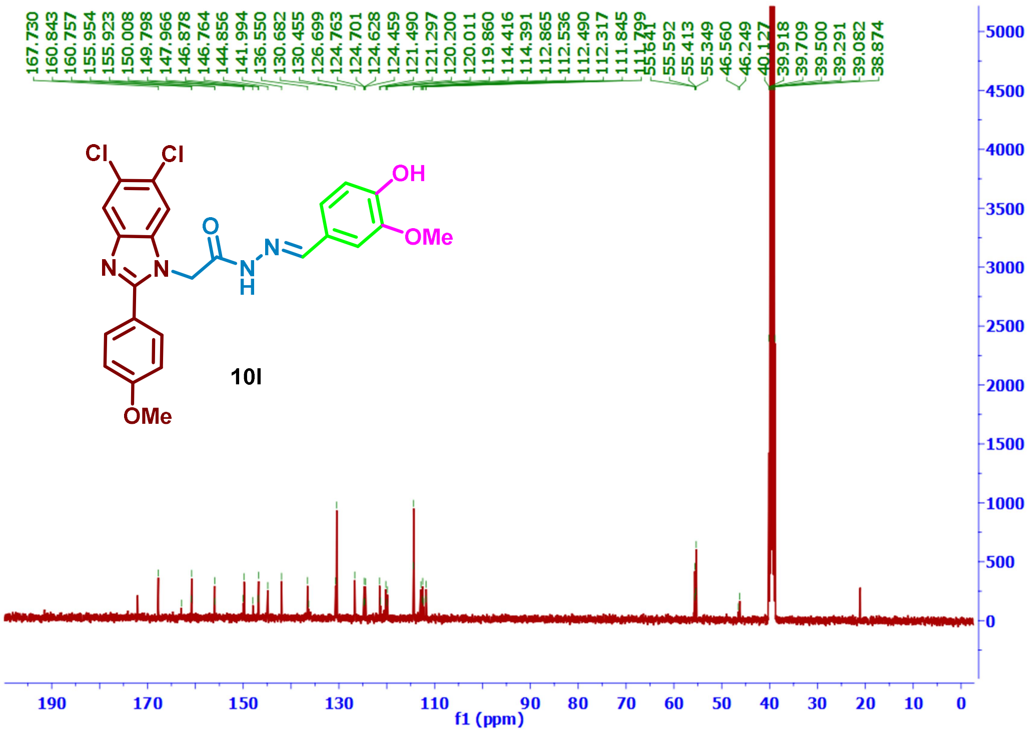

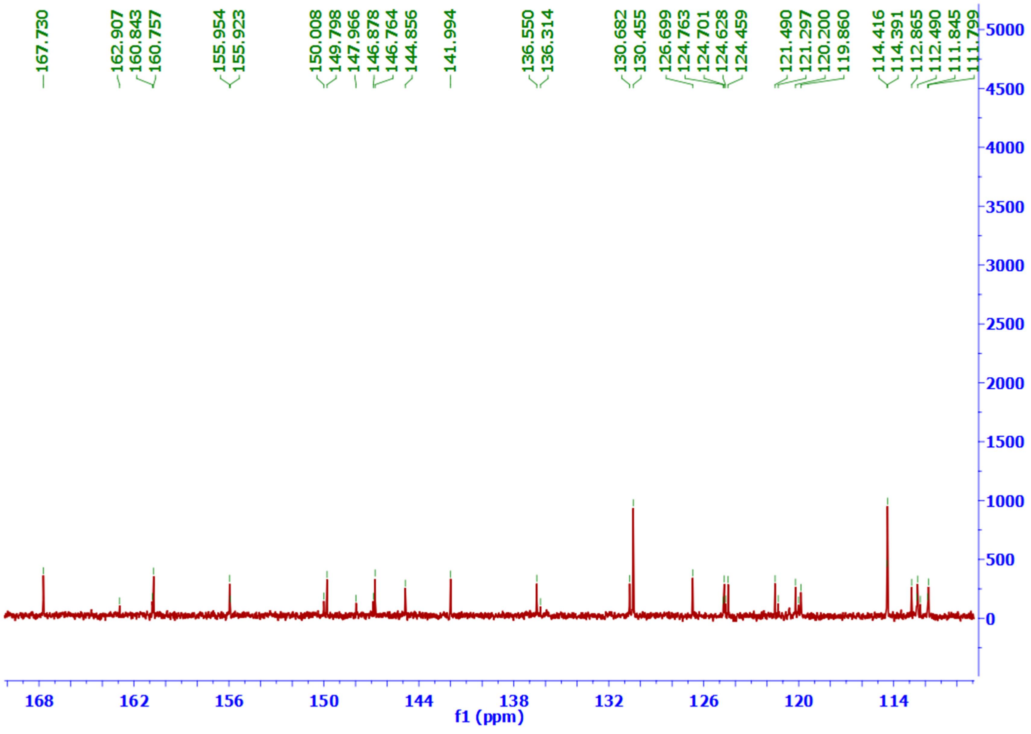

Figure 40. ^{13}C (100 MHz) NMR spectrum of **10l** in DMSO-d_6

2-(4-((2-(2-(5,6-Dichloro-2-(4-methoxyphenyl)-1*H*-benzo[*d*]imidazol-1-yl)acetyl)hydrazono) methyl)-2-methoxyphenoxy)acetic acid (10m)

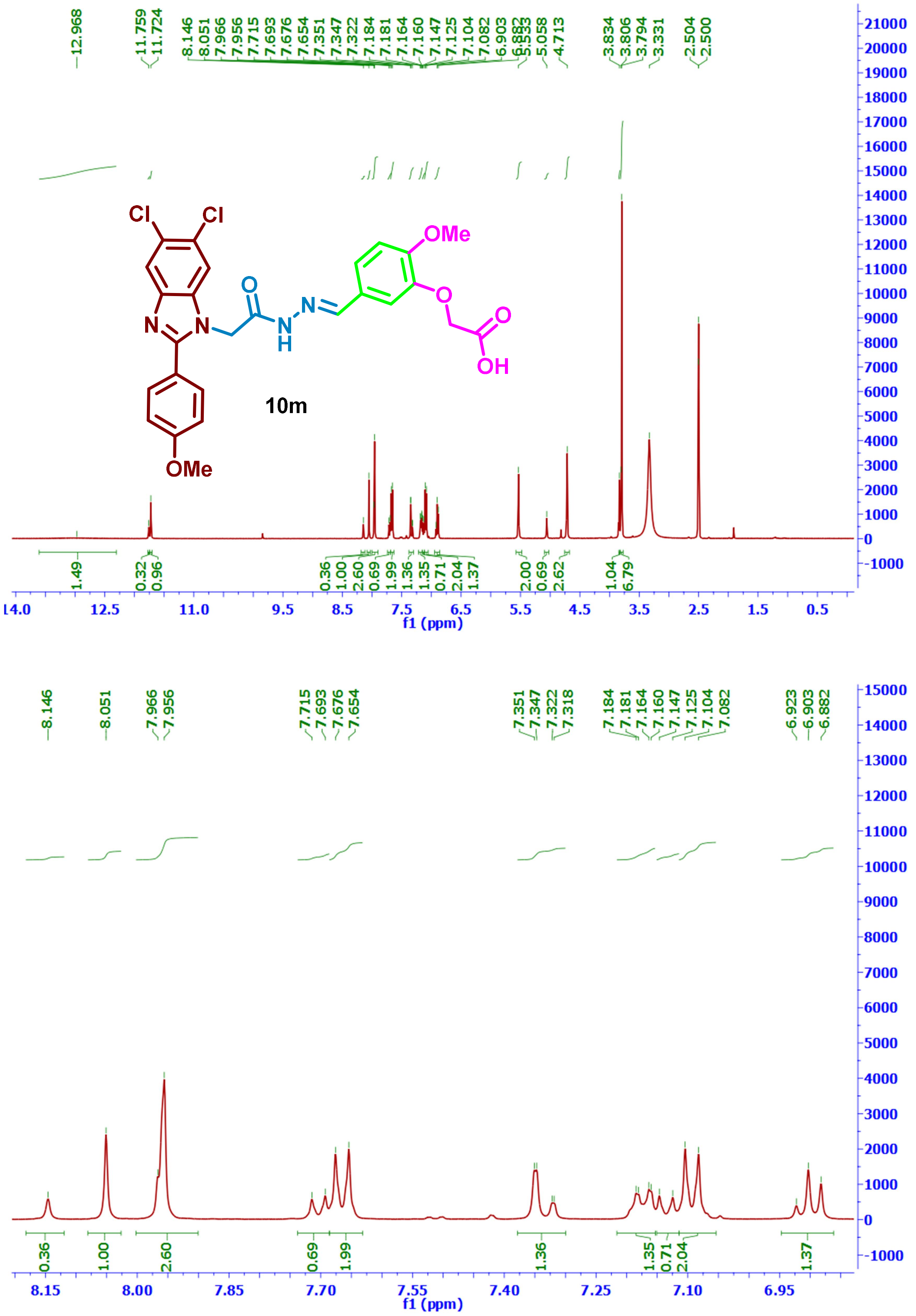

Figure 41. ^{1}H (400 MHz) NMR spectrum of **10m** in DMSO-d_6

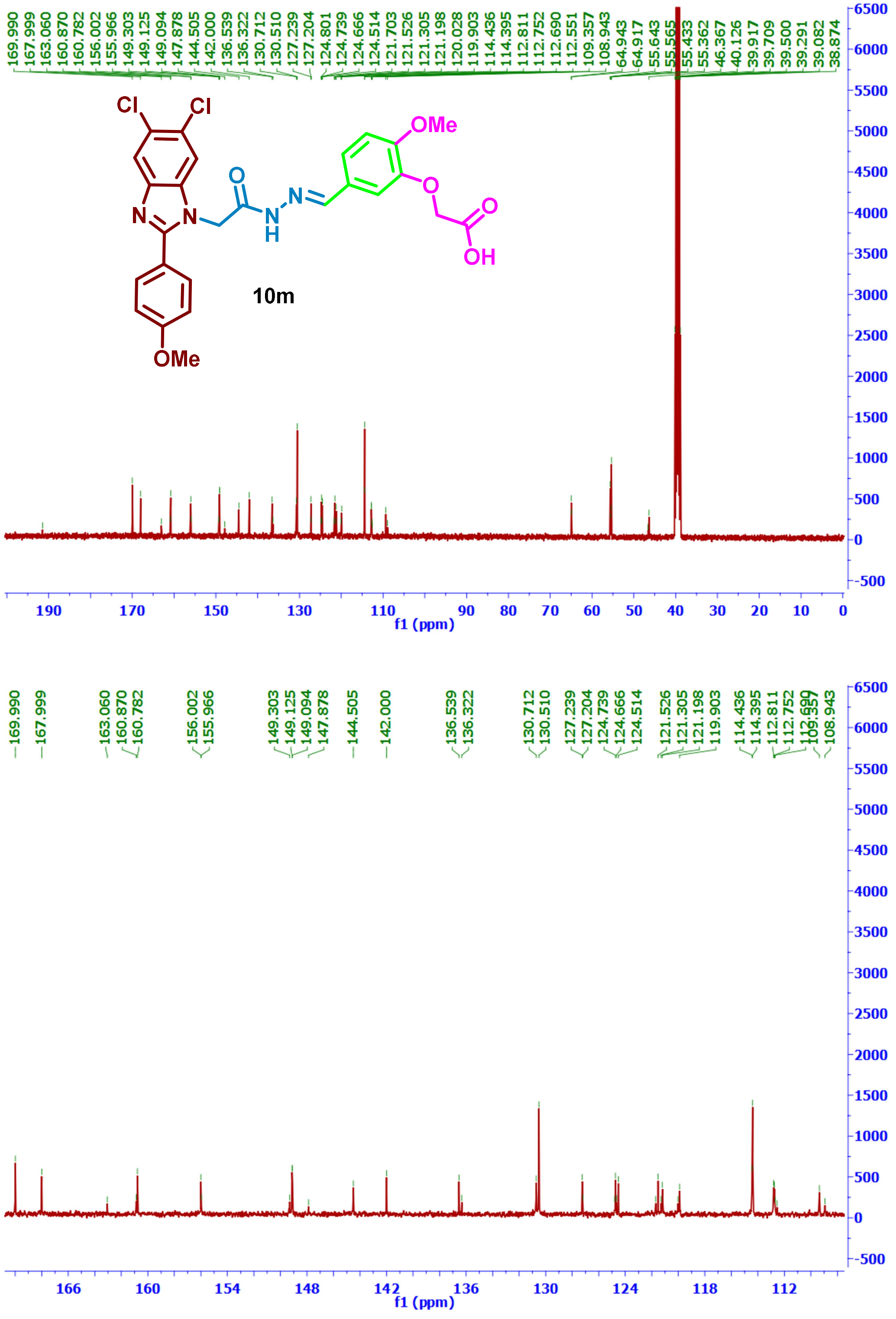

Figure 42. ^{13}C (100 MHz) NMR spectrum of **10m** in DMSO-d_6

2-(5-((2-(2-(5,6-Dichloro-2-(4-methoxyphenyl)-1*H*-benzo[*d*]imidazol-1-yl)acetyl)hydrazono)methyl)-2-methoxyphenoxy)acetic acid (10n)

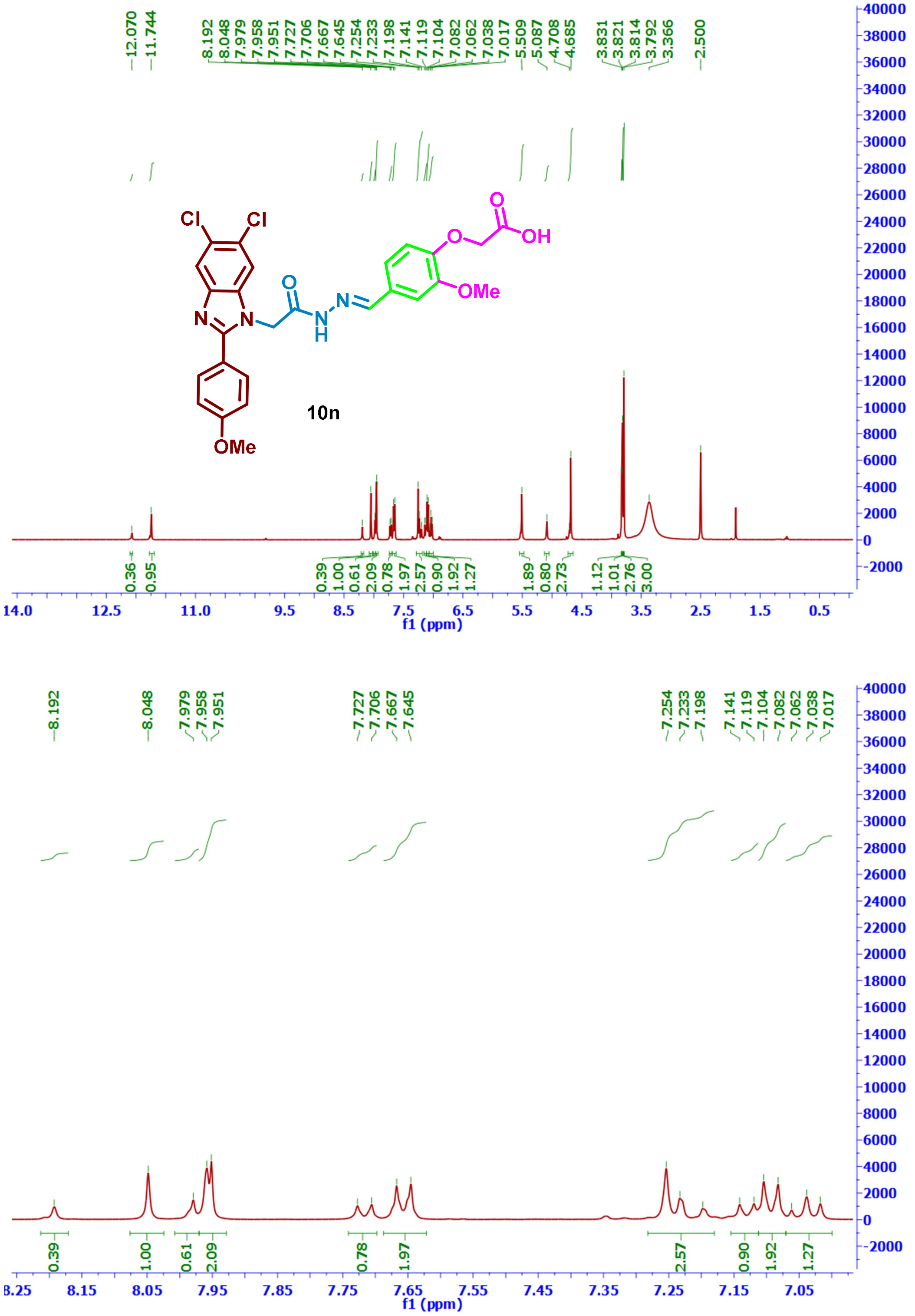

Figure 43. ^{1}H (400 MHz) NMR spectrum of **10n** in DMSO-d_6

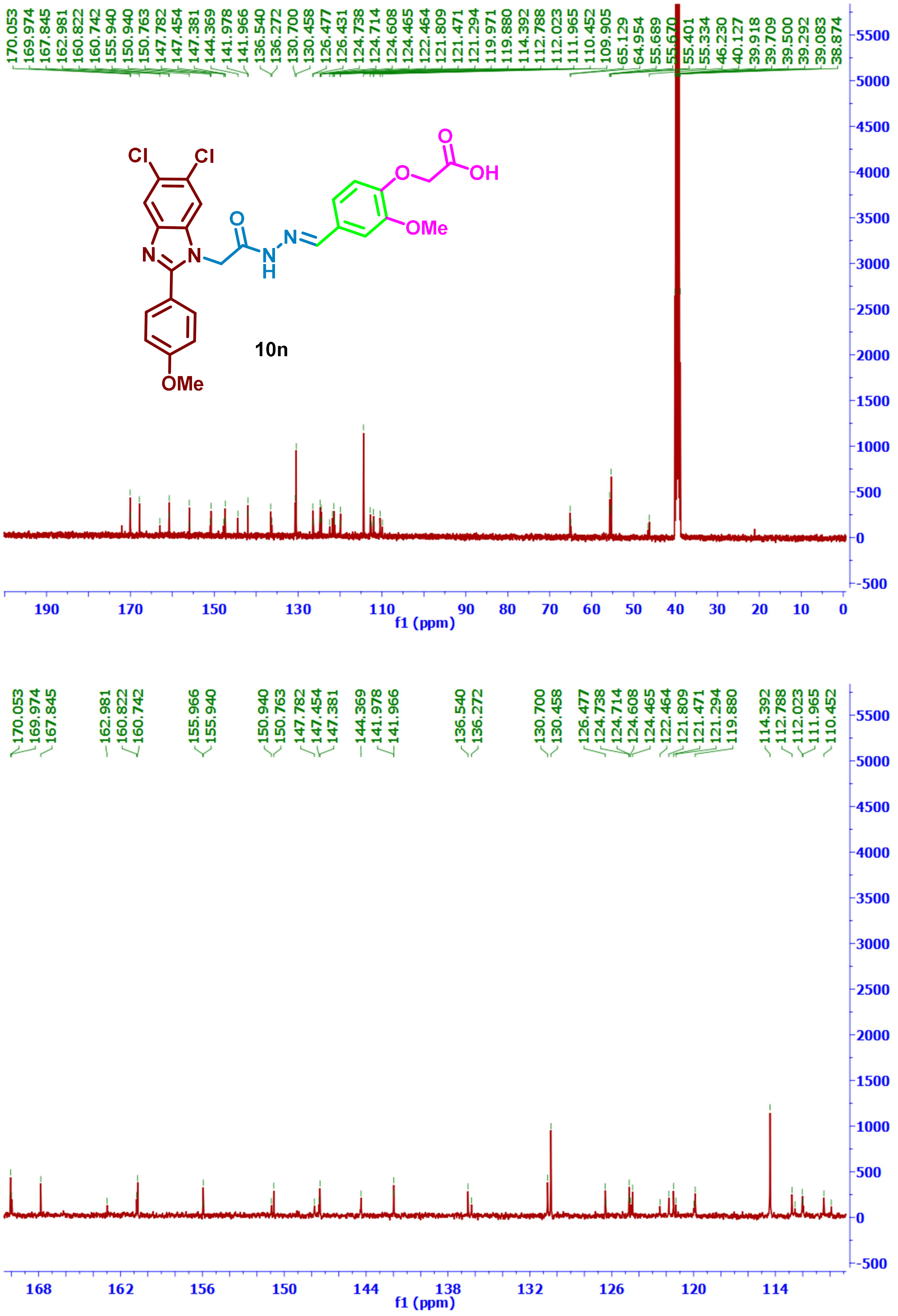

Figure 44. ^{13}C (100 MHz) NMR spectrum of **10n** in DMSO-d_6

Methyl-2-(5-((2-(2-(5,6-dichloro-2-(4-methoxyphenyl)-1*H*-benzo[*d*]imidazol-1-yl)acetyl)hydrazono) methyl)-2-methoxyphenoxy)acetate (10o)

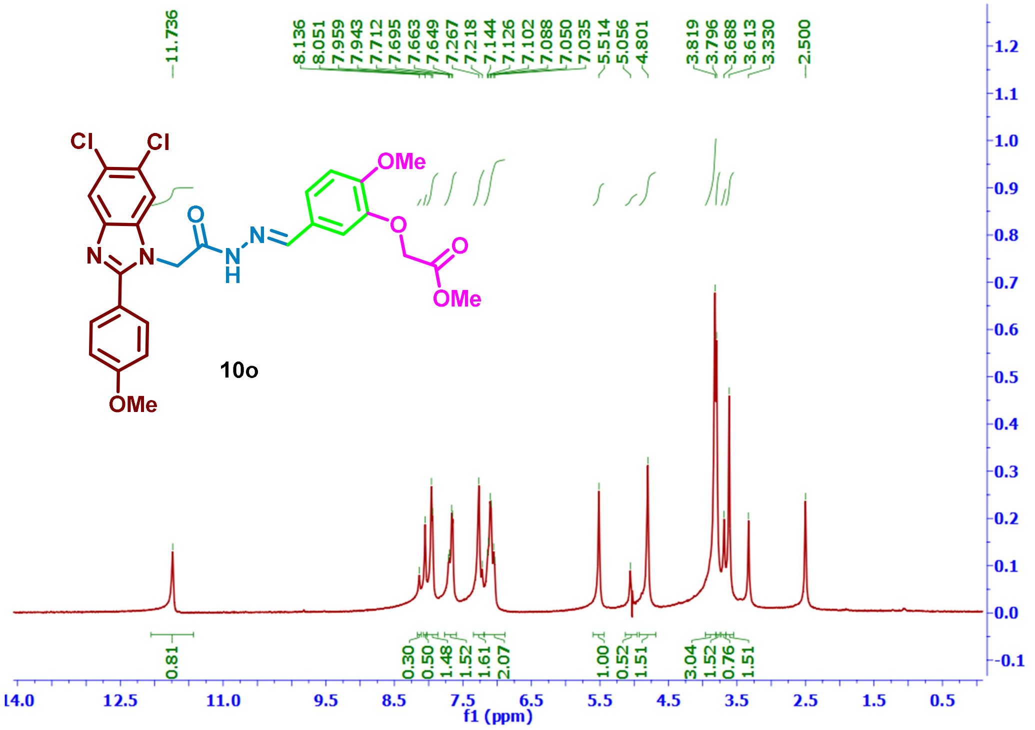

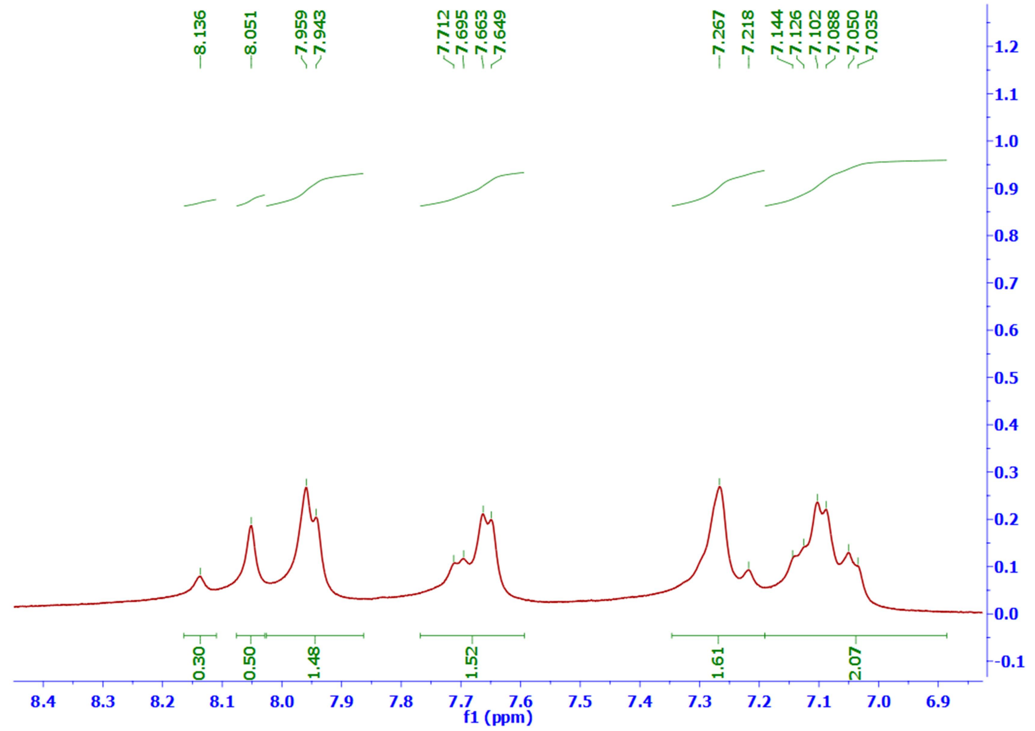

Figure 45. ^{1}H (500 MHz) NMR spectrum of **10o** in DMSO-d_6

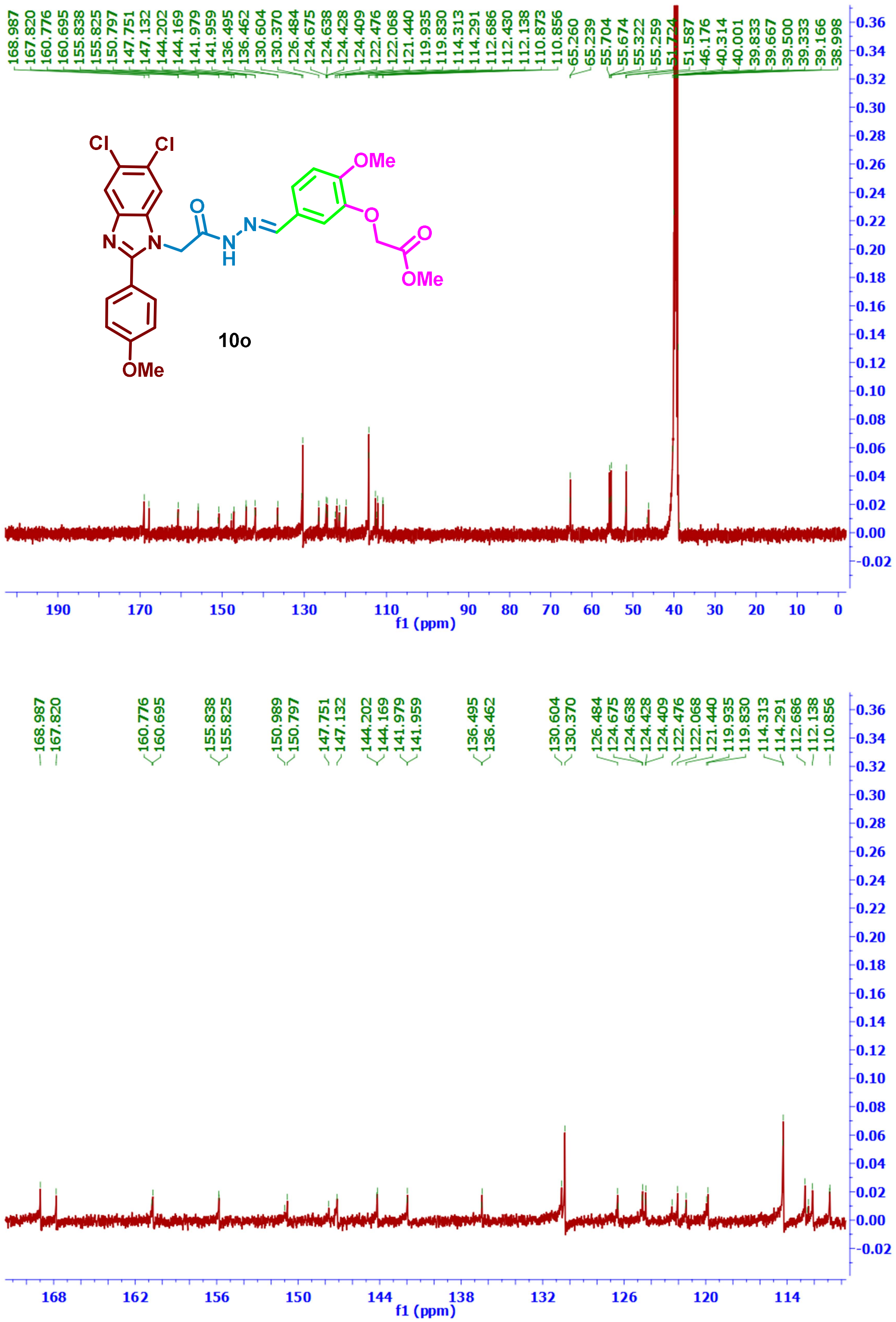

Figure 46. ^{13}C (125 MHz) NMR spectrum of **10o** in DMSO-d_6

Methyl-2-(4-((2-(2-(5,6-dichloro-2-(4-methoxyphenyl)-1*H*-benzo[*d*]imidazol-1-yl)acetyl)hydrazono)methyl)-2-methoxyphenoxy)acetate (10p)

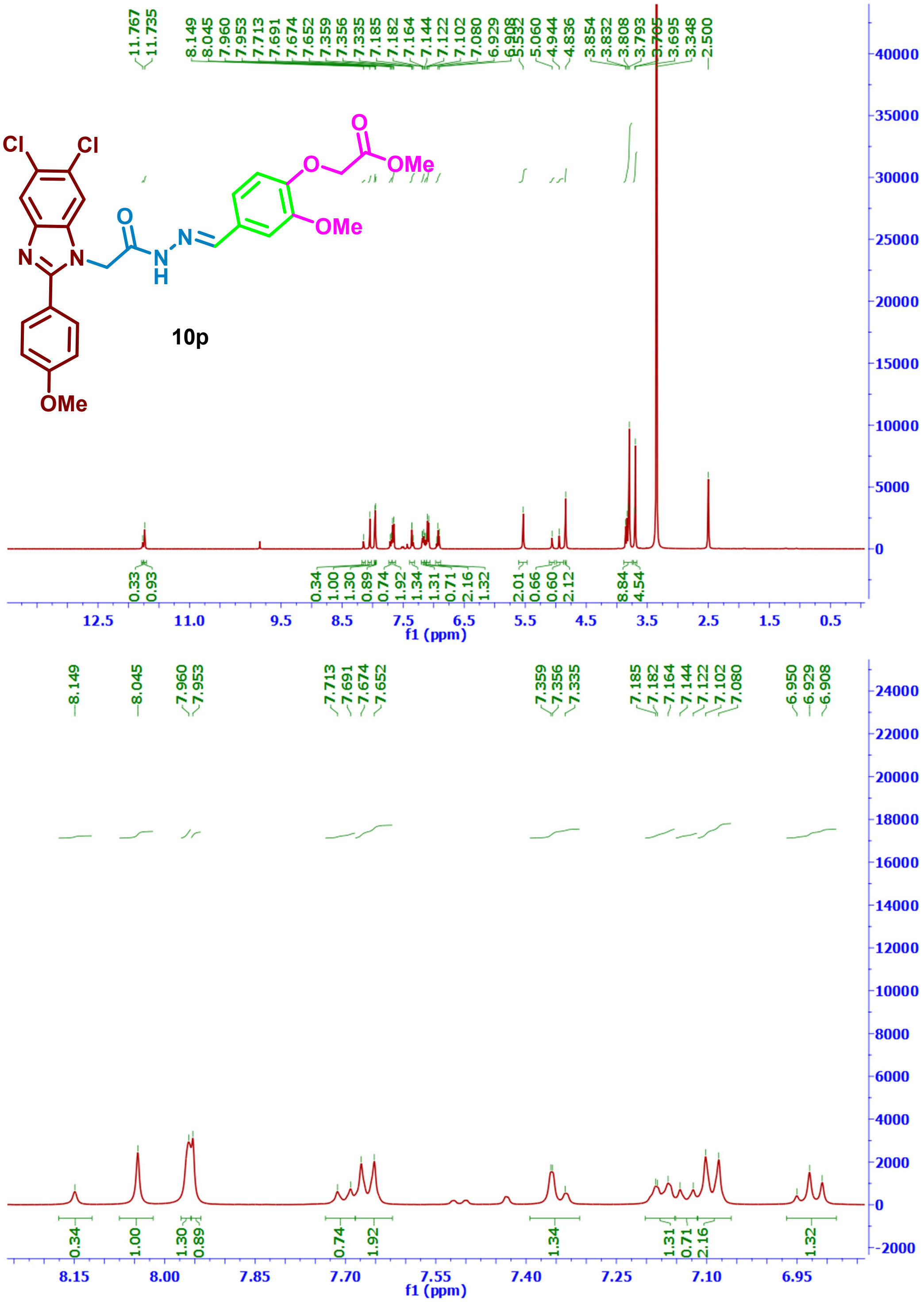

Figure 47. ^{1}H (400 MHz) NMR spectrum of **10p** in DMSO-d_6

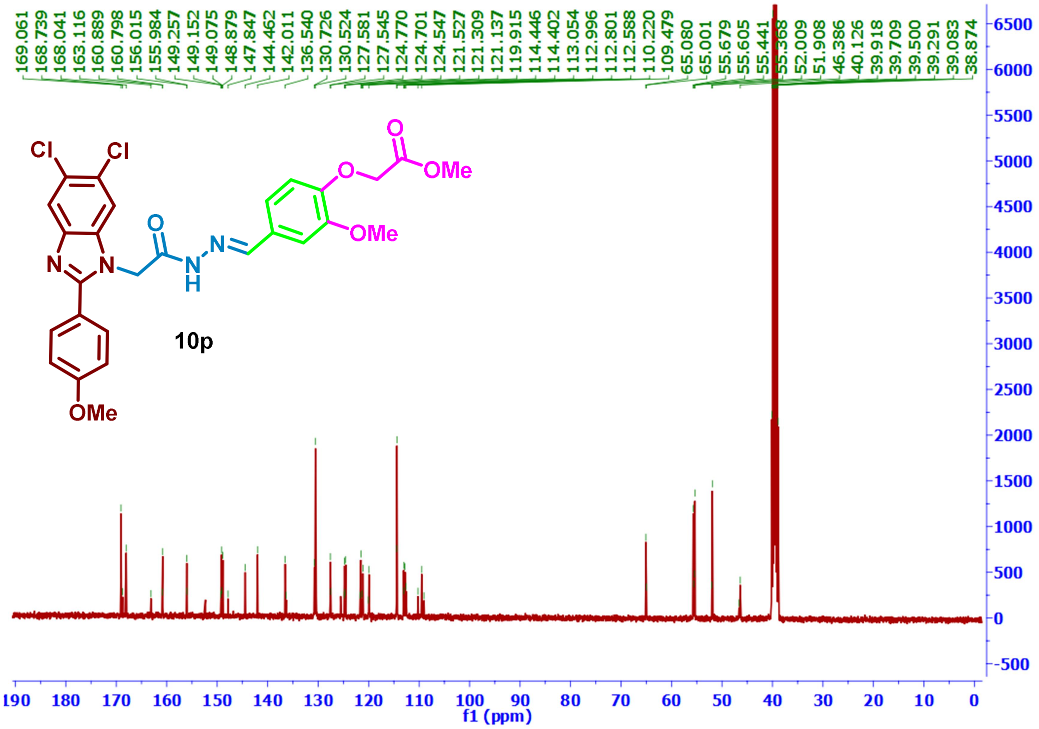

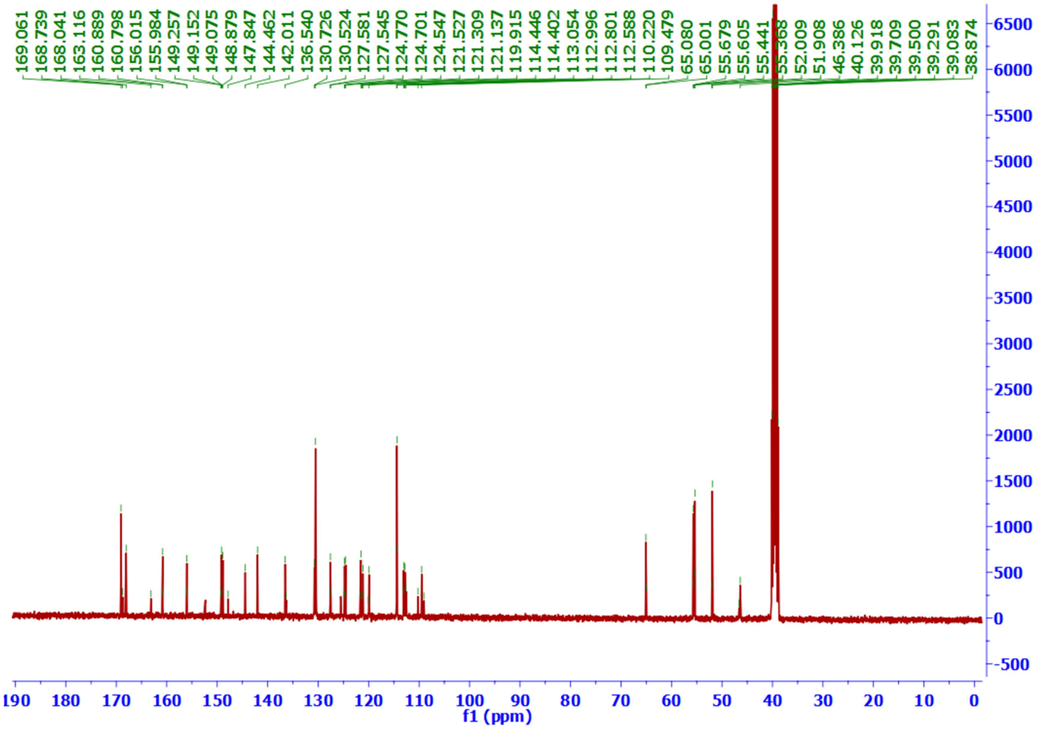

Figure 48. ^{13}C (100 MHz) NMR spectrum of **10p** in DMSO-d_6

3. HRMS spectra of dichlorobenzimidazole derivatives 10a-p

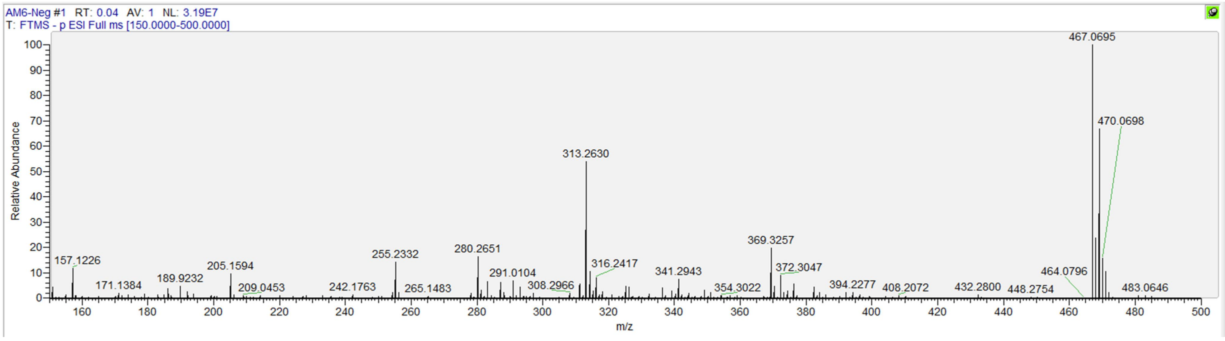

Figure 49. HRMS (-) ESI spectrum of **10a**

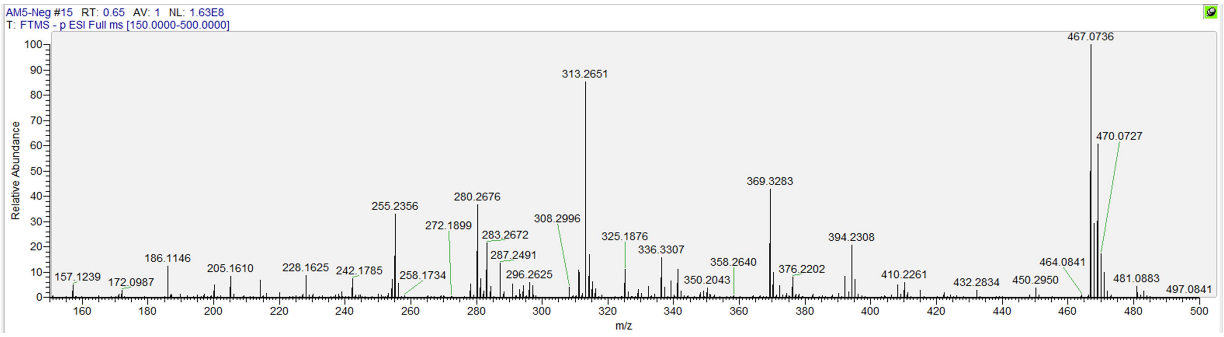

Figure 50. HRMS (-) ESI spectrum of **10b**

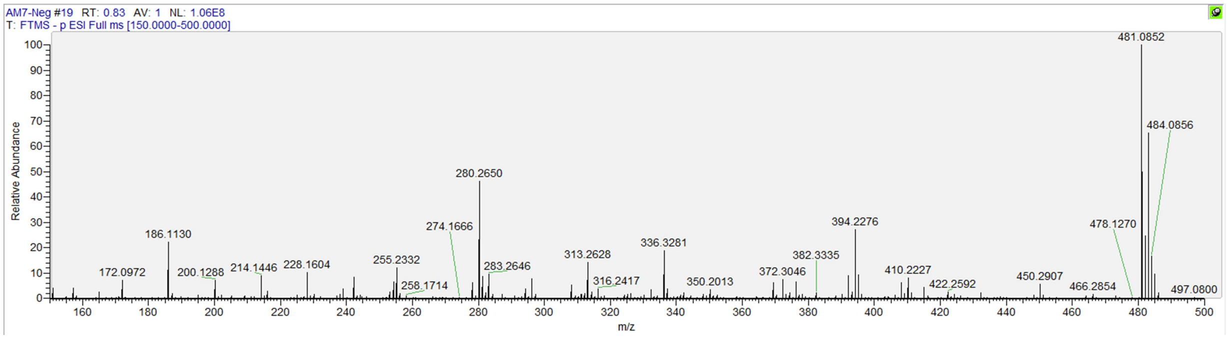

Figure 51. HRMS (-) ESI spectrum of **10c**

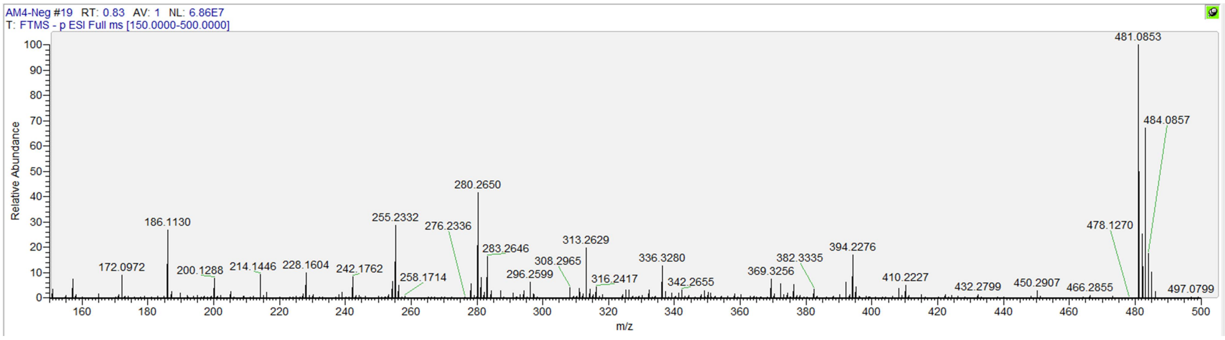

Figure 52. HRMS (-) ESI spectrum of **10d**

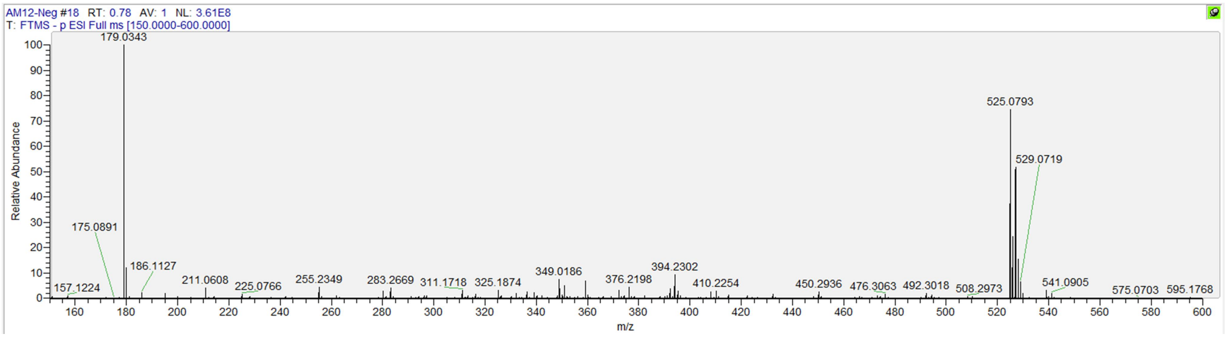

Figure 53. HRMS (-) ESI spectrum of **10e**

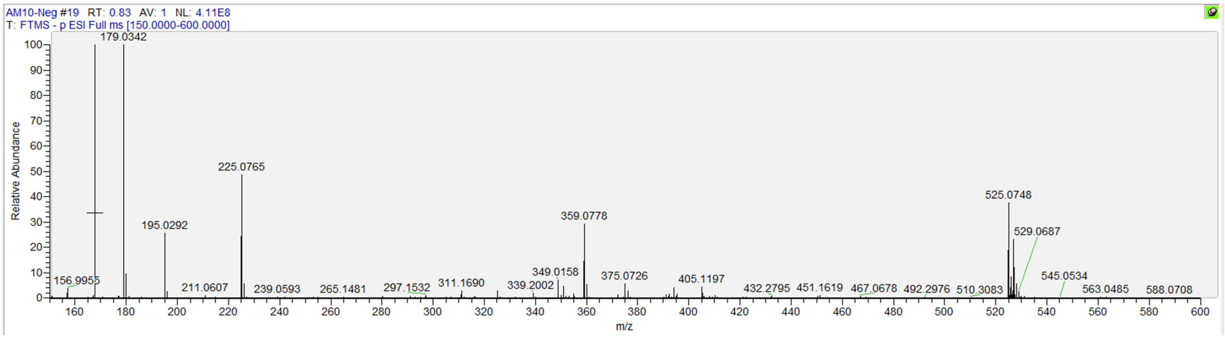

Figure 54. HRMS (-) ESI spectrum of **10f**

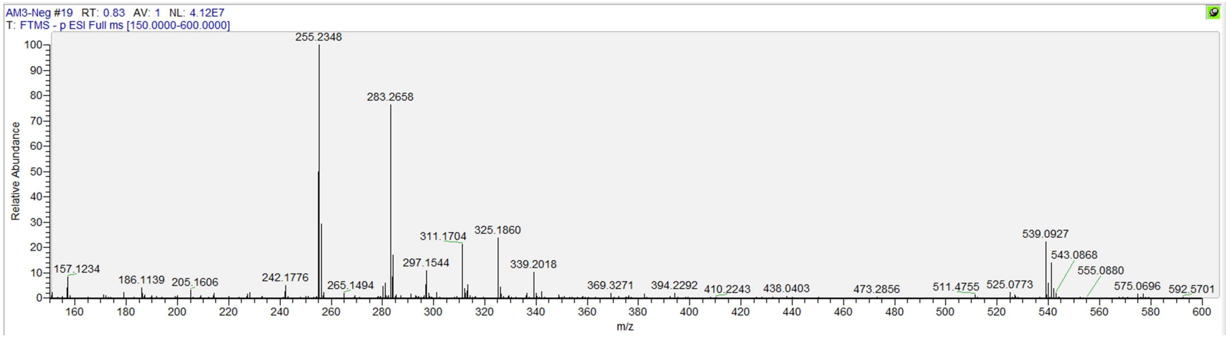

Figure 55. HRMS (-) ESI spectrum of **10h**

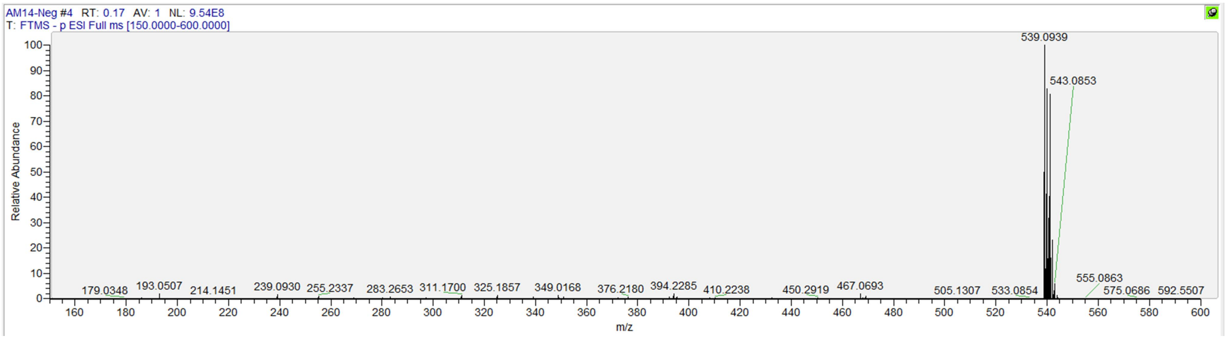

Figure 56. HRMS (-) ESI spectrum of **10i**

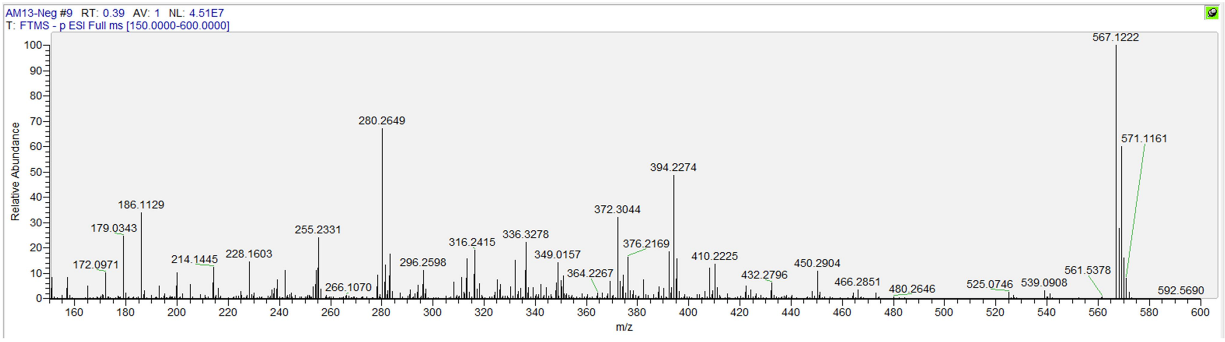

Figure 57. HRMS (-) ESI spectrum of **10j**

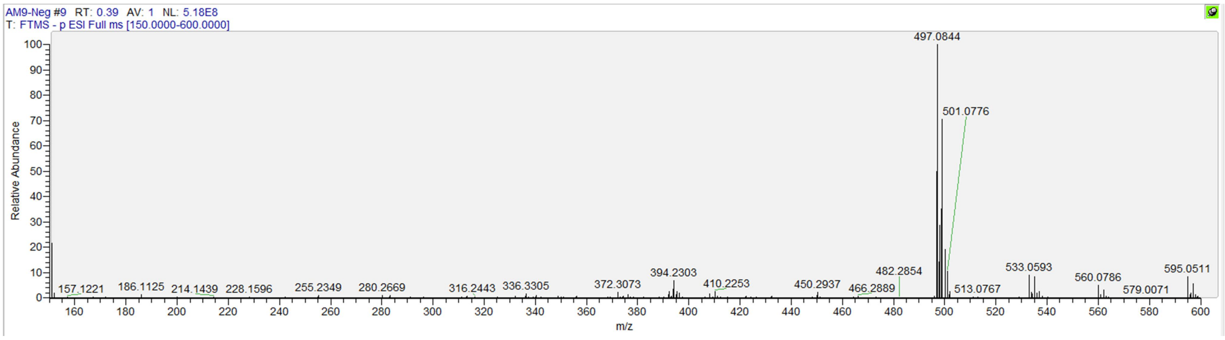

Figure 58. HRMS (-) ESI spectrum of **10k**

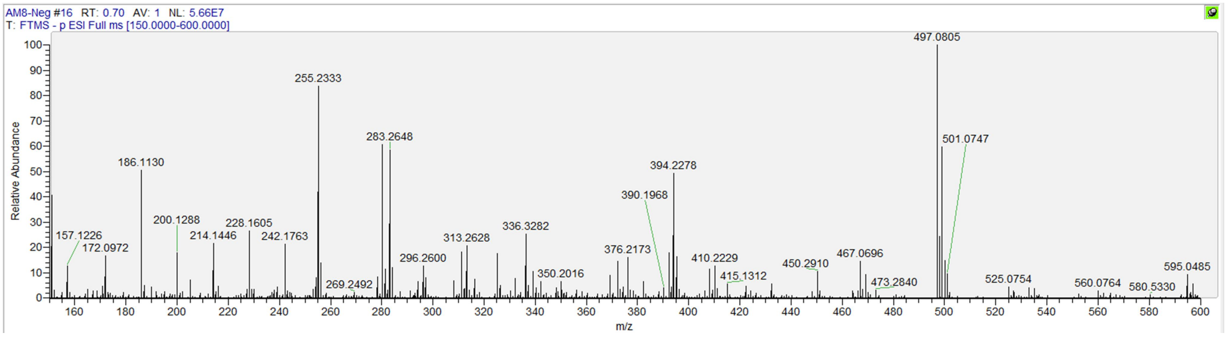

Figure 59. HRMS (-) ESI spectrum of **10l**

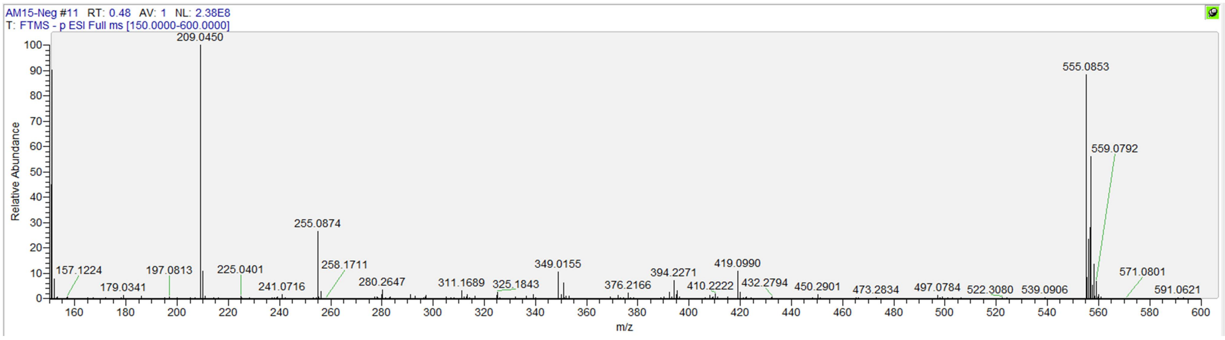

Figure 60. HRMS (-) ESI spectrum of **10m**

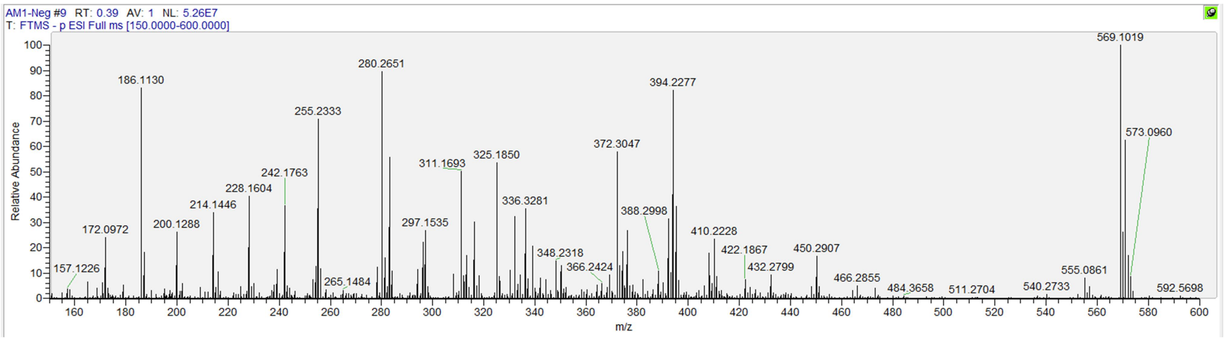

Figure 61. HRMS (-) ESI spectrum of **10o**

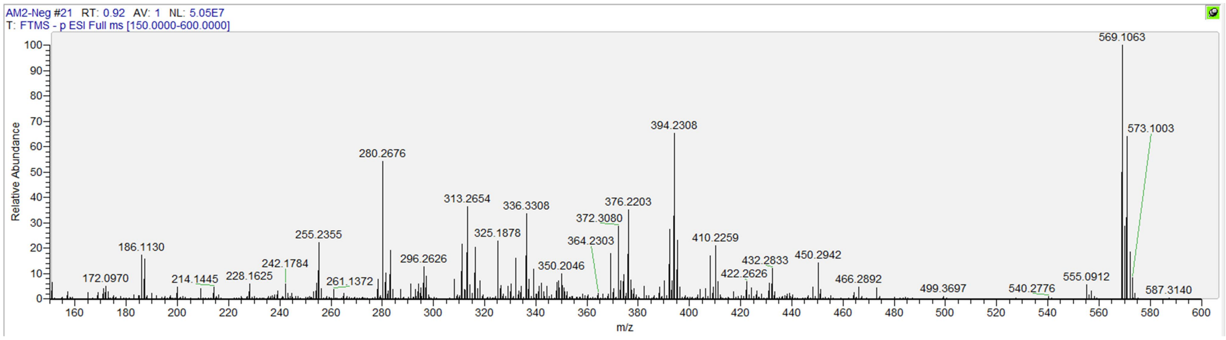

Figure 62. HRMS (-) ESI spectrum of **10p**

4. Biochemical kinase assays

4.1. Procedure

The inhibitory activity against different kinases was determined using kinase kits ($BRAF_{WT}$ cat. ID: 78316) and ($BRAF_{V600E}$ cat. ID: 48688) were purchased from BPS Biosciences and kinase-Glo Max luminescence kinase assay kit (Promega). Sorafenib was used as a reference multi-kinases inhibitor.

The assay was carried out according to the protocol provided by the manufacturer. A stock solution of the synthesized derivatives in 100% DMSO was prepared. Subsequently, the compounds were diluted to 10% DMSO.

A master mixture was prepared according to the number of wells. For BRAF assay each well include 6 µl of 5x Kinase Buffer 1 + 1 µl of ATP (500 µM) + 10 µl of 5X Raf substrate + 8 µl of distilled water. 1x Kinase buffer 1 was prepared by mixing 600 µl of 5x Kinase Buffer 1 with 2400 µl water to give 3 mL. Kinases were thawed on ice and were diluted with 1x Kinase Buffer 1.

To start the biochemical reaction, 25 µL of the master mixture was added to each well in 96 well plate. 5 µL of 10% DMSO was added to positive control wells and blank wells. 5 µL of diluted inhibitor was added to each well labelled with the test inhibitor so that the final concentration of DMSO is 1% in all reactions. Then 20 µL of (2.5 ng/µl) in case of $BRAF_{WT}$ and (1 ng / µl) in case of VEGFR-2 and FGFR-1 in 1x kinase buffer was added to positive control wells and wells labelled with the inhibitor, while 20 µL 1x kinase buffer 1 was added to the blank wells.

The plate was incubated at 30 ^{o}C for 45 min. Subsequently, 50 µl of Kinase-Glo Max luminescence reagent was added to each well and the plate was covered with aluminum foil and incubated at room temperature for 15 min. Finally, the luminescence was recorded using multimode microplate reader. Kinase activity assays were performed in duplicate at each concentration.

The luminescence data were analyzed as follows. The difference between luminescence intensities in the absence of kinase (Lu_t) and in the presence of kinase (Lu_c) was defined as 100 % activity ($Lu_t – Lu_c$). Using luminescence signal (Lu) in the presence of the compound, % activity was calculated as: % activity = $\{(Lu_t - Lu)/(Lu_t - Lu_c)\} \times 100\%$. The concentration of the test compounds required to reduce the kinase activity by 50% was determined from dose-response curves and recorded as their IC_{50}.

5. Screening of cytotoxic activity against a panel of sixty human tumor cell lines

5.1. Procedure

The selected compounds by NCI were evaluated for their anticancer activity in a two-stage process. First, these compounds were screened against the full NCI 60 cell lines panel at a single high dose of 10 μM. Then, the output from the single dose screen is reported as a mean graph. Second, compounds exhibiting significant growth inhibition were evaluated against the 60 cell panel at five different minimal concentrations.

Assay protocol. The human tumor cell lines of the cancer screening panel are grown in RPMI 1640 medium containing 5% fetal bovine serum and 2 mM L-glutamine. For a typical screening experiment, cells are inoculated into 96 well microtiter plates in 100 μL at plating densities ranging from 5000 to 40,000 cells/well depending on the doubling time of individual cell lines. After cell inoculation, the microtiter plates are incubated at 37 ^{o}C, 5% CO_2, 95% air and 100% relative humidity for 24 h prior to addition of the experimental drugs.

After 24 h, two plates of each cell line are fixed in situ with TCA, to represent a measurement of the cell population for each cell line at the time of drug addition (Tz). The experimental drugs are solubilized in dimethyl sulfoxide at 400-fold the desired final maximum test concentration and stored frozen prior to use. At the time of drug addition, an aliquot of frozen concentrate is thawed and diluted to twice the desired final maximum test concentration with complete medium containing 50 μg/mL gentamicin. Additional four, 10-fold or ½ log serial dilutions are made to provide a total of five drug concentrations plus control.

Aliquots of 100 mL of these different drug dilutions are added to the appropriate microtiter wells already containing 100 mL of medium, resulting in the required final drug concentrations. Following drug addition, the plates are incubated for an additional 48 h at 37 ^{o}C, 5% CO_2, 95% air, and 100% relative humidity. For adherent cells, the assay is terminated by the addition of cold TCA. Cells are fixed in situ by the gentle addition of 50 μL of cold 50% (w/v) TCA (final concentration, 10% TCA) and incubated for 60 min at 4 ^{o}C. The supernatant is discarded, and the plates are washed five times with tap water and air dried. Sulforhodamine B (SRB) solution (100 mL) at 0.4% (w/v) in 1% acetic acid is added to each well, and plates are incubated for 10 min at room temperature. After staining, unbound dye is removed by washing five times with 1% acetic acid and the plates are air dried. Bound stain is subsequently solubilized with 10 mM trizma base, and the absorbance is read on an automated plate reader at a wavelength of 515 nm. For suspension cells, the methodology is

the same except that the assay is terminated by fixing settled cells at the bottom of the wells by gently adding 50 µl of 80% TCA (final concentration, 16% TCA). Using the seven absorbance measurements [time zero, (Tz), control growth, (C), and test growth in the presence of drug at the five concentration levels (Ti)], the percentage growth is calculated at each of the drug concentrations levels.

Percentage growth inhibition is calculated as: [(Ti Tz)/(C–Tz)] x 100 for concentrations for which Ti>/=Tz and [(Ti-Tz)/Tz] x 100 for concentrations for which Ti < Tz.

Three dose response parameters are calculated for each experimental agent. Growth inhibition of 50% (GI_{50}) is calculated from [(Ti -Tz)/(C–Tz)] x 100 = 50, which is the drug concentration resulting in a 50% reduction in the net protein increase (as measured by SRB staining) in control cells during the drug incubation. The drug concentration resulting in total growth inhibition (TGI) is calculated from Ti = Tz. The LC_{50} (concentration of drug resulting in a 50% reduction in the measured protein at the end of the drug treatment as compared to that at the beginning) indicating a net loss of cells following treatment is calculated from [(Ti–Tz)/Tz] x 100 = -50. Values are calculated for each of these three parameters if the level of activity is reached; however, if the effect is not reached or is exceeded, the value for that parameter is expressed as greater or less than the maximum or minimum concentration tested. Results for each compound were reported as a mean graph of the percent growth of the treated cells when compared to the untreated control cells. There after obtaining the results for one dose assay, analysis of historical Development Therapeutics Programme (DTP) was performed and compounds which satisfies predetermined threshold inhibition criteria is selected for NCI full panel 5 dose assay.

5.2. Dose response curves of the dichlorobenzimidazoles 10h, 10o and 10p on NCI cancer cell lines

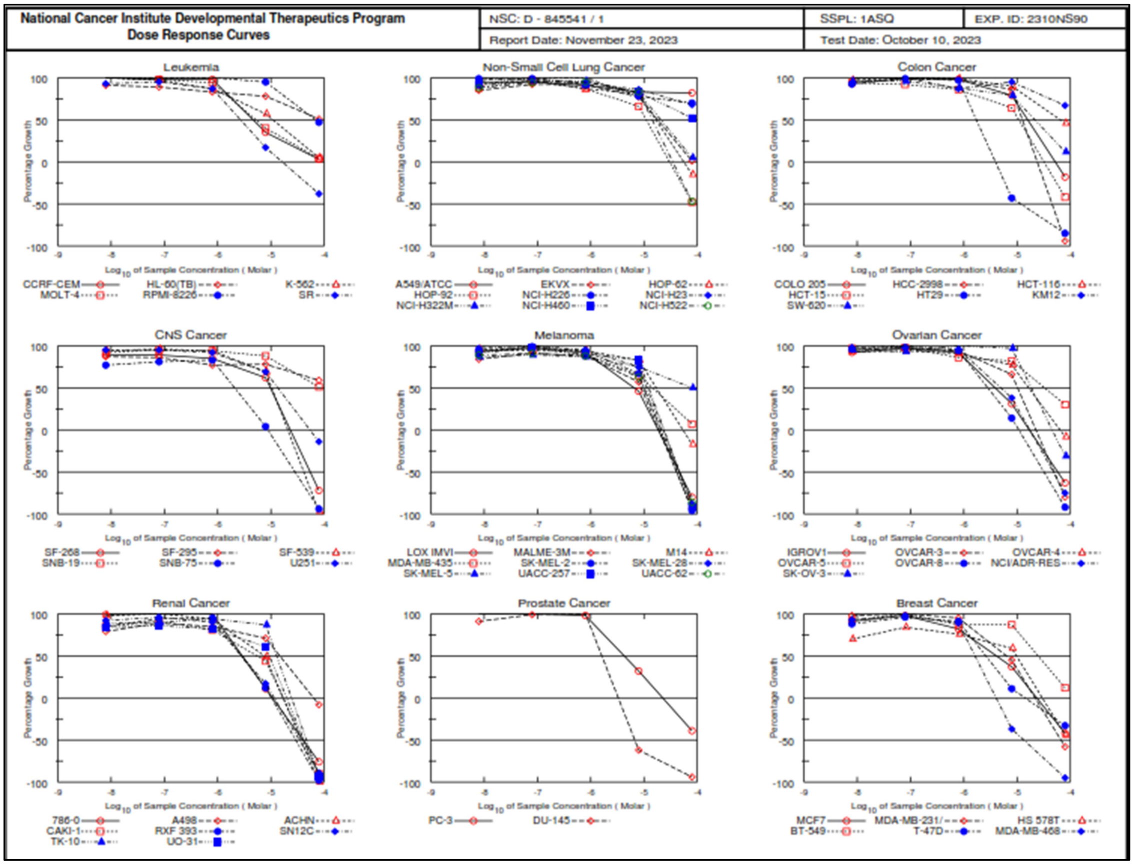

Figure 63. Dose response curves of **10h** on NCI cancer cell lines

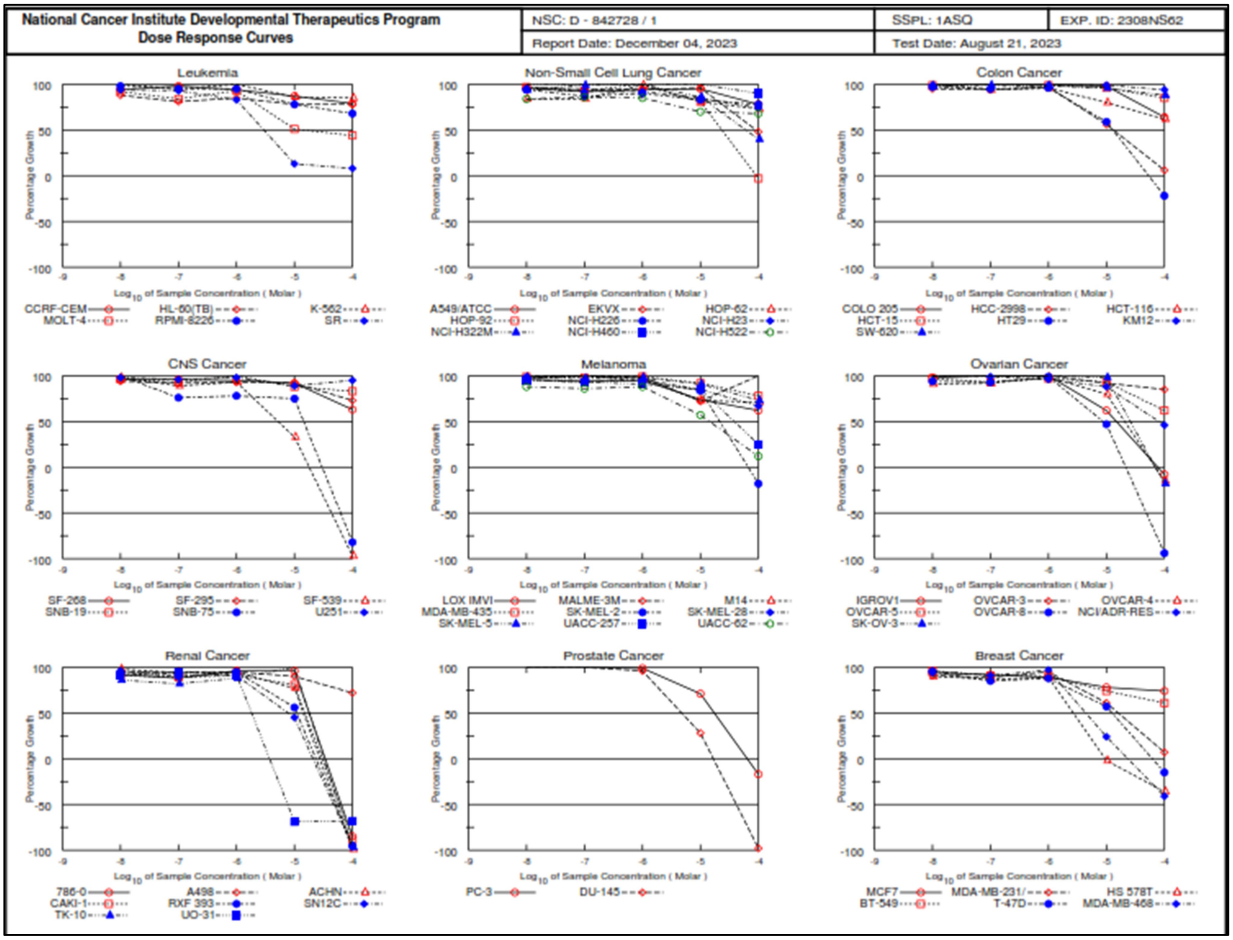

Figure 64. Dose response curves of **10o** on NCI cancer cell lines

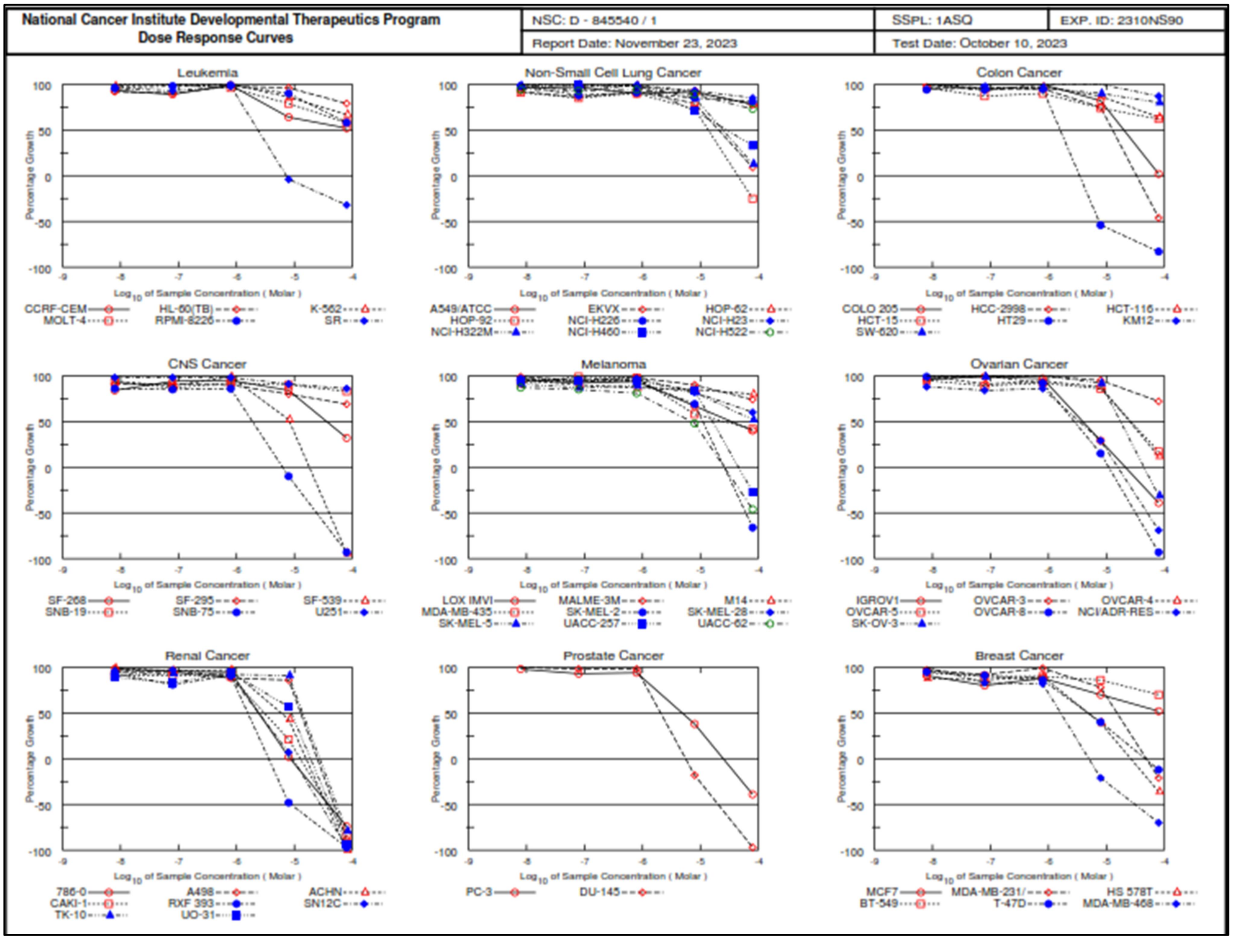

Figure 65. Dose response curves of **10p** on NCI cancer cell lines

6. Analysis of 10h on HSF normal cell line

6.1 Procedure

Human Skin Fibroblast (HSF) was obtained from Nawah Scientific Inc., (Mokatam, Cairo, Egypt). Cells were maintained in DMEM media supplemented with 100 mg/mL of streptomycin, 100 units/mL of penicillin and 10% of heat-inactivated fetal bovine serum in humidified, 5% (v/v) CO2atmosphere at 37 °C. Cell viability was assessed by SRB assay. Aliquots of 100 μL cell suspension (5x10^3 cells) were in 96-well plates and incubated in complete media for 24 h. Cells were treated with another aliquot of 100 μL media containing drugs at various concentrations. After drug exposure, cells were fixed by replacing media with 150 μL of 10% TCA and incubated at 4 °C for 1 h. The TCA solution was removed, and the cells were washed 5 times with distilled water. Aliquots of 70 μL SRB solution (0.4% w/v) were added and incubated in a dark place at room temperature for 10 min. Plates were washed 3 times with 1% acetic acid and allowed to air-dry overnight. Then, 150 μL of TRIS (10 mM) was added to dissolve protein-bound SRB stain; the absorbance was measured at 540 nm using an Infinite F50 microplate reader (TECAN, Switzerland).

6.2. Dose response curve of 10h on HSF normal cell line

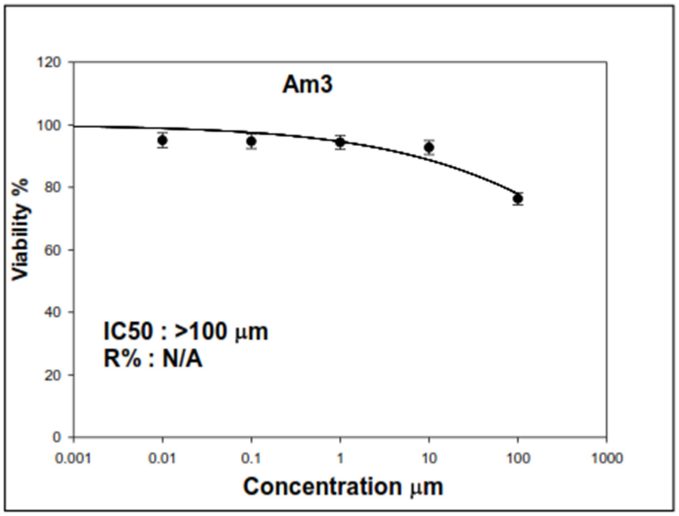

Figure 66. Dose response curve of **10h** on HSF normal cell line

7. Analysis of cell cycle distribution

HT29: Colon Cancer was obtained from Nawah Scientific Inc., (Mokatam, Cairo, Egypt). Cells were maintained in DMEM media supplemented with 100 mg/mL of streptomycin, 100 units/mL of penicillin and 10% of heat-inactivated fetal bovine serum in humidified, 5% (v/v) CO_2 atmosphere at 37 °C. After treatment with test compound **8m** for 48h, cells (105 cells) are collected by trypsinization and washed twice with ice-cold PBS (pH 7.4). Cells are re-suspended in two milliliters of 60% ice-cold ethanol and incubated at 4ºC for 1h for fixation. Fixed cells are washed twice again with PBS (pH 7.4) and re-suspended in 1 mL of PBS containing 50 µg/mL RNAase A and 10 µg/mL propidium iodide (PI). After 20 min of incubation in dark at 37 ^{o}C, cells are analyzed for DNA contents using flow cytometry analysis using FL2 (λex/em 535/617 nm) signal detector (ACEA Novocyte™ flowcytometer, ACEA Biosciences Inc., San Diego, CA, USA). For each sample, 12,000 events are acquired. Cell cycle distribution is calculated using ACEA NovoExpress™ software (ACEA Biosciences Inc., San Diego, CA, USA).

8. Apoptosis assay

Apoptosis and necrosis cell populations are determined using Annexin V-FITC apoptosis detection kit (Abcam Inc., Cambridge Science Park, Cambridge, UK) coupled with 2 fluorescent channels flowcytometry. After treatment with test compounds for 48h, cells (105 cells) are collected by trypsinization and washed twice with ice-cold PBS (pH 7.4). Then, cells are incubated in dark with 0.5 ml of Annexin V-FITC/PI solution for 30 min in dark at room temperature according to manufacturer protocol. After staining, cells are injected via ACEA Novocyte™ flowcytometer (ACEA Biosciences Inc., San Diego, CA, USA) and analysed for FITC and PI fluorescent signals using FL1 and FL2 signal detector, respectively (λex/em 488/530 nm for FITC and λex/em 535/617 nm for PI). For each sample, 12,000 events are acquired and positive FITC and/or PI cells are quantified by quadrant analysis and calculated using ACEA NovoExpress™ software (ACEA Biosciences Inc., San Diego, CA, USA).

9. Molecular Modeling

9.1. Molecular docking:

All the molecular docking simulations were conducted utilizing Molecular Operating Environment (MOE, 2022.02) software. All minimizations were conducted with MOE until an RMSD gradient of 0.05 kcal·mol^{-1}Å^{-2} with Amber14:EHT force field and the partial charges were automatically calculated.

BRAF

For BRAF crystal structure (PDB ID: 1UWH) [1], chain B, water molecules and ligands which are not involved in binding were first removed. Then, protein structure was prepared for the docking study using *QuickPrep* protocol in MOE with default options. The co-crystalized ligand was used to define the active site in the protein structure for docking. *Triangle Matcher* placement method and *London dG* scoring function were used for docking utilizing *induced fit* protocol. Docking protocol was first validated by self-docking of the co-crystallized ligand (Sorafenib) in the vicinity of the active site giving a docking pose with an energy score (S) = −14.98 kcal/mol and an RMSD of 0.730Å from the co-crystalized ligand pose (**Figure 67**).

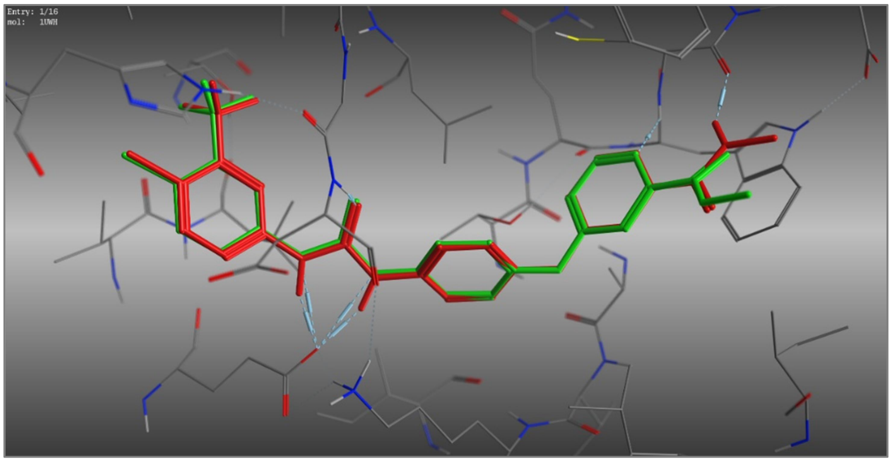

(A)

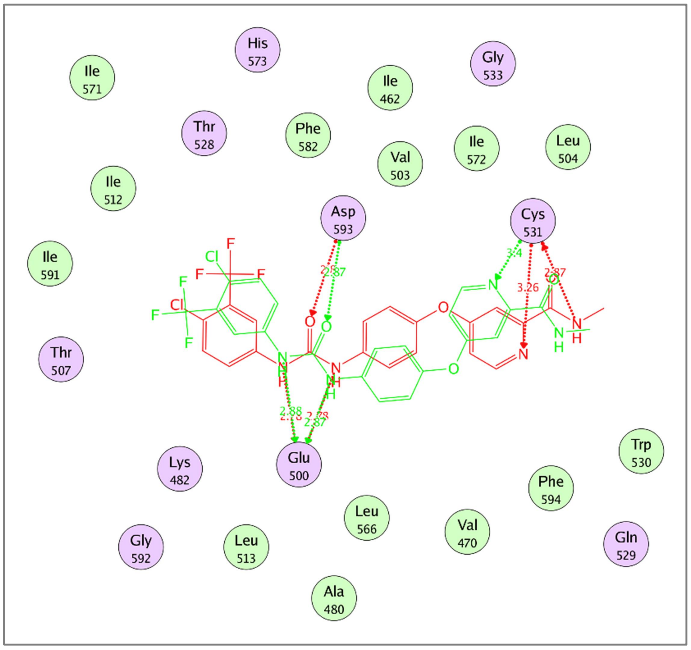

(B)

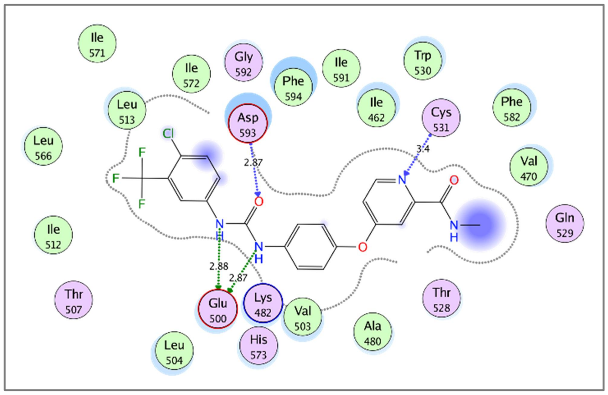

(C)

Figure 67. 2D diagram (A) and 3D representation (B) of the superimposition of docking pose (green) and the co-crystallized (red) of Sorafenib in the BRAF active site with RMSD of 0.730Å. (C) 2D interaction diagram showing Sorafenib docking pose interactions with the key amino acids (hot spots) in the BRAF active site. (Distances in Å)

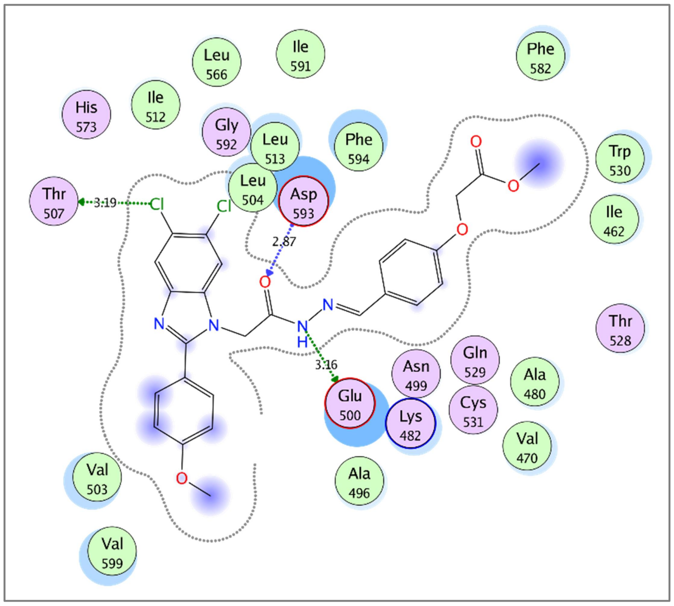

Figure 68. 2D interaction diagram showing compound **10h** docking pose interactions with the key amino acids (hot spots) in the BRAF active site. (Distances in Å)

BRAF$_{V600E}$

For BRAF$_{V600E}$ crystal structure (PDB ID: 1UWJ) [1], chain B, a water molecule and ligands which are not involved in binding were first removed. Then, protein structure was prepared for the docking study using *QuickPrep* protocol in MOE with default options. The co-crystalized ligand was used to define the active site in the protein structure for docking. *Triangle Matcher* placement method and *London dG* scoring function were used for docking utilizing *induced fit* protocol. Docking protocol was first validated by self-docking of the co-crystallized ligand (Sorafenib) in the vicinity of the active site giving a docking pose with an energy score (S) = −15.36 kcal/mol and an RMSD of 0.720Å from the co-crystalized ligand pose (**Figure 69**).

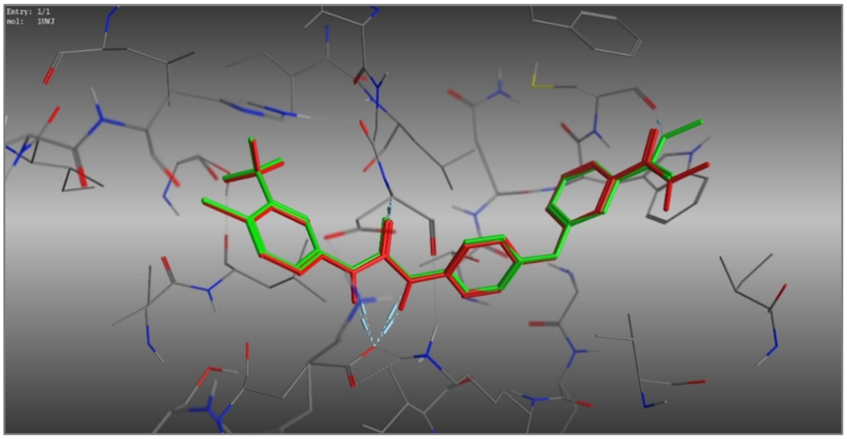

(A)

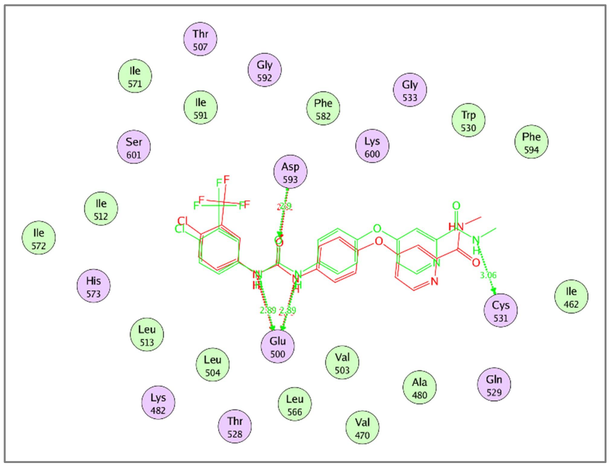

(B)

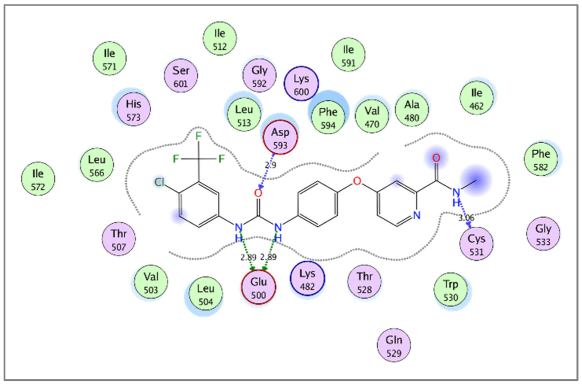

(C)

Figure 69. 2D diagram (A) and 3D representation (B) of the superimposition of docking pose (green) and the co-crystallized (red) of Sorafenib in the $BRAF_{V600E}$ active site with RMSD of 0.720Å. (C) 2D interaction diagram showing Sorafenib docking pose interactions with the key amino acids (hot spots) in the $BRAF_{V600E}$ active site (Distances in Å)

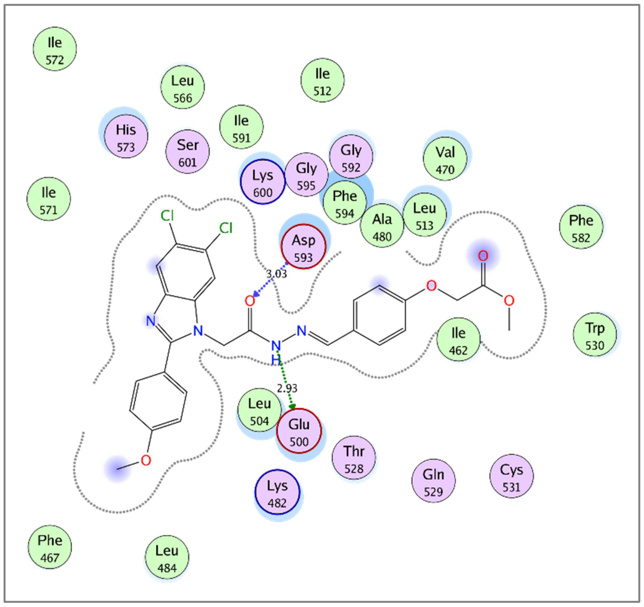

Figure 70. 2D interaction diagram showing compound **10h** docking pose interactions with the key amino acids (hot spots) in the $BRAF_{V600E}$ active site (Distances in Å)

Table 1. Docking energy scores (*S*) in kcal/mol for the most potent compound **10h** and the co-crystalized compound (Sorafenib) in BRAF and $BRAF_{V600E}$ active sites

**Compound**	**Energy score (S) kcal/mol BRAF**	**Energy score (S) kcal/mol $BRAF_{V600E}$**
**10h**	−16.02	−17.30
**Co-crystalized ligand (Sorafenib)**	−14.98	−15.36

9.2. Molecular dynamic simulations

To further investigate the binding pattern and the dynamic behaviour of the newly synthesized compounds, molecular dynamics (MD) simulations for compound **10h**, as a representative compound, in BRAF and $BRAF_{V600E}$ kinase domains were carried out. Starting from the obtained molecular docking **10h**/BRAF and **10h**/$BRAF_{V600E}$ complexes, MD simulations were performed using Groningen Machine for Chemical Simulations (GROMACS) 2021.3 package [2]. Amber99SB force field was used for protein topology generation [3]. Whereas ligand parametrization was carried out using Amber GAFF force field [4, 5], followed by topology generation using ACPYPE (AnteChamber Python Parser Interface) [6]. Solvation was carried out in a triclinic box (1 nm in all directions from the protein) using TIP3P water model which was neutralized with six Cl^- ions in **10h**/BRAF complex and seven Cl^- ions in **10h**/$BRAF_{V600E}$ complex. System energy minimization was first performed using steepest descent algorithm until it converged to F_{max} less than 1000 kJ mol^{-1} nm^{-1}. Then system equilibration was carried out under NVT followed by NPT ensembles, 100 ps each, with the protein atomic positions restrained. In the NVT step, the modified Berendsen (V-rescale) thermostat with a time constant of 0.1 ps was used to keep the temperature at 300 K [7]. In the NPT step, the pressure was maintained at 1 bar using the Berendsen pressure coupling method (isotropic coupling type) with a time constant of 2 ps [8]. Finally, full production simulations for 100 ns were run using the leap-frog integrator with a timestep of 2 fs. During the production runs, Linear Constraint Solver (LINCS) algorithm, V-rescale thermostat with a time constant of 0.1 ps, and Parrinello−Rahman barostat with a time constant of 2 ps were used [7, 9, 10]. Particle Mesh Ewald summation (PME) method was used for long-range electrostatics description [11]. Long-range electrostatic and short-range van der Waals cut-off was set to 1 nm. Trajectories were recorded after every 10.0 ps. For both complexes, the Root Mean Square Deviation (RMSD) from the initial reference frame backbone was calculated for the backbone Cα atom and was graphically analysed at a time point scale in ns [12, 13]. Furthermore, root mean square fluctuation (RMSF) for each residue was also calculated [14]. Radius of gyration (Rg) was also calculated to understand the compactness of **10h**/BRAF and **10h**/$BRAF_{V600E}$ complexes [15]. The analysis of the resulting trajectories was performed using GROMACS tools and Chimera 1.17.1 [16]. The obtained poses of compound **10h** in BRAF and $BRAF_{V600E}$ kinase domains in the dominant clusters were scored using Molecular Mechanics/Generalized Born Surface Area (MM/GBSA) binding free energy calculation method implemented in fastDRH webserver (http://cadd.zju.edu.cn/fastdrh/) [17] and were compared to that of the co-crystalized ligand sorafenib in its experimental poses. MM/GBSA binding free energy was calculated using ff99SB force field

(with TIP3P water model) for protein and GAFF2 force field for the ligands implemented in fastDRH webserver (http://cadd.zju.edu.cn/fastdrh/) [17].

10. References

[1] P.T. Wan, M.J. Garnett, S.M. Roe, S. Lee, D. Niculescu-Duvaz, V.M. Good, C.M. Jones, C.J. Marshall, C.J. Springer, D. Barford, R. Marais, P. Cancer Genome, Mechanism of activation of the RAF-ERK signaling pathway by oncogenic mutations of B-RAF, Cell 116 (2004) 855-67.

[2] M.J. Abraham, T. Murtola, R. Schulz, S. Páll, J.C. Smith, B. Hess, E. Lindahl, GROMACS: High performance molecular simulations through multi-level parallelism from laptops to supercomputers, SoftwareX 1-2 (2015) 19-25.

[3] K. Lindorff-Larsen, S. Piana, K. Palmo, P. Maragakis, J.L. Klepeis, R.O. Dror, D.E. Shaw, Improved side-chain torsion potentials for the Amber ff99SB protein force field, Proteins 78 (2010) 1950-1958.

[4] J. Wang, R.M. Wolf, J.W. Caldwell, P.A. Kollman, D.A. Case, Development and testing of a general amber force field, J Comput Chem 25 (2004) 1157-1174.

[5] J. Wang, W. Wang, P.A. Kollman, D.A. Case, Automatic atom type and bond type perception in molecular mechanical calculations, J Mol Graph Model 25 (2006) 247-260.

[6] A.W. Sousa da Silva, W.F. Vranken, ACPYPE - AnteChamber PYthon Parser interfacE, BMC Res Notes 5 (2012) 367.

[7] G. Bussi, D. Donadio, M. Parrinello, Canonical sampling through velocity rescaling, J Chem Phys 126 (2007) 014101.

[8] H.J.C. Berendsen, J.P.M. Postma, W.F. van Gunsteren, A. DiNola, J.R. Haak, Molecular dynamics with coupling to an external bath, J Chem Phys 81 (1984) 3684-3690.

[9] B. Hess, H. Bekker, H. Berendsen, J. Fraaije, LINCS: A Linear Constraint Solver for molecular simulations, J Comput Chem 18 (1997) 14631472.

[10] M. Parrinello, A. Rahman, Polymorphic transitions in single crystals: A new molecular dynamics method, J Appl Phys 52 (1981) 7182-7190.

[11] T. Darden, D. York, L. Pedersen, Particle mesh Ewald: An N·log(N) method for Ewald sums in large systems, J Chem Phys 98 (1993) 10089-10092.

[12] K.L. Damm, H.A. Carlson, Gaussian-weighted RMSD superposition of proteins: a structural comparison for flexible proteins and predicted protein structures, Biophysical journal 90 (2006) 4558-73.

[13] V.N. Maiorov, G.M. Crippen, Significance of Root-Mean-Square Deviation in Comparing Three-dimensional Structures of Globular Proteins, J Mol Biol 235 (1994) 625-634.

[14] E. Fuglebakk, J. Echave, N. Reuter, Measuring and comparing structural fluctuation patterns in large protein datasets, Bioinformatics 28 (2012) 2431-2440.

[15] M.Y. Lobanov, N.S. Bogatyreva, O.V. Galzitskaya, Radius of gyration as an indicator of protein structure compactness, Mol Biol 42 (2008) 623-628.
[16] E.F. Pettersen, T.D. Goddard, C.C. Huang, G.S. Couch, D.M. Greenblatt, E.C. Meng, T.E. Ferrin, UCSF Chimera—A visualization system for exploratory research and analysis, J Comput Chem 25 (2004) 1605-1612.
[17] Z. Wang, H. Pan, H. Sun, Y. Kang, H. Liu, D. Cao, T. Hou, fastDRH: a webserver to predict and analyze protein-ligand complexes based on molecular docking and MM/PB(GB)SA computation, Brief Bioinform 23 (2022)
